# Spatial and temporal fluctuations in COVID-19 fatality rates in Brazilian hospitals

**DOI:** 10.1038/s41591-022-01807-1

**Published:** 2022-05-10

**Authors:** Andrea Brizzi, Charles Whittaker, Luciana M. S. Servo, Iwona Hawryluk, Carlos A. Prete, William M. de Souza, Renato S. Aguiar, Leonardo J. T. Araujo, Leonardo S. Bastos, Alexandra Blenkinsop, Lewis F. Buss, Darlan Candido, Marcia C. Castro, Silvia F. Costa, Julio Croda, Andreza Aruska de Souza Santos, Christopher Dye, Seth Flaxman, Paula L. C. Fonseca, Victor E. V. Geddes, Bernardo Gutierrez, Philippe Lemey, Anna S. Levin, Thomas Mellan, Diego M. Bonfim, Xenia Miscouridou, Swapnil Mishra, Mélodie Monod, Filipe R. R. Moreira, Bruce Nelson, Rafael H. M. Pereira, Otavio Ranzani, Ricardo P. Schnekenberg, Elizaveta Semenova, Raphael Sonabend, Renan P. Souza, Xiaoyue Xi, Ester C. Sabino, Nuno R. Faria, Samir Bhatt, Oliver Ratmann

**Affiliations:** 1 Department of Mathematics, Imperial College London, London, UK; 2 MRC Centre for Global Infectious Disease Analysis, Jameel Institute, School of Public Health, Imperial College London, London, UK; 3 Institute for Applied Economic Research, IPEA, Brasília, Brazil; 4 Departamento de Engenharia de Sistemas Eletrônicos, Escola Politécnica, Universidade de São Paulo, São Paulo, Brazil; 5 World Reference Center for Emerging Viruses and Arboviruses and Department of Microbiology and Immunology, University of Texas Medical Branch, Galveston TX, USA; 6 Departamento de Genética, Ecologia e Evolução, Instituto de Ciências Biológicas, Universidade Federal de Minas Gerais, Belo Horizonte, Brazil; 7 Instituto D’Or de Pesquisa e Ensino (IDOR), Rio de Janeiro, Brazil; 8 Laboratory of Quantitative Pathology, Center of Pathology, Adolfo Lutz Institute, São Paulo, Brazil; 9 Programa de Computação Científica, Fundação Oswaldo Cruz, Rio de Janeiro, Brazil; 10 Departamento de Moléstias Infecciosas e Parasitárias e Instituto de Medicina Tropical da Faculdade de Medicina, Universidade de São Paulo, São Paulo, Brazil; 11 Department of Zoology, University of Oxford, Oxford, UK; 12 Department of Global Health and Population, Harvard T. H. Chan School of Public Health, Boston MA, USA; 13 Department of Epidemiology of Microbial Diseases, Yale School of Public Health, New Haven CT, USA; 14 Latin American Centre, University of Oxford, Oxford, UK; 15 Department of Computer Science, University of Oxford, Oxford, UK; 16 Department of Microbiology, Immunology and Transplantation, Rega Institute, KU Leuven – University of Leuven, Leuven, Belgium; 17 Section of Epidemiology, School of Public Health, University of Copenhagen, Copenhagen, Denmark; 18 Departamento de Genética, Instituto de Biologia, Universidade Federal do Rio de Janeiro, Rio de Janeiro, Brazil; 19 Environmental Dynamics, INPA, National Institute for Amazon Research, Manaus, Brazil; 20 Barcelona Institute for Global Health, ISGlobal, Barcelona, Spain; 21 Nuffield Department of Clinical Neurosciences, University of Oxford, Oxford, UK; 22 Department of Infectious Disease Epidemiology, Imperial College London, London, UK

**Keywords:** Health care, Developing world

## Abstract

The severe acute respiratory syndrome coronavirus 2 (SARS-CoV-2) Gamma variant of concern has spread rapidly across Brazil since late 2020, causing substantial infection and death waves. Here we used individual-level patient records after hospitalization with suspected or confirmed coronavirus disease 2019 (COVID-19) between 20 January 2020 and 26 July 2021 to document temporary, sweeping shocks in hospital fatality rates that followed the spread of Gamma across 14 state capitals, during which typically more than half of hospitalized patients aged 70 years and older died. We show that such extensive shocks in COVID-19 in-hospital fatality rates also existed before the detection of Gamma. Using a Bayesian fatality rate model, we found that the geographic and temporal fluctuations in Brazil’s COVID-19 in-hospital fatality rates were primarily associated with geographic inequities and shortages in healthcare capacity. We estimate that approximately half of the COVID-19 deaths in hospitals in the 14 cities could have been avoided without pre-pandemic geographic inequities and without pandemic healthcare pressure. Our results suggest that investments in healthcare resources, healthcare optimization and pandemic preparedness are critical to minimize population-wide mortality and morbidity caused by highly transmissible and deadly pathogens such as SARS-CoV-2, especially in low- and middle-income countries.

## Abstract (web summary)

Analysis of individual-level patient records from Brazil reveals that the extensive shocks in COVID-19 mortality rates are associated with pre-pandemic geographic inequities as well as shortages in healthcare capacity during the pandemic.

## Main

Since late 2020, emerging variants of concern of severe acute respiratory syndrome coronavirus 2 (SARS-CoV-2) have led to substantial epidemic waves and marked increases in coronavirus disease 2019 (COVID-19) deaths^1–5^. In Brazil, the SARS-CoV-2 Gamma variant of concern (also known as P.1, 20J/501Y.V3 or GR/501Y.V3, VOC-21JAN-02 or VOC202101/02) was first detected in December 2020 in Manaus, Amazonas state, in north Brazil^3,6,7^. Gamma is characterized by several amino acid substitutions in the spike protein, including L18F, N501Y, E484K and K417N, that have been associated with increased transmissibility and immune escape^3,8,9^. Three weeks after its detection, Gamma became the dominant lineage circulating in Manaus as measured by variant frequency^3^. The rapid spread of Gamma through the country was followed by waves of COVID-19-associated mortality, suggesting increased disease severity after infection and hospitalization with Gamma^10^. However, these data have not been examined in the context of extensive inequities in baseline development and healthcare capacity across Brazil, which are common in low- and middle-income countries^11^.

**Fig. 1:**
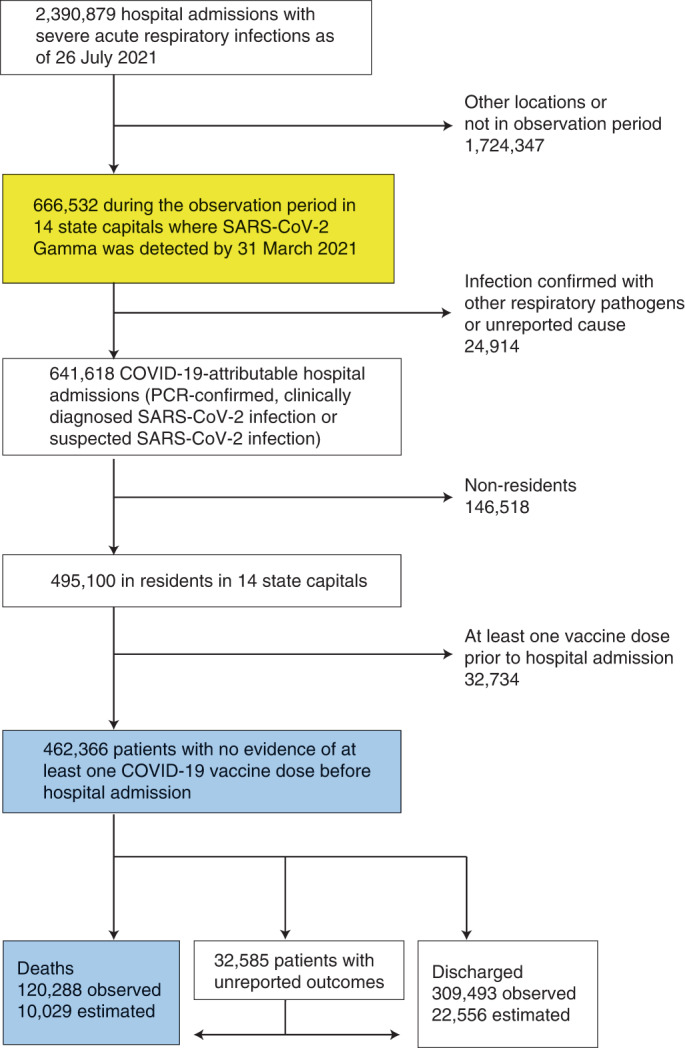
Analysis flow chart. Individual-level records of hospital admissions with severe acute respiratory infection across Brazil are mandatory to report to the SIVEP-Gripe database, and publicly available records between 20 January 2020 and 26 July 2021 were downloaded on 31 January 2022. Data used to derive COVID-19 in-hospital fatality rates are shown in blue, and data used to derive the healthcare pressure indices are shown in yellow (Methods).

In this study, we used individual-level patient histories after hospitalization with suspected or confirmed COVID-19 (Fig. 1)^12,13^ to describe how the expansion of Gamma was followed by shocks in COVID-19 fatality rates in Brazilian hospitals, and we showed that in-hospital fatality rates also fluctuated extensively before the emergence of Gamma. We introduced pandemic healthcare pressure indices that measure and monitor mismatches between healthcare demand and resource availability in hospital settings. We found that these pandemic healthcare pressure indices are strongly correlated with variations in in-hospital COVID-19 fatality rates across 14 Brazilian state capitals. Using a Bayesian model, we then assessed the factors driving the extensive fluctuations in COVID-19 in-hospital fatality rates, from pre-pandemic geographic inequities in economic development, healthcare resources and population vulnerability^14–18^, to pandemic healthcare pressures^15,19,20^ and variant-specific disease severity as measured by in-hospital fatality rates^21,22^. Table 1 summarizes our findings and policy implications.

**Table 1:** Policy summary

Background	Little research has put the evolving COVID-19 pandemic into the wider focus of healthcare inequities and pandemic healthcare pressure in low- and middle-income countries.
Main findings and limitations	For Brazil, we show that COVID-19 fatality rates in hospitals have fluctuated substantially both geographically and temporally since the beginning of the pandemic. In several cities, shock periods are characterized by in-hospital fatality rates exceeding 50% in patients aged 70 years and older. Using healthcare-facility-level microdata on personnel and equipment, we measured healthcare pressure at the city level and found strong associations with the fluctuating COVID-19 in-hospital fatality rates. These associations are confirmed in a Bayesian model that accounts for the emergence and rapid spread of the SARS-CoV-2 Gamma variant. We estimate that approximately half of Brazil’s COVID-19 deaths in hospitals could have been avoided without pre-pandemic geographic inequities and without the multitude of pandemic healthcare pressures. Limitations of this study include sparsely available data on patient comorbidity factors and incomplete data on patient outcomes.
Policy implications	Our findings show that COVID-19 fatality rate shocks seen in Brazilian hospitals can be explained by a substantially increased demand on limited healthcare resources that follow SARS-CoV-2 infection waves. The conclusions are relevant for other countries and show that responding to acute healthcare shocks is challenging. Without sustained investments in healthcare resources, healthcare optimization and pandemic preparedness to minimize population-wide mortality and morbidity, countries will continue to experience high fatality rates caused by immune-escape SARS-CoV-2 variants and other highly transmissible viruses.

## Results

### In-hospital fatality rates fluctuated around the emergence of Gamma

**Fig. 2:**
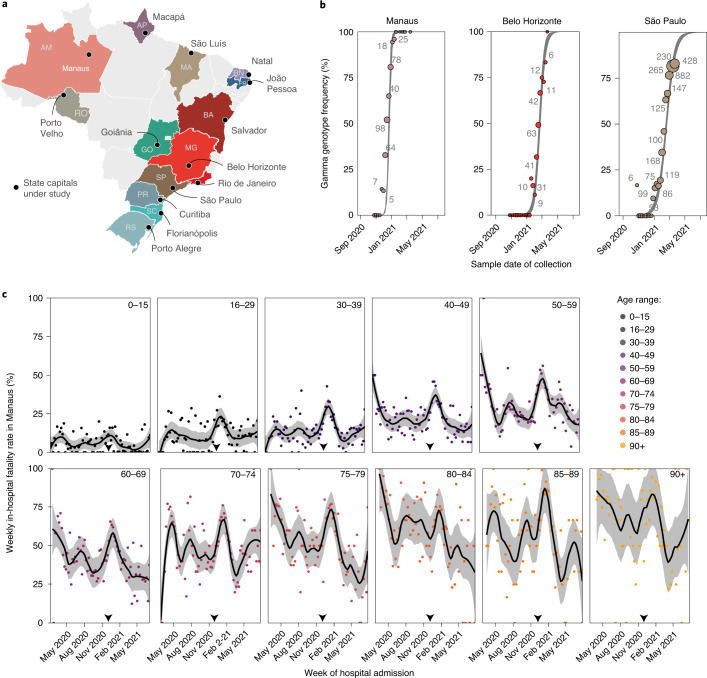
Spatio-temporal expansion of SARS-CoV-2 Gamma in Brazil and associated shocks in COVID-19 fatality rates in hospitals. **a**, The 14 states and state capitals in which Gamma was detected by 31 March 2021 and which were included in the analysis. **b**, Time evolution of SARS-CoV-2 Gamma variant frequencies in three locations, suggesting rapid expansion. Data from GISAID^23^ (dots) are shown along with the number of sequenced SARS-CoV-2 samples (text) and posterior median model fits (line) and associated 95% CrIs (gray ribbon). **c**, Weekly COVID-19 in-hospital fatality rates among hospitalized residents in Manaus with no evidence of vaccination before admission (dots), by age group (facets). Non-parametric loess mean estimates of time trends are shown as block solid lines along with 95% confidence intervals as gray ribbons. The date of Gamma’s first detection is indicated as the gray dotted vertical line.

**Extended Data Fig. 1:**
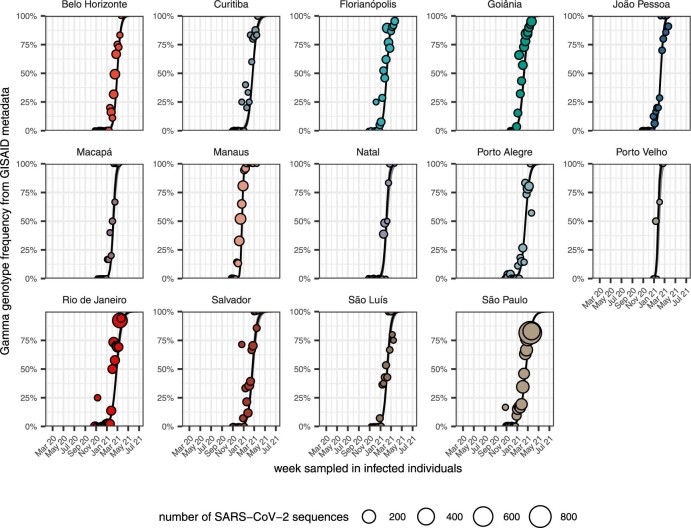
Spatiotemporal expansion of the SARS-CoV-2 Gamma variant across Brazil. SARS-CoV-2 genome sequences were obtained from the GISAID repository^23^ along with confirmed lineage assignments. The frequency of the Gamma variant (dots) in weekly SARS-CoV-2 genome sequence counts (size of dots) is shown along with posterior median estimates of Gamma’s variant frequencies (black line) under the Bayesian multi-strain fatality model and 95% credible intervals (grey ribbon).

**Extended Data Fig. 2:**
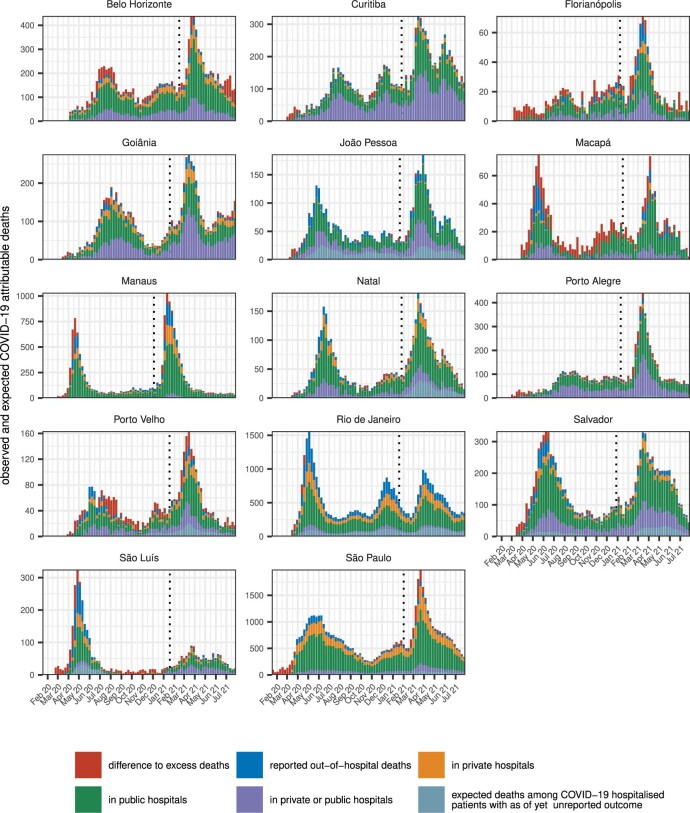
Underreporting-adjusted COVID-19 attributable deaths. Reported COVID-19 attributable deaths in the SIVEP-Gripe platform were adjusted for in-hospital underreporting, by counting a proportion of hospitalised patients with as of yet unreported outcome as fatal, and for likely out-of-hospital under-reporting, by comparison against population excess deaths derived from all-cause mortality data of the Brazilian Civil Registry (Supplementary Information). The date of Gamma’s first detection in each city is shown as a vertical dotted line.

With the aim of characterizing longitudinal trends in COVID-19-attributable fatality rates since the beginning of the pandemic, we investigated mortality in hospitalized patients admitted with polymerase chain reaction (PCR)-confirmed, clinically diagnosed or suspected SARS-CoV-2 infection (SARS-CoV-2-attributable infection)^16^ from 20 January 2020 to 26 July 2021 across Brazil. We focused on hospital settings, as these are the places where most lives are saved, and we restricted analyses to 14 of 27 Brazilian state capitals for which SARS-CoV-2 genomic data were publicly available from GISAID^23^ to characterize the expansion dynamics of Gamma and control for potentially elevated disease severity of Gamma in hospitals (Fig. 2a and Extended Data Fig. 1). In total, 641,618 patients with SARS-CoV-2-attributable infection were admitted to hospitals across the 14 cities and reported to Brazil’s Sistema de Informação da Vigilância Epidemiológica da Gripe (SIVEP-Gripe) database (Fig. 1 and Supplementary Table 1). To match these data to city-level transmission dynamics, we retained for analysis the 495,100 COVID-19-attributable hospital admissions among residents in each state capital. We further excluded 32,734 resident patients who were administered at least one vaccine dose before hospitalization to avoid confounding of time trends in fatality rates with vaccine rollout, which occurred during the same period. Thus, our study population comprised the remaining 462,366 COVID-19-attributable hospital admissions in residents (Supplementary Fig. 1). Among those, 120,288 (26.02%) outcomes were fatal. However, 32,585 (7%) admissions had unreported clinical outcomes, which occurred primarily after the detection of Gamma, and so it is important to account for potential underreporting of COVID-19-attributable deaths^16,24,25^. We estimated 10,029 additional fatalities by considering the proportion of deaths among patients with known outcomes, stratified by age and observation week (Methods, Extended Data Fig. 2 and Supplementary Table 2).

**Extended Data Fig. 3:**
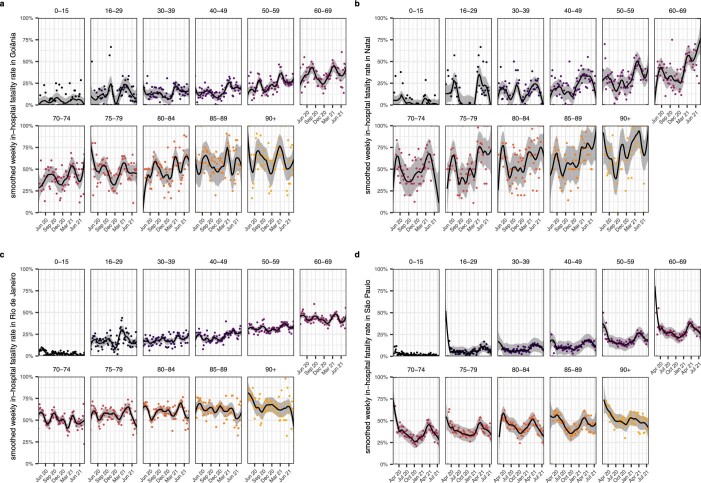
Time trends in age-specific COVID-19 in-hospital fatality rates. Weekly, age-specific COVID-19 in-hospital fatality rates are shown as dots, and non-parametric loess mean estimates of time trends are shown as block solid line along with 95% confidence intervals as grey ribbon. The date of Gamma’s first detection is indicated with vertical black lines. Data are shown for Goiânia (**a**), Natal (**b**), Rio de Janeiro (**c**), and São Paulo (**d**).

Figure 2c shows the empirical COVID-19 in-hospital fatality rates for Manaus, defined as the proportion of underreporting-adjusted deaths in weekly COVID-19-attributable hospital admissions among residents with no evidence of vaccination before admission (see Extended Data Fig. 3 for four other state capitals). The observed, age-dependent patterns in COVID-19 in-hospital fatality rates are consistent with earlier observations that COVID-19 fatality rates increase with age^26^. Moreover, we observe, across all age bands, marked increases in COVID-19 in-hospital fatality rates after detection of Gamma, a pattern that is consistent across the 14 cities. However, the within-age band variation reveals stark geographical differences and temporal fluctuations since the beginning of the pandemic rather than just since the emergence of Gamma, reinforcing previous findings on geographical heterogeneity of mortality in the first 3 months of the pandemic^17^. For example, in Belo Horizonte, no age group experienced shocks of COVID-19 in-hospital fatality rates above 50% that lasted at least four consecutive weeks, whereas, in Macapá, all age groups of 40 or older experienced such fatality shocks (Supplementary Table 3). We also found that the increases in COVID-19 in-hospital fatality rates after detection of Gamma were largely transient, declining in tandem with fewer admissions, as shown in Fig. 2c for Manaus.

**Fig. 3:**
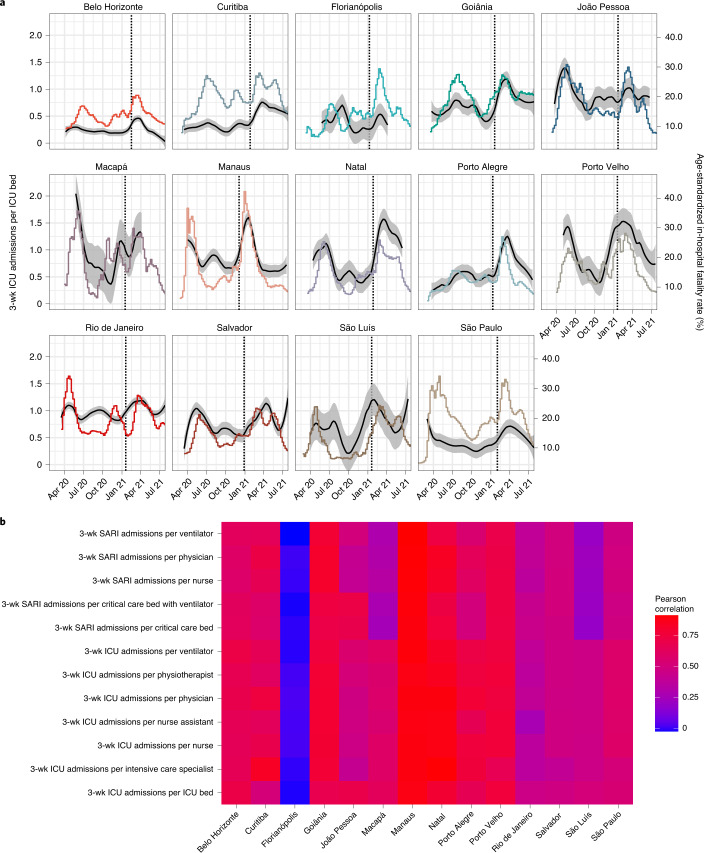
Time trends in age-standardized COVID-19 in-hospital fatality rates and pandemic healthcare pressure. **a**, Non-parametric median estimates (lines) and 95% confidence interval (ribbons) of age-standardized COVID-19 in-hospital fatality rates (black, right-hand-side axis) are shown against the healthcare pressure index of ICU admissions over 3 weeks per available ICU bed in each city (color, left-hand-side axis). The date of first detection of Gamma is added as a vertical dashed line. **b**, Heat map of Pearson correlation coefficients between age-standardized in-hospital fatality rates and each pandemic healthcare pressure index. SARI, severe acute respiratory infection; wk, week.

To obtain a simple measure on the extent of spatio-temporal variation, we first estimated smoothed, non-parametric trends through the weekly, age-specific in-hospital fatality rates (Methods). Then, and because the population age compositions vary across cities, we weighted each age-specific trend in a location by the proportion of the 14 cities’ populations in the corresponding age band, resulting in age-standardized estimates of weekly COVID-19 in-hospital fatality rates. Figure 3a shows the age-standardized COVID-19 in-hospital fatality rates since the beginning of the pandemic across locations in black lines, and Table 2 reports the minimum and maximum observed values in each location. The minimum age-standardized in-hospital fatality rates occurred either at the start of the observation period or between waves of hospital admissions in each location for all cities except Belo Horizonte, where the lowest fatality rates were seen at the end of the observation period. We performed additional analyses suggesting that this further drop in fatality rates in Belo Horizonte at the end of the observation period is likely related to missing data on vaccine status in hospitalized patients in Belo Horizonte (Supplementary Fig. 2). For this reason, and to guard our analyses against other potential confounders, such as improved treatment with dexamethasone, we evaluated the minimum fatality rates over the period until the detection of Gamma. In Belo Horizonte, the minimum age-standardized COVID-19 in-hospital fatality rate before detection of Gamma was 7.7%, and the maximum value over the entire study period was 12.7%, a 1.64-fold increase. We observed higher fold increases in all other state capitals, apart from Rio de Janeiro where age-standardized in-hospital fatality rates were very high throughout the study period. Rates tended to reach similar baseline values between shock periods in each location and were lowest in state capitals from the South and Southeast regions of Brazil. The maximum rates occurred in most locations after the first detection of Gamma, except for João Pessoa, Macapá and Rio de Janeiro where they occurred before the first detection of Gamma. Overall, rates tended to be highest in the North, Northeast and Center-West regions of Brazil.

**Table 2:** Temporal fluctuations in COVID-19-attributable in-hospital fatality rate and avoidable COVID-19-attributable deaths in hospitals

Location	Observation period	COVID-19 attributable hospital admissions in unvaccinated residents		Age-standardized weekly COVID-19 in-hospital fatality rate (HFR)			Estimated avoidable COVID-19 deaths in hospitals	
		Total	Deaths^a^	Lowest (%)^b^	Highest (%)^b^	Fold increase^b^	Assuming the lowest HFR in each city (%)^c^	Assuming the lowest HFR across all 14 cities (%)^c^
Belo Horizonte	06/04/20–26/07/21	42,113	7,692	7.7	12.7	1.64	29.1 (24.6–33.7)	29.1 (24.6–33.7)
Curitiba	02/03/20–26/07/21	33,015	7,535	8.1	18.1	2.23	39.6 (34.2–44.7)	47.3 (43.9–50.8)
Florianópolis	09/03/20–26/07/21	4,032	893	7.8	17.1	2.20	22.9 (12.5–32.6)	44.5 (40.9–48.1)
Goiânia	16/03/20–26/07/21	20,044	6,246	11.6	25.9	2.23	44.7 (39.0–50.0)	61.2 (58.6–63.7)
João Pessoa	09/03/20–26/07/21	10,552	3,636	15.3	29.7	1.94	18.8 (13.0–25.1)	65.9 (63.7–68.1)
Macapá	30/03/20–26/07/21	3,169	1,011	11.0	41.5	3.78	41.6 (30.5–51.8)	68.4 (66.2–70.6)
Manaus	24/02/20–26/07/21	26,260	10,168	16.4	33.4	2.04	39.3 (35.9–42.3)	70.9 (69–72.9)
Natal	16/03/20–26/07/21	9,344	3,512	11.4	32.9	2.87	35.4 (30.2–40.5)	66.9 (64.8–69)
Porto Alegre	02/03/20–26/07/21	16,106	5,266	8.6	27.1	3.16	41.8 (37.7–45.7)	59.6 (57.1–62.3)
Porto Velho	30/03/20–26/07/21	6,795	2,473	11.5	32.2	2.79	39.1 (32.2–45.5)	70.3 (68.3–72.4)
Rio de Janeiro	16/03/20–26/07/21	73,139	28,053	19.2	26.0	1.35	10.2 (8.5–11.9)	66.9 (64.8–69)
Salvador	16/03/20–26/07/21	26,964	8,509	9.7	26.9	2.76	20.8 (16.2–25.2)	61.3 (58.8–63.9)
São Luís	24/02/20–26/07/21	8,545	2,547	8.2	26.5	3.25	35.7 (28.4–42.4)	60.5 (57.9–63.1)
São Paulo	20/01/20–26/07/21	182,288	42,769	8.8	19.8	2.24	36.2 (33.9%-–38.4)	50.1 (46.9–53.4)
All 14 cities	20/01/20–26/07/21	462,366	130,317				29.8 (28.7–30.9)	57.1 (54.3–59.9)

^a^Observed deaths plus expected deaths in COVID-19-attributable hospital admissions with unreported outcome.

^b^Age-specific COVID-19-attributable in-hospital fatality rates were estimated from linked data on underreporting-adjusted deaths in COVID-19-attributable hospital admissions. Non-parametric loess estimates were obtained and weighted by the population age composition across cities. Lowest fatality rates were calculated in the period before Gamma’s first detection in each location, and highest fatality rates were calculated including the time after Gamma’s first detection. The lowest fatality rates in the period before Gamma’s first detection agreed with those observed across the entire study period for all cities except Belo Horizonte. See the main text for further details and analyses.

^c^Estimates are based on hypothetical scenarios evaluated under the Bayesian multi-strain fatality model, assuming the lowest observed in-hospital fatality rates seen in the periods before Gamma’s first detection in each location.

### Healthcare pressure indices track in-hospital fatality rates

Since the early phase of the COVID-19 pandemic, investments to avoid a widespread collapse of Brazil’s unified health system have resulted in increased availability of equipment such as ventilators and intensive care unit (ICU) beds as well as trained healthcare professionals—but with considerable geographic differences^19,27^. In the wider context of substantial underfunding of Brazil’s unified health system before the pandemic^14,28^ and disparities in healthcare resources across and within Brazil’s states^29^, here we introduce pandemic healthcare pressure indices that monitor in-hospital healthcare load at the city level. We obtained healthcare-facility-level microdata on personnel (nurses, nurse assistants, physiotherapists, physicians and intensive care specialists) and equipment (critical care beds, ICU beds and ventilators) from Brazil’s National Register of Health Facilities (Cadastro Nacional de Estabelecimentos de Saúde (CNES)), which we consolidated into monthly time series for each location (Methods).

**Extended Data Fig. 4:**
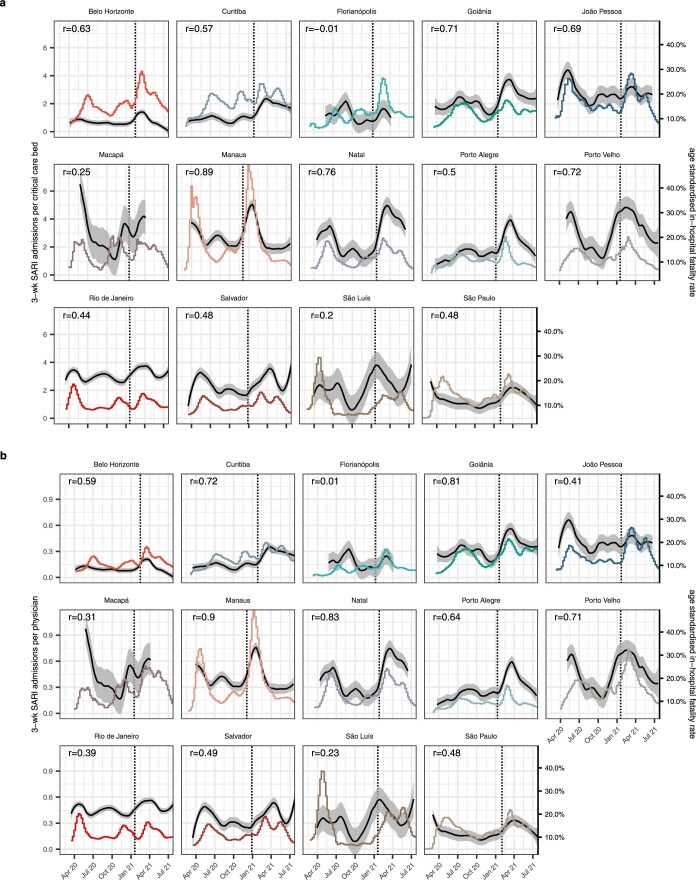
Time evolution of pandemic healthcare pressure indices, part 1. SARI admissions in this and the following two weeks per hospital resource are shown in colour, with y-axis on the left. In (**a**), demand per critical care bed is shown, and in (**b**) demand per physician. Non-parametric mean estimates of age-standardised COVID-19 in-hospital fatality rates are shown in black, with 95% confidence intervals as grey ribbons, and y-axis on the right. Pearson correlation coefficients (*r*) are shown in the upper left corner, and dates of Gamma’s first detection as vertical black lines.

**Extended Data Fig. 5:**
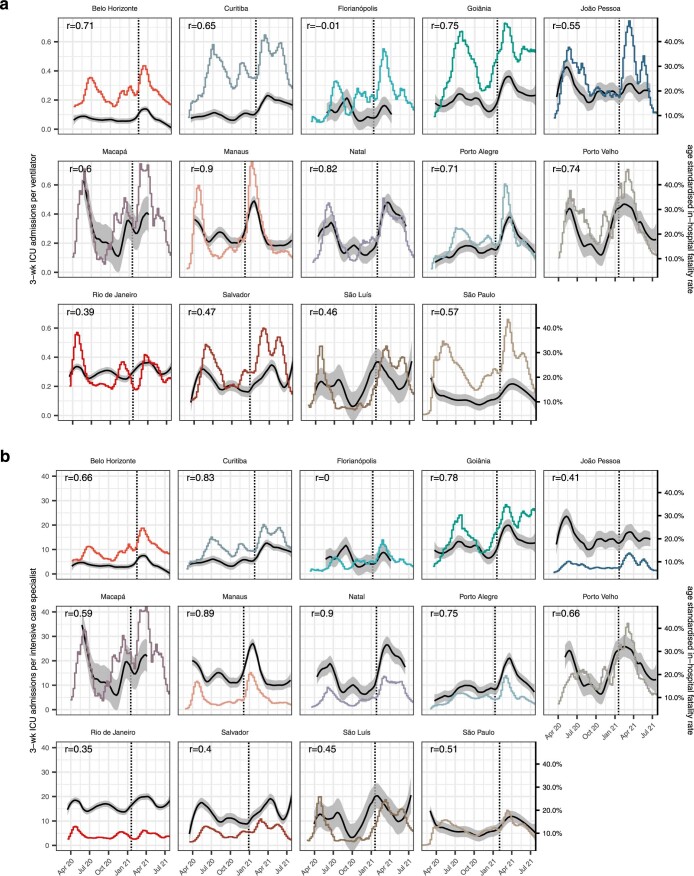
Time evolution of pandemic healthcare pressure indices, part 2. ICU admissions in this and the following two weeks per hospital resource are shown in colour, with y-axis on the left. In (**a**), demand per ventilator is shown, and in (**b**) demand per intensive care specialist. Non-parametric mean estimates of age-standardised COVID-19 in-hospital fatality rates are shown in black, with 95% confidence intervals as grey ribbons, and y-axis on the right. Pearson correlation coefficients (*r*) are shown in the upper left corner, and dates of Gamma’s first detection as vertical lines.

We found large inequities in healthcare resources across Brazil. In March 2020 the number of available ventilators per 100,000 population ranged from 21.7 in Macapá to 102 in Porto Alegre, and the number of physicians per 100,000 population ranged from 124 in Macapá to 631 in Belo Horizonte (Supplementary Table 4). From these data, we constructed healthcare pressure indices that capture changes in hospital demand per available resource, with demand comprising all hospitalized patients with severe acute respiratory infection, including non-residents and individuals with vaccine breakthrough infections (Fig. 1 and Methods). One such index, the moving sum of ICU admissions over 3 weeks per ICU bed, is shown in Fig. 3a, and four further indices are shown in Extended Data Figs. 4 and 5. We found that all healthcare pressure indices are strongly correlated with the age-standardized, weekly COVID-19 in-hospital fatality rates in most cities, except Florianópolis (Fig. 3b).

### The effects of severity, location and pandemic healthcare pressure

We next developed a Bayesian multi-strain fatality model to disentangle the effects of location-specific inequities, pandemic healthcare pressures and Gamma-specific disease severity on fluctuating COVID-19 in-hospital fatality rates while accounting for the substantial cumulated loss of life^30^ and age-prioritized vaccine rollout^10,21,22^. In brief, the model reconstructs the replacement dynamics of Gamma in each of the 14 state capitals from weekly SARS-CoV-2 sequence metadata and estimates the weekly number of patients who are hospitalized, respectively, with Gamma and non-Gamma SARS-CoV-2 infection in 11 age strata. We modeled age-specific in-hospital fatality rates for each variant through a regression equation that captures non-parametric location effects, fixed non-negative effects associated with the healthcare pressure indices and non-parametric virus variant effects associated with the replacement dynamics of Gamma (Methods). In the model, the non-parametric location effects do not vary in time and account for a variety of constant social, economic or healthcare-related factors that differentiate one city from another in the weeks when their observed fatality rates were lowest, such as the prevalence of comorbidities that might modify disease severity or pre-existing inequities in both the quality and the quantity of available healthcare resources^15,31^. By contrast, the healthcare pressure indices vary in time, and the strength of their association with the fatality rates is estimated through the regression equation. Patients estimated to have been infected with the Gamma variant are in the model considered separately from those estimated to have been infected with non-Gamma variants of SARS-CoV-2, which allowed us to control for Gamma-specific expansion and in-hospital disease severity when inferring associations between fatality rates and healthcare pressure.

**Extended Data Fig. 6:**
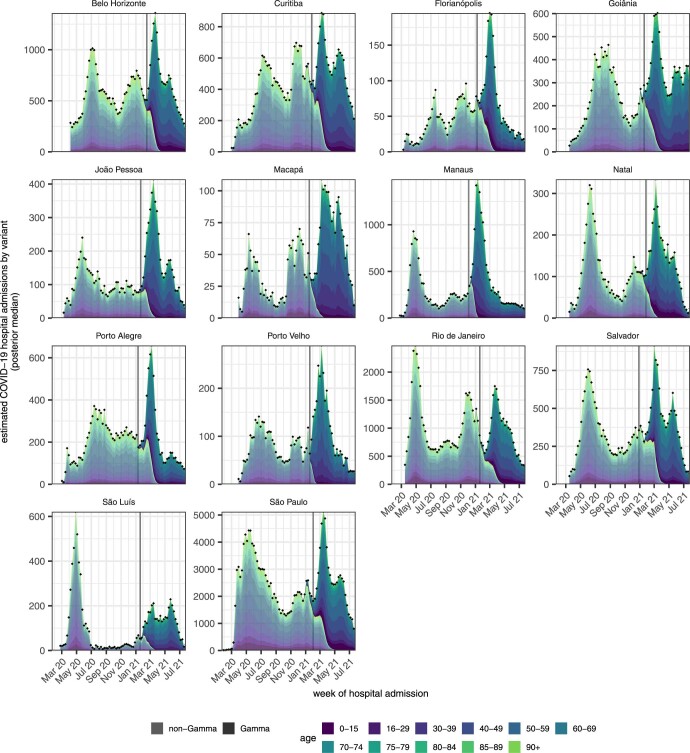
Estimated COVID-19 attributable hospital admissions by SARS-CoV-2 variant. Posterior median estimates of hospital admissions among residents in each location, that are attributed to non-Gamma variants are shown for each age band (color) in lighter shades, while those for the Gamma variant are shown in darker shades. Estimates are derived using the Bayesian multi-strain fatality model. Locations are shown across facets. The date of Gamma’s detection is shown as a grey vertical line. Observed weekly hospital admissions in residents are shown as black dots.

**Extended Data Fig. 7:**
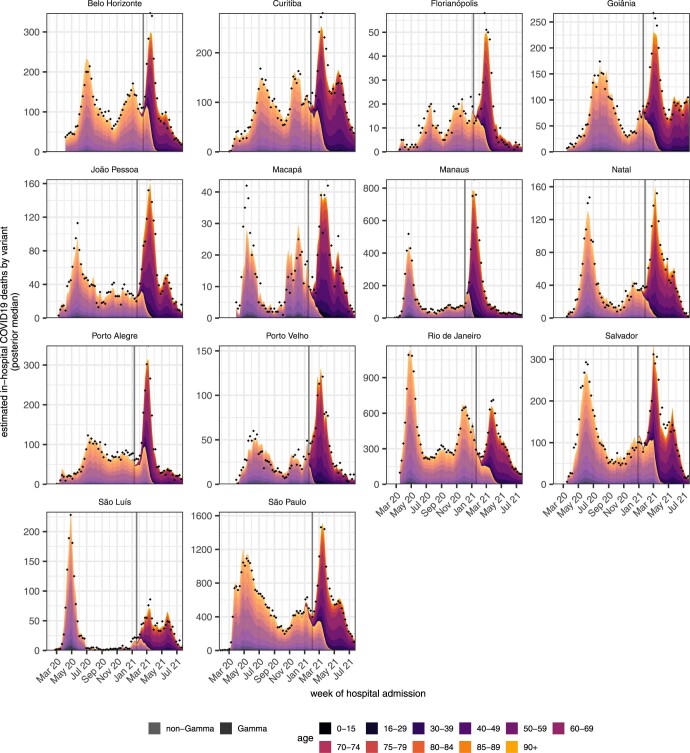
Estimated COVID-19 attributable deaths in hospitals by SARS-CoV-2 variant. Posterior median estimates of deaths following hospital admissions of residents in each location, that are attributed to non-Gamma variants are shown for each age band (color) in lighter shades, while those for the Gamma variant are shown in darker shades. Estimates are derived using the Bayesian multi-strain fatality model. Locations are shown across facets. The date of Gamma’s detection is shown as a grey vertical line. Observed weekly deaths following hospital admissions in residents are shown as black dots.

**Extended Data Fig. 8:**
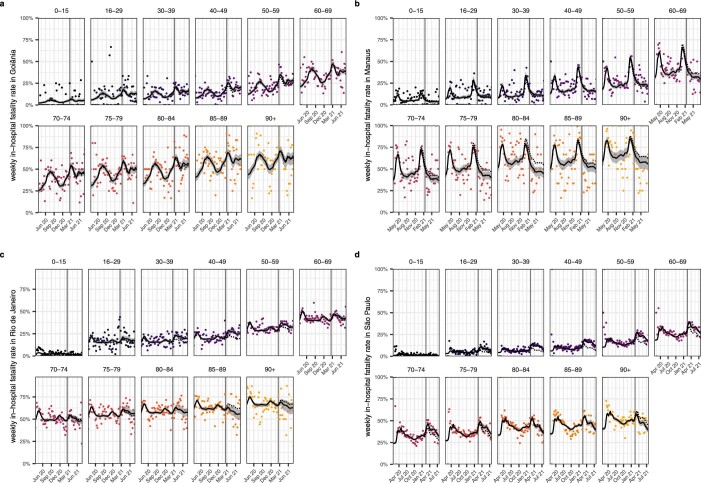
Model fits to age-specific COVID-19 in-hospital fatality rates. Weekly, age-specific in-hospital fatality rates are shown as dots. Posterior median estimates of the expected in-hospital fatality rates across variants from the Bayesian multi-strain fatality model, Equation. (8), are shown on the y-axis (black line) along with 95% credible intervals (grey ribbon). The expected in-hospital fatality rates of non-Gamma variants, Equation (6a), are shown as dotted line. The date of Gamma’s first detection is indicated as a vertical line. Data are shown for Goiânia (a), Manaus (b), Rio de Janeiro (c), and São Paulo (d).

**Extended Data Fig. 9:**
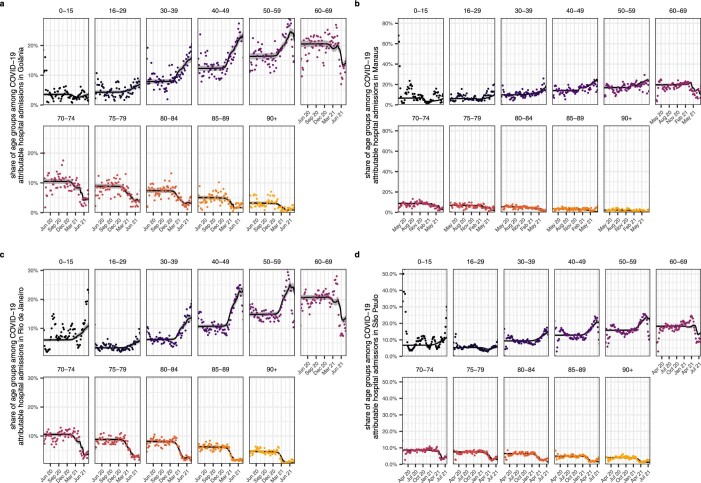
Model fits of the expected age composition of COVID-19 attributable hospital admissions. Posterior median estimates of the expected age composition of hospital admissions in each location, age band, and week, obtained with the Bayesian multi-strain fatality model are shown on the y-axis as a black line along with 95% credible intervals. Time trends are shown by week of hospital admission (x-axis). The empirical proportions are shown as dots. Data are shown for Goiânia (**a**), Manaus (**b**), Rio de Janeiro (**c**), and São Paulo (**d**).

Brazil’s rapid vaccination rollout overlapped with the temporal expansion of Gamma as well as changes in Brazil’s age-specific population structure^30^. To account for this, we used all-cause death records from Brazil’s Civil Registry and vaccine administration records from the Brazilian Ministry of Health to adjust downwards the population at risk of hospitalization per location and age group in the model (Methods). We observed substantial variation in the timing of vaccine rollout, time to second dose and vaccine type administered (Supplementary Figs. 3–7). Accounting for this variation was important to obtain good model fit to age-specific shifts in hospital admissions, deaths in hospitalized patients, in-hospital fatality rates and variant frequencies of Gamma (Extended Data Figs. 1 and 6–9).

### Regional and healthcare inequities drive in-hospital fatality rates

**Fig. 4:**
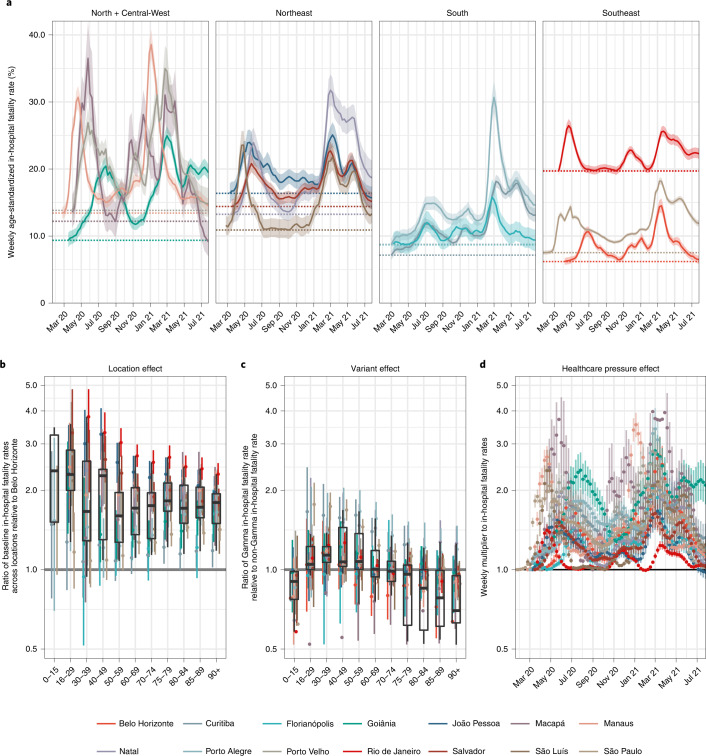
Estimated contribution of location effects, infection severity of Gamma and pandemic healthcare pressure on COVID-19 in-hospital fatality rates. **a**, Estimated weekly age-standardized COVID-19 in-hospital fatality rates, averaged across SARS-CoV-2 variants. Posterior median estimates (line) are shown with 95% CrIs (ribbon) and the lowest estimated fatality rates before detection of Gamma in each state capital (dotted horizontal line). **b**, Estimated ratio in lowest in-hospital fatality rates in each location relative to that seen in Belo Horizonte. **c**, Estimated ratio in in-hospital fatality rates for Gamma versus non-Gamma lineages of SARS-CoV-2. **d**, Estimated multiplier to the lowest age-standardized fatality rates before Gamma’s detection shown in **a**, which is associated with the pandemic healthcare pressure indices. In each plot, posterior median estimates are shown as dots and 95% CrIs as linerange. Box plots summarize posterior medians across locations (*n* = 14): the middle line is the median; the box limits represent the upper and lower quartiles; and the whiskers extend to the extreme values that are no further than 1.5 times the interquartile range. Multipliers and ratios in **b**–**d** are reported on a logarithmic scale. Posterior estimates with CrI width larger than 3 were removed for clarity of presentation.

Figure 4a compares the fitted age-standardized COVID-19 in-hospital fatality rates across Brazil’s macroregions—the North, Northeast, Central-West, Southeast and South—revealing considerable geographical heterogeneity. Before the first detection of Gamma in each location, the fitted age-standardized in-hospital fatality rate was lowest in Belo Horizonte and highest in Rio de Janeiro (shown as dotted horizontal lines in Fig. 4a). The high estimates for Rio de Janeiro prompted us to compare excess deaths derived from Brazilian’s Civil Registry to the COVID-19-attributable in-hospital deaths, which suggested that a smaller than expected proportion of hospitalized patients with unreported clinical outcomes may have died. However, Rio de Janeiro’s age-standardized COVID-19 fatality rates remained the highest even when we assume that all patients with unreported clinical outcomes were successfully treated and survived COVID-19 (Methods and Supplementary Fig. 8).

Figure 4b–d compares the estimated location and the Gamma and healthcare pressure contributions to in-hospital fatality rates (Methods). We found that the marked increase in COVID-19 in-hospital fatality rates is better explained by changes in healthcare pressures rather than a direct effect of Gamma on fatality rates in hospitalized patients. This result is inferred from three observations. First, the empirical in-hospital fatality rates also fluctuated before the detection of Gamma; second, the rates largely decreased after the initial shock after Gamma detection; and third, the rates were strongly associated with the time-varying pandemic healthcare pressure indices (Extended Data Fig. 8). Relative to Belo Horizonte, the lowest fatality rates were an estimated 1.95-fold (95% credible interval (CrI): 1.11–3.25) higher in all other state capitals, a location effect that was consistent across all age groups. Although we do not explore the exact drivers of this location effect, it is likely shaped by a combination of socioeconomic factors specific to each city, in conjunction with city-specific, pre-existing inequities in the availability, quality and accessibility of healthcare^32,33^. At peak times, pandemic healthcare pressures as measured by our indices were associated with a 2.33-fold (95% CrI: 1.39–4.27) multiplicative effect across the 14 cities, and this contribution was stronger in places that already had high in-hospital fatality rates at baseline. Gamma was not associated with a statistically significant effect on in-hospital fatality rates after controlling for healthcare pressures (posterior *P* = 0.14 that Gamma-specific in-hospital disease severity is lower than or equal to that of non-Gamma variants in patients aged 30–39 years compared to non-Gamma variants of SARS-CoV-2 and larger in those younger than 30 years and older than 39 years).

### Avoidable deaths in the absence of resource limitations

To quantify the effect that the observed fluctuations in COVID-19-attributable in-hospital rates had on the death toll in the 14 state capitals, we considered counterfactual simulations where infection waves and resource limitations did not result in surging in-hospital fatality rates (Methods). We consider this counterfactual scenario conservative as it assumes, for each location, achievable in-hospital fatality rates that are based on the lowest values observed before the first detection of Gamma in each state capital and, thus, before subsequent improvements in clinical management, patient triage and treatments (for example, dexamethasone). Across the 14 state capitals, we found that, in the absence of pandemic resource limitations, deaths could have been reduced by an estimated 29.8% (95% CrI: 28.7–30.9%) (Table 2).

Using additional counterfactual simulations, we further evaluated how many in-hospital deaths could have been avoided in the absence of regional inequities and pandemic resource limitations—that is, if all 14 state capitals would have experienced throughout the pandemic the lowest observed in-hospital fatality rates across all 14 cities, which were found in Belo Horizonte. We consider these estimations less conservative, because the observed, low in-hospital fatality rates in Belo Horizonte could reflect a large range of factors that might not be translatable to other locations in Brazil. We found that in-hospital deaths could have been reduced by an estimated 57.1% (95% CrI: 54.3–59.9%) in the 14 state capitals (Table 2).

## Discussion

This study highlights extensive geographic and temporal variation in COVID-19 in-hospital fatality rates since the beginning of the epidemic in 14 state capitals across Brazil. Our analyses indicate that the observed variation is driven primarily by shortages in healthcare capacity, which, in turn, emerged from a combination of pre-pandemic regional inequities and increased healthcare pressure brought about by epidemic waves of SARS-CoV-2 transmission. The extent and degree of this mismatch between supply (of staff and equipment) and demand (patients requiring hospitalization for COVID-19) is highly dynamic, varying considerably over the course of the epidemic with the magnitude of each infection wave.

Our findings should be considered in the context of the following limitations. First, Brazil’s inpatient records are limited in that comorbidity factors, vaccination status or SARS-CoV-2 variant information are either frequently missing or not available at the individual level^34^. This makes it challenging to comprehensively control for individual-level factors that can modulate fatality rates^35,36^. However, Brazil’s freely accessible inpatient data constitute one of the world’s largest databases to characterize the pandemic effect of COVID-19 in a middle-income country and across areas that differ substantially in baseline development and available healthcare resources. In sensitivity analyses, we considered SARS-CoV-2 sequence data from alternative sources as well as alternative patient inclusion criteria, which suggest that our estimates of location and healthcare pressure effect sizes are robust (Supplementary Figs. 9–14).

Second, here we delineate fluctuations in COVID-19 in-hospital fatality rates at city level to focus on the extensive spatio-temporal heterogeneity in fatality rates across Brazil. We recognize that this broader geographic focus masks important differences within cities, and, thus, we cannot identify the exact factors determining the substantial location effects that we measure. However, these are likely related to (1) differences in catchment populations, as vulnerable populations with poor healthcare access are highly clustered across Brazil’s largest cities^29,37^; (2) underfunding of the public healthcare system^14,28^ and emerging discrepancies in healthcare resources compared to private hospitals^17,32,37,38^; or (3) inequities in the quality and capabilities of healthcare systems that are well-documented—for example, for pre-pandemic sepsis survival rates^39^. Additional relevant factors could include (4) differences in demand reflecting variation in epidemic magnitude^32^ and (5) the timing and extent of public health measures aimed at controlling spread and preventing infection in vulnerable groups^19,27,33^. As such, the location effects could also reflect healthcare pressures that are present already at the lowest incidences of COVID-19 hospital admissions in each location. We observed substantial fluctuations in in-hospital fatality rates even in private hospitals of São Paulo city (Supplementary Fig. 15), suggesting that large effects of healthcare pressure on in-hospital fatality rates are common across Brazil.

Third, the pandemic healthcare pressure indices that we derived are based on data reported to CNES, which does not capture all resource limitations, such as the acute shortages in oxygen supply that were experienced in January 2021 in Manaus^40^, and is prone to potential reporting biases^20^. In our view, the inferred associations between healthcare pressure indices and fatality rates demonstrate that, where resources are limited, real-time monitoring of available resources is especially important to identify critical resource limitations and avoid the lamentable shocks in death rates that we describe for many state capitals across Brazil.

Fourth, in characterizing hospital fatality rates in a broad Brazilian context, we are ignoring historical policy aspects that can be a source of heterogeneity. The standard of healthcare per unit staff member can be improved by federal coordinated actions and policies on training as well as unambiguous governmental medical policies to prevent disinformation^41^. These federal policies also extend to financing to ensure that the underlying structure of primary care and emergency services all present a standardized quality of care^42^.

Finally, our analyses start with hospitalization, which is a limitation because in-hospital fatality rates also depend on which, and under what circumstances, severely ill patients are admitted to hospitals. Thus, it is possible that healthcare demand was amplified due to an increased risk of hospitalization after infection with a variant of concern with higher infection severity, as has been shown for SARS-CoV-2 Alpha^35,43^. We also found evidence that, in several locations, out-of-hospital deaths surged at times of peak demand (Extended Data Fig. 2), and that, in hospitalized patients with a fatal outcome, the time to death after admission tended to be shorter during peak demand (Supplementary Fig. 16). These observations indicate that increased healthcare pressure acts to shape in-hospital fatality rates through distinct mechanisms, likely through a combination of both the reduced ability to provide adequate care and an increase in the average severity of admitted patients (with only the most severely ill admitted during periods of highest pressure). In this context, if less severe COVID-19 cases could also have been admitted, we expect that the estimated proportion of avoidable COVID-19 in-hospital deaths in the absence of healthcare pressure effects would be lower than what is reported here. At the same time, the projected numbers are likely an underestimate of COVID-19 deaths that could have been avoided in the absence of healthcare pressure effects because we did not account for deaths in severely ill individuals who were not cared for in hospitals.

It can be challenging to generalize the effects of healthcare pressure indices to other countries and in different temporal waves of the pandemic. The concept of health system resilience is well-studied^44–46^. However, our results highlight that established indices such as the Global Health Security Index^47^ are inadequate to reliably measure healthcare resilience. Similar results to what we have presented in this study have been found in Greece, Israel and the United States^48–50^. The factors affecting in-hospital fatality rates in Brazil are universal and highlight that healthcare resilience cannot be simply solved by responding to short-term shocks or attempting to dynamically redistribute capacity. Instead, it requires an investment in long-term measures, such as training healthcare workers, strengthening public health functions and funding in excess of current need^51^.

The implications of the inferred scale of location inequities and healthcare shortages in Brazil are substantial. As of 26 July 2021, we estimate that approximately one-quarter of the COVID-19-attributable deaths in the hospitals in the 14 cities studied could have been avoided if healthcare pressure had not exacerbated baseline fatality rates. Taking the percent reduction across the 14 state capitals as indicative and generalizable to all of Brazil, we estimate that, as of 26 July 2021, 176,399 (169,888–182,911) of Brazil’s observed 591,945 COVID-19-attributable deaths in hospitals could have been avoided in the absence of pandemic resource limitations. We also estimate that approximately half of the COVID-19-attributable deaths in the hospitals in the 14 cities studied could have been avoided if, in addition, all hospitals would have had the same baseline COVID-19 fatality rates as those observed in Belo Horizonte. Extrapolating Belo Horizonte’s lowest observed in-hospital fatality rates to all of Brazil, we estimate that, as of 26 July 2021, 337,763 (321,485–354,575) of Brazil’s COVID-19-attributable in-hospital deaths could have been avoided. Our findings are particularly important in calibrating the risk posed by new SARS-CoV-2 variants of concern. We find that the effect of Gamma in Brazil’s hospitals has predominantly been indirect and mediated through pre-existing geographic inequities, transient infection waves and concomitant shocks in healthcare demand. In conclusion, our results suggest that investments in healthcare resources, healthcare optimization and pandemic preparedness are critical to minimize population-wide mortality and morbidity caused by highly transmissible and deadly pathogens such as SARS-CoV-2, especially in low- and middle-income countries.

## Methods

To characterize the role of pre-pandemic geographic inequities and increased healthcare pressure brought about by epidemic waves of SARS-CoV-2 transmission, we took a systematic approach that involved longitudinal statistical analyses of hospitalized patients with suspected or confirmed COVID-19 infection in 14 state capitals of 27 Brazilian federal states, longitudinal analyses of healthcare resources in each city, mathematical modellng to disentangle the effect of geographic inequities and healthcare pressure from Gamma’s rapid expansion across Brazil and validation against external data. The following sections summarize our methods. All data used are publicly available, and no further ethics consent was required. Code and data are fully available (see ‘Data availability’ section).

### Data to characterize in-hospital fatality rates

We obtained publicly available individual records of patients with severe acute respiratory infection in public or private hospitals across Brazil that were reported in the SIVEP-Gripe database, release 31 January 2022 (https://opendatasus.saude.gov.br/dataset/srag-2020, https://opendatasus.saude.gov.br/dataset/srag-2021-e-2022).

Individual patient records in Brazil’s SIVEP-Gripe database do not contain linked SARS-CoV-2 sequence data. To characterize the associations and effect sizes of geographic inequities and healthcare pressure on COVID-19 in-hospital fatality rates while controlling for variant-specific effects of Gamma on in-hospital severity, we focused analyses on geographically well-defined locations where SARS-CoV-2 sequence metadata were independently and freely available. We searched GISAID (https://www.gisaid.org) on 14 June 2021 for sequence data associated with SARS-CoV-2 Gamma virus genome sequences in the 27 federal units (26 Brazilian states and the Federal District). Gamma genome data were available for 16 federal units: Amapá, Amazonas, Bahia, Goiás, Maranhão, Mato Grosso do Sul, Minas Gerais, Paraíba, Paraná, Rio de Janeiro, Rio Grande do Norte, Rio Grande do Sul, Rondônia, Santa Catarina, São Paulo and Tocantins. We focused our analysis on the state capitals in these federal units because key variables, such as population size, vaccination coverage and healthcare indicators, were more directly available at the level of state capitals. We assumed that the frequency of Gamma in state capitals is similar to the frequency of Gamma across that state, measured by available sequence metadata. Two state capitals, Palmas and Campo Grande, were excluded from further analysis due to limited, weekly age-specific COVID-19 hospital admission and death counts of residents reported to the SIVEP-Gripe database. The cities in our sampling frame were, thus, Belo Horizonte, Curitiba, Florianópolis, Goiânia, João Pessoa, Macapá, Manaus, Natal, Porto Alegre, Porto Velho, Rio de Janeiro, Salvador, São Luís and São Paulo. Reflecting the geographical expansion of COVID-19 through Brazil over time, our observation periods varied across cities. The start dates were defined as the Monday after the date on which at least 2.5 patients with suspected or confirmed COVID-19 per 100,000 population were hospitalized in each location. The end date was set to 26 July 2021 (Supplementary Table 1). Records were filtered to exclude out-of-hospital deaths, defined as individuals with a missing hospital admission date or who died on the day of admission; to exclude confirmed infections with other respiratory pathogens or with missing diagnosis; to exclude non-residents to match city-level population denominators; and to exclude patients with at least one vaccine dose before hospital admission to that ensure that trends in fatality rates were not confounded with vaccine status. To avoid bias^16^, our data, thus, included patients with PCR-confirmed, clinically diagnosed or suspected COVID-19 infection (Supplementary Fig. 17). Alternative inclusion criteria were considered in sensitivity analyses and are reported below. The data are available at inst/data/SIVEP_hospital_31-01-2022-all.rds in the GitHub repository.

Population size estimates in each city were retrieved by sex and 1-year age bands from the 2020 National Household Sample Survey COVID-19, Pesquisa Nacional por Amostra de Domicílios COVID-19 (ref. ^52^). The projections were reconciled with available vaccination records of residents in each city, so that, at most, 99% of residents received at least one vaccine dose during the observation period (Supplementary Fig. 18). The data are available at inst/data/PNADc_vaxadj_ population_210802.csv in the GitHub repository.

To further investigate the shocks in in-hospital fatality rates before and after Gamma’s emergence, we obtained monthly data on healthcare resources reported by healthcare facilities to the National Register of Health Facilities (CNES)^53^, release 15 September 2021. Data on healthcare resources are mandatory to report by both public and private healthcare facilities and comprised personnel and equipment. Nurses (CBO 2235), nurse assistants (CBO 3222), physiotherapists (CBO 2236), physicians (CBO 2231, 2251, 2252 and 2253) and intensive care physicians (CBO 225150) were summed per month and location according to the 2002 Brazilian Classification of Occupations (Classificação Brasileira de Ocupações (CBO)), and, where possible, records were validated by name and professional health card number. ICU beds summed per month and location reported ICU type II, ICU type III and COVID type II beds. ICU type I beds (code 74) refer to an older standard that is being phased out since 2017 and were not counted. Type II ICU beds (code 75) represent the minimum requirement for severe cases of COVID-19 requiring ventilation. Type III ICU beds (code 76) are, by regulation, reserved to ICU patients with multiple acute failures of vital organs, or to patients at risk of developing them, with an immediate threat to life^54^. Since March 2020, new ICU beds were created to try to minimize the immediate risk of healthcare system collapse. These ICU beds were intended exclusively for treatment of COVID-19 and are designated COVID-19 type II ICU beds (code 51). At least one microprocessor-controlled lung ventilator must be available for every two ICU beds. Considering that both a ventilator and an ICU bed are necessary for adequate treatment of severe COVID-19, we counted only adult ICU beds with a matched ventilator per month and location, defined as the minimum number of available ventilators (respirador or ventilador) and the number of adult ICU beds in the same healthcare facility that are reported to CNES, which we then aggregated across healthcare facilities in the same location^29^. Critical care beds summed ICU beds and intermediate care beds (code 95), and we considered counts with and without controlling for available reported ventilators. To guard against potential reporting differences or bias in reported bed types^20^, we also considered monthly counts of available ventilators as resource. The number of reported ventilators (respirador or ventilador, code 64) does not include ventilators already held for ICU or critical care beds. Thus, for each healthcare facility, we counted the number of lung ventilators reported to CNES and added one ventilator for every two reported ICU type II, type III or COVID-19 type II beds, added one ventilator for every three reported intermediate beds and then aggregated across healthcare facilities in the same location. The data are available at inst/data/IPEA_ICUbeds_physicians_210928.csv in the GitHub repository.

### Statistical analysis of in-hospital fatality rates

To characterize longitudinal trends to in-hospital fatality rates, we considered the age strata

1 $$\begin{array}{lll}{{{{{\mathcal{A}}}}}} &=& \left\{ {0{\mbox{--}}15, 16{\mbox{--}}29, 30{\mbox{--}}39, 40{\mbox{--}}49, 50{\mbox{--}}59, 60{\mbox{--}}69,}\right.\\&& \quad \left. {70{\mbox{--}}74, 75{\mbox{--}}79, 80{\mbox{--}}84, 85{\mbox{--}}89, 90+ } \right\},\end{array}$$

to control for age dependence in fatality rates. To capture temporal shocks in fatality rates, all data were aggregated to weeks. In COVID-19-attributable patients with unreported outcomes, deaths were predicted independently in each location, week and age strata based on the fatality rates in COVID-19-attributable patients with observed outcomes in the two previous weeks, assuming data were missing at random over the 3 weeks $w - 1,\,w - 2,w$ in each age group and each location. Empirical fatality rates *z*_*l*,*a*,*w*_ were defined as the ratio of underreporting-adjusted fatalities in unvaccinated, resident, COVID-19-attributable hospital admissions in location *l*, age strata *a* and week *w* except for weeks with no such hospital admissions. Longitudinal trends ${\hat{z}}_{l,a,w}$ were obtained by fitting the non-parametric loess smoother as implemented in the R stats package version 4.0.3 with argument span = 0.3 to the empirical fatality rates *z*_*l*,*a*,*w*_, separately for each location and age band, and starting from the first week with non-zero hospital admissions in each age band. To compare fatality rates across cities in a simple statistic while accounting for the substantial differences in age demographics (Supplementary Fig. 18), we calculated age-standardized, weekly in-hospital fatality rates with

2 $${\hat{z}}_{l,w} = \mathop {\sum }\limits_a \frac{{n_a^{{{{{{\mathrm{cities}}}}}}}}}{{\mathop {\sum }\nolimits_b n_b^{{{{{{\mathrm{cities}}}}}}}}}{\hat{z}}_{l,a,w},$$

where ${\hat{z}}_{l,a,w}$ are the smoothed fatality rates in location *l*, age strata *a* and week *w*, and $n_a^{{{{{{\mathrm{cities}}}}}}}$ are our 2020 projected population sizes in age band *a* across the 14 cities (see above). To avoid extrapolation, the smoothed rates (2) were generated onward from the first week for which the ${\hat{z}}_{l,a,w}$ were defined for all age groups.

We next defined 12 pandemic healthcare pressure indices that quantified hospital demand over time in each location. Demand was defined in terms of all hospital admissions for severe acute respiratory infections (that is, including non-residents, cases caused by other pathogens and vaccinated individuals) and all ICU admissions among patients hospitalized for severe acute respiratory infection. Records were again filtered to exclude out-of-hospital deaths as defined above. The healthcare pressure indices were calculated by considering demand per available healthcare resource over time. To account for the fact that admitted patients remain in care for several weeks, we considered rolling sums of the form

3 $$x_{l,w}^{{{{{3 {\mbox{-}} {{{\mathrm{wk}}}}}}}}\,{{{{{\mathrm{ICU}}}}}}\,{{{{{\mathrm{adm}}}}}}\,{{{{{\mathrm{per}}}}}}\,{{{{{\mathrm{ICU}}}}}}\,{{{{{\mathrm{bed}}}}}}} = \left( {\mathop {\sum }\limits_{i = 0}^3 h_{l,w + i}^{{{{{{\mathrm{ICU}}}}}}}} \right){\big /}r_{l,w}^{{{{{{\mathrm{ICU}}}}}}\,{{{{{\mathrm{beds}}}}}}},$$

where $h_{l,w}^{{{{{{\mathrm{ICU}}}}}}}$ are the number of ICU admissions, and $r_{l,w}^{{{{{{\mathrm{ICU}}}}}}\,{{{{{\mathrm{beds}}}}}}}$ are the number of ICU beds in location *l* and week *w*. Data on resources were available per month. In weeks overlapping months, we used weighted averages of the resources in the corresponding months to define the weekly resources, and, otherwise, we used the monthly values. Supplementary Table 5 provides definitions for all healthcare pressure indices considered. Associations between the healthcare pressure indices and smoothed, age-standardized in-hospital fatality rates were quantified with Pearson correlation coefficients.

### Data to account for Gamma’s expansion dynamics

To assess fluctuating in-hospital fatality rates in the context of the spatio-temporal expansion of the SARS-CoV-2 Gamma variant, we obtained from GISAID^23^ publicly available viral genome sequences across Brazilian states with collection date from 1 November 2020 to 31 March 2021 on 28 June 2021. Acknowledgment tables with GISAID IDs are available in the acknowledgments_GISAID_Tables directory in the GitHub repository. Records with incomplete collection dates were removed, de-duplicated and classified as Gamma or non-Gamma variants with pangolin version 3.0.6, Pangolearn version 1.2.12, scorpio lineage (https://github.com/cov-lineages/pangolin). In total, we retained sequences and metadata from 7,221 samples, most of which were from São Paulo (1,104 viral genomes), with an average of 158 genomes per state. Gamma and non-Gamma frequencies were calculated for each week and location, assuming that the frequency of Gamma in state capitals was similar to the state-level frequency of Gamma in the GISAID data. The data are available at inst/data/genomic_data_210702.csv in the GitHub repository and shown in Supplementary Fig. 19. Supplementary Table 1 reports the weeks in which Gamma was first detected in each location, and Extended Data Fig. 1 shows the proportion of Gamma sequences over time in each location. Throughout, we denote the week index in which Gamma was in each location first detected by $W_l^{{{{{{\mathrm{detect}}}}}} {\mbox{-}} \Gamma }$, the number of sequenced genotypes in location *l* and collection week *w* by *s*_*l*,*w*_ and the number sequenced genotypes attributed to the Gamma variant with pangolin by $s_{l,w}^\Gamma$.

### Dating Gamma’s emergence

To guide our modeling, we dated the emergence of the SARS-CoV-2 Gamma variant in each location using viral phylogenetic methods based on a subset of 2,212 high-coverage SARS-CoV-2 Gamma genome sequences across the 14 locations under study. This dataset included five sequences that had been recovered from the International Guarulhos Airport in São Paulo. The reference strain WH04 (GISAID EPI_ISL_406801) was appended to the sequence dataset before multiple sequence alignment using MAFFT version 7 (ref. ^55^). After removing untranscribed terminal regions, the resulting multiple sequence alignment had a length of 29,409 nucleotides. Maximum likelihood phylogenetic trees were estimated using IQTree version 2 (ref. ^56^) under the Jukes Cantor (JC69) substitution model^57^. We next used TempEst version 1.5.3 (ref. ^58^) to regress root-to-tip distances against sampling dates and identify data quality and data annotation problems before further phylogenetic analysis. Specifically, we discarded virus genomes characterized by a genetic distance to WH04 of more than 4 standard deviations from the epi-week mean genetic distance to WH04 (ref. ^59^). A total of ten sequences were excluded from subsequent phylogenetic analysis. The GISAID identifiers of the excluded sequences were EPI_ISL_1821206, EPI_ISL_2249444, EPI_ISL_1821208 (earliest available sequence from São Paulo, dated 2020-11-03), EPI_ISL_1821217, EPI_ISL_2249440 (earliest available sequence from Rio de Janeiro, dated 2020-11-18), EPI_ISL_2249443, EPI_ISL_2241496 (earliest available sequence from Paraíba, dated 2020-10-01) and EPI_ISL_1715135, EPI_ISL_ 1821257 and EPI_ISL_2249437.

Estimating time trees for large alignments can be computationally intractable. Thus, we follow a computation strategy similar to du Plessis et al.^59^ and Gutierrez et al.^60^ that involves (1) estimating an evolutionary rate using a subsample of the genome dataset of interest and (2) using a simpler computational approach to estimate time trees for the complete genome dataset. For step (1), we randomly selected a maximum of 20 sequences per state (except for Paraíba and Rondônia, which had only 15 and nine sequences available, respectively, during the study period). This generated a dataset of 264 genome sequences. Sequences with earliest and latest dates of collection from each state were kept in the alignment to increase temporal signal of the resulting dataset. We used BEAST version 1.10 (ref. ^61^) to estimate an evolutionary rate under a Hasegawa–Kishino–Yano^62^ substitution model and a strict molecular clock with a continuous-time Markov chain prior. We used a Bayesian skygrid with ten grid points as a demographic tree prior^63^. The BEAST.xml file is available at inst/utils/BEAST_thorney_P1.xml in the GitHub repository. Four Markov chain Monte Carlo (MCMC) chains were run for 50 million steps, sampling parameters and trees every 50,000 steps. Convergence of the MCMC chains was assessed using Tracer version 1.7 (ref. ^64^). For step (2), the complete dataset was analyzed using BEAST version 1.10.5 (ref. ^65^) using a newly developed approach that significantly reduces computation time. This approach takes in a rooted phylogenetic maximum likelihood tree (instead of an alignment) and rescales its branches into time units. The likelihood of each branch length is modeled as a Poisson distribution with a mean that is directly proportional to the clock rate^66,67^. We used a rate of 4.864 × 10^−4^ substitutions/site/year based on the median clock rate estimate obtained from step (1). We defined a coalescent skygrid prior and used the best-fitting IQTree maximum likelihood tree rooted in TempEst as a starting data tree. Two independent MCMC chains were run for 1,000 million MCMC steps and combined after discarding 10% of the run as burn-in to generate an empirical posterior tree distribution. Convergence was assessed using Tracer version 1.7 (ref. ^64^). We next used a 14-state asymmetric discrete Bayesian molecular clock phylogeographic approach^68^ implemented in BEAST version 1.10.4 (ref. ^61^) to infer ancestral state locations on an empirical distribution of 500 posterior time trees. For sequences with known travel history, we assigned the state of infection instead of state of reporting. We estimated unknown state locations for the sequences collected at the International Guarulhos Airport in São Paulo. We tracked the complete jump history of viral movement events between each pair of states^69–71^. We used a recently developed tool, the TreeMarkovJumpHistoryAnalyzer, which collects Markov jumps and their timings from a posterior tree distribution with Markov jump histories^72^, available at inst/utils/P.1_MJumps_complete_history.xml in the GitHub repository. To date the most common recent ancestor of the earliest local transmission cluster in each state, we used a customised R script to summarize the posterior probability distribution densities for the earliest time of the introduction leading to two or more descendants in each of the 14 states. The phylogenetically estimated dates of emergence of Gamma in each city are shown in Supplementary Table 1. Throughout, we denote the week of the posterior median estimate with $W_l^{{{{{{\mathrm{emerge}}}}}} {\mbox{-}} \Gamma }$.

### Modeling to disentangle factors associated with shocks in in-hospital fatality rates

We developed a Bayesian multi-strain fatality model to disentangle the effects of pre-pandemic geographic inequities, pandemic healthcare pressures and Gamma’s in-hospital disease severity on fluctuating COVID-19 in-hospital fatality rates. This section describes the modeling framework, and full details are given in the final section below.

Overall, the model is structured in three components. In the first model component, a logistic function is fitted to the Gamma variant frequency data to estimate the proportion of Gamma infections in location *l* and week *w*, which, throughout, we denote by *α*_*l*,*w*_. We here use the phylogenetically derived emergence times of the Gamma variant in each city and specify that *α*_*l*,*w*_ is essentially zero for all weeks before $W_l^{{{{{{\mathrm{emerge}}}}}} {\mbox{-}} \Gamma }$. In the second model component, the proportion of hospitalized patients with Gamma and non-Gamma infections in the weekly hospital admissions are estimated by city, age and week of admission. The model-based attribution of hospital admissions to Gamma and non-Gamma variants is based on the estimated expansion of the Gamma variant in each city through the *α*_*l*,*w*_. In the third model component, we describe the fatality rates in the Gamma-attributable hospital admissions and non-Gamma-attributable hospital admissions through two corresponding regression equations. The regression equations comprise non-parametric location effects, fixed effects associated with the healthcare pressure indices and non-parametric virus variant effects of Gamma-specific disease severity in hospitals. The free parameters of the model, and, in particular, the parameters in the regression equations, can be well-identified from the weekly Gamma variant frequency data (denoted by $s_{l,w}^\Gamma$), the weekly COVID-19-attributable hospital admissions (denoted by $h_{l,a,w}^{{{{{{\mathrm{res}}}}}}}$) and the number of deaths occurring in the linked, individual-level records of weekly age-specific hospital admissions during follow-up (denoted by $h_{l,a,w}^{{{{{{{{\mathrm{res}}}} {\mbox{-}} {{{\mathrm{adj}}}} {\mbox{-}} {{{\mathrm{D}}}}}}}}}$) (Extended Data Figs. 1 and 6–9). Recall that the deaths count the observed deaths in patients with known outcome and the expected deaths in patients with unreported outcome, indexed by week *w* of hospital admission. In the rest of this section, we provide more detail on the second and third component of the model.

In the second model component, the proportion of hospitalized patients with Gamma infection is estimated by city, age and week of admission. To build intuition, we considered modeling the expected values of the hospital admissions $h_{l,a,w}^{{{{{{\mathrm{res}}}}}}}$ with

4 $${\Bbb E}\left( {h_{l,a,w}^{{{{{{\mathrm{res}}}}}}}} \right) = \left( {\alpha _{l,w}\pi _{l,a}^\Gamma + \left( {1 - \alpha _{l,w}} \right)\pi _{l,a}^{{{{{{{{\mathrm{non}}}} {\mbox{-}} }}}}\Gamma }} \right)h_{l,w}^{{{{{{\mathrm{res}}}}}}},$$

where $h_{l,w}^{{{{{{\mathrm{res}}}}}}}$ is the sum of hospital admissions across age strata; $\pi _{l,a}^{{{{{{\mathrm{non}}}}}}{\mbox{-}}\Gamma }$ is the characteristic age composition of non-Gamma hospital admissions in location *l* that is independent of time; and $\pi _{l,a}^\Gamma$ is the characteristic age composition of Gamma hospital admissions. It is clear that $\pi _{l,a}^{{{{{{\mathrm{non}}}}}} {\mbox{-}} \Gamma }$ can be inferred from patient records when *α*_*l*,*w*_ ≈ 0—that is, from before Gamma’s expansion—and $\pi _{l,a}^\Gamma$ can be inferred from patient records when *α*_*l*,*w*_ ≈ 1—that is, from after Gamma’s expansion. Across time, the proportion of hospital admissions in age band *a* is described as a mixture of the Gamma and non-Gamma proportions that is determined by the increasing weights of Gamma’s variant frequency *α*_*l*,*w*_ and the decreasing weights of the non-Gamma variant frequencies 1 − *α*_*l*,*w*_. The model encodes robustness in the sense that good model fit can be obtained only when the observed age distributions of hospitalized patients shift over time according to Gamma’s expansion. Following (4), in a particular age stratum *a* and week *w*, the expected number of hospital admissions attributable to Gamma is described by

5 $$\frac{{\alpha _{l,w}\pi _{l,a}^\Gamma }}{{\alpha _{l,w}\pi _{l,a}^\Gamma + \left( {1 - \alpha _{l,w}} \right)\pi _{l,a}^{{{{{{\mathrm{non}}}}}}{\mbox{-}}\Gamma }}}h_{l,w}^{{{{{{\mathrm{res}}}}}}}.$$

Equations (4) and (5) are extended in the full model to account for the fact that the age distribution of hospitalized patients is also changing with higher cumulative mortality in older age groups and with prioritized vaccination of older age groups (see below).

In the third model component, we quantify and model the fatality rates in the Gamma-attributable hospital admissions (recall (5)) and non-Gamma-attributable hospital admissions (analogous to (5)) separately. We model in-hospital fatality rates in week *w*, location *l* and age group *a* that are respectively infected with non-Gamma and Gamma variants with the decomposition

6a $$\zeta _{l,a,w}^{{{{{{\mathrm{non}}}}}}{\mbox{-}}\Gamma } = {{{{{\mathrm{logit}}}}}}^{ - 1}\left( {\eta _{l,a}^{{{{{{\mathrm{non}}}}}}{\mbox{-}}\Gamma } + X_{l,w}\beta _l} \right)$$

6b $$\zeta _{l,a,w}^\Gamma = {{{{{\mathrm{logit}}}}}}^{ - 1}\left( {\eta _{l,a}^{{{{{{\mathrm{non}}}}}}{\mbox{-}}\Gamma } + \eta _{l,a}^{\Gamma {\mbox{-}}{{{{{\mathrm{random}}}}{\mbox{-}}{{{\mathrm{effect}}}}}}} + X_{l,w}\beta _l} \right),$$

where $X_{l,w} \in {\Bbb R}^{1 \times p}$ are *p* standardized healthcare pressure indices in location *l* and week *w* and $\beta _l \in {\Bbb R}^p$ are constrained to be non-negative. For each location, we standardized the healthcare pressure indices to zero for the week in which the empirical fatality rates (2) are lowest before Gamma’s detection, which was typically at the beginning of the pandemic and observation period. Specifically, we standardized each index according to

7 $${\tilde{x}}_{l,p,w} = \frac{{x_{l,p,w} - x_{l,p,{\tilde{w}}_l}}}{{{{{{{\mathrm{s.d.}}}}}}\left( {x_{l,p,w}} \right)}},$$

where ${\tilde{w}}_l = {{{{{\mathrm{argmin}}}}}}_{w = 1:\left( {W_l^{{{{{{\mathrm{detect}}}}}} {\mbox{-}} \Gamma } - 1} \right)} {\hat{z}}_{l,w}$ and s.d. denotes the standard deviation of the indicator across all weeks in location *l*, using the notation in (2) and (3). The *p* regression coefficients *β*_*l*_ were constrained to be positive so that each standardized index of increasing hospital demand pressure could only have an increasing effect on fatality rates when *β*_*l*_ > 0, or no effect when *β*_*l*_ = 0, and not offset each other and artifactually increase the effect of another index.

Thus, we can interpret the ${{{{{\mathrm{logit}}}}}}^{ - 1}\left( {\eta _{l,a}^{{{{{{\mathrm{non}}}}}}{\mbox{-}}\Gamma }} \right)$ as the lowest observed fatality rate in each location and age group (technically defined before the emergence of Gamma to guard against potential confounders in Belo Horizonte as described in the main text, and except for Belo Horizonte equivalent to over the entire study period) and take ratios of these terms across locations to quantify the effect of geographic inequities (location effect). These ratios measure non-specific baseline differences in fatality rates across the state capitals that are not captured in our healthcare pressure indices. Next, for each location, we can interpret the ratio of ${{{{{\mathrm{logit}}}}}}^{ - 1}\left( {\eta _{l,a}^{{{{{{\mathrm{non}}}}}}{\mbox{-}}\Gamma } + X_{l,w}\beta _l} \right)$ divided by ${{{{{\mathrm{logit}}}}}}^{ - 1}\left( {\eta _{l,a}^{{{{{{\mathrm{non}}}}}}{\mbox{-}}\Gamma }} \right)$ as the multiplier to in-hospital fatality rates in week *w* and age group *a* that is associated with the increasing healthcare pressure indices (healthcare pressure effect). In (6b), $\eta _{l,a}^{\Gamma {\mbox{-}}{{{{{\mathrm{random}}}}{\mbox{-}}{{{\mathrm{effect}}}}}}}$ are non-parametric random effects that measure age-specific deviations in fatality rates in hospitalized patients attributed to Gamma infection. So, for each location, we can interpret the ratio of ${{{{{\mathrm{logit}}}}}}^{ - 1}\left( {\eta _{l,a}^{{{{{{\mathrm{non}}}}}}{\mbox{-}}\Gamma } + \eta _{l,a}^{\Gamma {\mbox{-}}{{{{{\mathrm{random}}}}{\mbox{-}}{{{\mathrm{effect}}}}}}}} \right)$ divided by ${{{{{\mathrm{logit}}}}}}^{ - 1}\left( {\eta _{l,a}^{{{{{{\mathrm{non}}}}}}{\mbox{-}}\Gamma }} \right)$ as the multiplier to fatality rates in hospitalized patients that is attributable to Gamma infection versus non-Gamma infection (variant effect in hospitals), controlling for differences in healthcare pressure in the weeks before and after the emergence of Gamma (because the *X*_*l,w*_*β*_*l*_ are zeroed) as well as cumulative mortality and vaccination in the full model (see below). We note that the regression model is measuring associations and is not guaranteed to identify causal relationships.

It follows again from (4) that, in a particular age group *a* and week *w*, the in-hospital fatality rate in Gamma and non-Gamma patients is described by

8 $$\begin{array}{l}\zeta _{l,a,w} = \frac{{\left( {1 - \alpha _{l,w}} \right)\pi _{l,a}^{{{{{{\mathrm{non}}}}}}{\mbox{-}}\Gamma }}}{{\alpha _{l,w}\pi _{l,a}^\Gamma + \left( {1 - \alpha _{l,w}} \right)\pi _{l,a}^{{{{{{\mathrm{non}}}}}}{\mbox{-}}\Gamma }}}\zeta _{l,a,w}^{{{{{{\mathrm{non}}}}}}{\mbox{-}}\Gamma } + \frac{{\alpha _{l,w}\pi _{l,a}^\Gamma }}{{\alpha _{l,w}\pi _{l,a}^\Gamma + \left( {1 - \alpha _{l,w}} \right)\pi _{l,a}^{{{{{{\mathrm{non}}}}}}{\mbox{-}}\Gamma }}}\zeta _{l,a,w}^\Gamma .\end{array}$$

Equation (8) enables us to estimate, for each location, the unknowns in (6) from the longitudinal records of age-specific deaths in the denominator of hospitalized patients with COVID-19 in location *l*, age group *a* and week *w* and expanding variant frequency of Gamma in weekly SARS-CoV-2 sequence data.

### Model validation and sensitivity analyses

Concerns on SARS-CoV-2 sequence sample representativeness^73^ prompted us to re-evaluate our findings using monthly state-level variant frequency data reported by Rede Genômica FioCruz (http://wwwgenomahcov.fiocruz.br/dashboard/). Data on monthly Gamma variant frequencies in each Brazilian federal unit or state between November 2020 and May 2021 were retrieved from the dashboard on 15 June 2021. We again assumed that the variant frequencies reported at state level are representative of the variant frequencies in state capitals. We also considered weekly city-level Gamma variant frequency data from Manaus, Belo Horizonte and São Paulo obtained under controlled sampling frames. In Manaus, samples from PCR-positive residents testing in two private laboratories through nasal and oropharyngeal swabs were selected at random regardless of cycle threshold values for sequencing. The samples were sequenced and processed using the ARTIC bioinformatics pipeline^74^ as described in Faria et al.^3^. Viral genomes recovered from 147 samples collected between 1 November 2020 and 10 January 2021 had sufficient genome coverage enabling lineage classification with pangolin version 2.2.1 (refs. ^75,76^). In Belo Horizonte, samples were selected at random from PCR-positive residents in three laboratories (Laboratório Hermes Pardini, Laboratório de Biologia Integrativa, UFMG, and Laboratório Municipal de Referência, PBH). The samples were sequenced on the Illumina MiSeq platform and processed using a custom pipeline. Identified mutations were manually inspected, and the sequences were classified using pangolin version 2.2.1 (ref. ^75^). In total, 27 samples were classified as Gamma and 47 samples to other lineages. For São Paulo, sequences were generated by the Adolf Lutz Institute, a national public health and reference laboratory for São Paulo State, Brazil, retrieved from GISAID (https://www.gisaid.org) for the period 1 November 2020 to 31 March 2021. In total, 76 sequences were analyzed between 1 November 2020 and 31 March 2021. The data are shown in Supplementary Table 6. We found only minor differences in our primary findings depending on what SARS-CoV-2 variant frequency data were used (Supplementary Figs. 9–14).

Given long-term underfunding of Brazil’s public healthcare system, we investigated if geographic inequities could be modulated by different proportions of patients in public and private hospitals and if the strong fluctuations in COVID-19 in-hospital fatality rates are also observed in private hospitals. For São Paulo, we could classify most hospitals as either private or public. We found consistently lower in-hospital fatality rates in private hospitals that, however, fluctuated in synchrony with those in public hospitals, in line with our primary findings (Supplementary Fig. 15).

In Belo Horizonte, the age-standardized COVID-19 in-hospital fatality rates declined over the summer months of 2021 to levels well below those seen during earlier time periods (Fig. 4a). We hypothesized that larger numbers of patients hospitalized in Belo Horizonte may have been vaccinated, with no vaccination record reported to SIVEP-Gripe. To address this possibility, we performed a sub-analysis in which we excluded patients with unreported vaccination status in the SIVEP-Gripe dataset (Supplementary Fig. 2). In the figure, fatality rate estimates are not shown for weeks in which patient denominators were too small. In this sub-analysis, we found larger discrepancies in fatality rates compared to the main analysis only for Belo Horizonte, suggesting that missing data on vaccination status have likely no substantial effect on our overall findings. However, for Belo Horizonte, it is possible that our in-hospital fatality rate estimates are confounded with unreported vaccination status.

The highest age-standardized COVID-19 in-hospital fatality rates were observed in Rio de Janeiro, against the national trend of declining rates in Brazil’s South and Southeast macroregions. It is possible that smaller proportions of hospitalized patients with unreported clinical outcomes may have died, and analyses were repeated assuming that all patients with unreported clinical outcomes survived. We found, in comparison to other cities, that Rio de Janeiro’s baseline in-hospital fatality rates were, in this sensitivity analysis, considerably lower than in the main analysis but remained overall largest and showed the same strong fluctuations as in the main analysis (Supplementary Fig. 8).

In-hospital fatality rates also depend on which, and under what circumstances, severely ill patients are admitted to hospitals. This prompted us to investigate if the observed fluctuations in COVID-19 fatality rates could, in part, be the result of concomitant changes in the profile of admitted patients. There are limited data on disease severity at time of hospital admission available in SIVEP-Gripe; however, one indicator that can be readily calculated is the time between hospital admission and death in patients with a fatal outcome (Supplementary Fig. 16). In Macapá, Manaus, Porto Alegre and Porto Velho, we found substantially shorter times to death when the number of COVID-19-attributable hospital admissions peaked, suggesting that admitted patients may already have been at a more severe clinical stage when admitted or that, during times of peak demand, healthcare pressure in hospitals both increased fatality rates and led to faster progression to death. Further data on out-of-hospital deaths shows that COVID-19-attributable out-of-hospital deaths typically occurred during times of peak demand, with the exception of Rio de Janeiro (Extended Data Fig. 2). Together, these data suggest that increased healthcare pressure likely acts to shape in-hospital fatality rates through distinct mechanisms, through a combination of both a reduced ability to provide adequate care and an increase in the average severity of admitted patients.

### Full statistical model, generated quantities and counterfactuals

We begin by describing the three components of the full statistical model, which were already motivated and introduced above. The first model component describes the temporal expansion of Gamma in hospital admissions through a logistic function and is given by

9a $$s_{l,w}^\Gamma \sim {{{{{\mathrm{Beta}}}}{\mbox{-}}{{{\mathrm{Binomial}}}}}}\left( {s_{l,w},\frac{{\alpha _{l,w}}}{{\theta _1}},\frac{{1 - \alpha _{l,w}}}{{\theta _1}}} \right),$$

9b $$\alpha _{l,w} = \frac{1}{{1 + {{{{{\mathrm{exp}}}}}}\left( { - \alpha _l^{{{{{{\mathrm{growth}}}}}}}\left( {w - \alpha _l^{{{{{{\mathrm{mid}}}}}}}} \right)} \right)}}$$

9c $$\alpha _l^{{{{{{\mathrm{growth}}}}}}} \sim {{{{{\mathrm{Normal}}}}}}\left( {0,0.2^2} \right)$$

9d $$\alpha _l^{{{{{{\mathrm{mid}}}}}}} \sim {{{{{\mathrm{Normal}}}}}}\left( {\alpha _l^{{{{{{\mathrm{mid}}}}{\mbox{-}}{{{\mathrm{mean}}}}}}},3^2} \right)$$

9e $$\alpha _{l,1:\left( {W_l^{{{{{{\mathrm{emerge}}}}}}{\mbox{-}}\Gamma } - 1} \right)} \sim {{{{{\mathrm{Normal}}}}{\mbox{-}}{{{\mathrm{ccdf}}}}}}\left( {0,0.0025^2} \right)$$

9f $$\theta _1 \sim {{{{{\mathrm{Exponential}}}}}}\left( {20} \right),$$

where the Beta-Binomial is specified in terms of the shape–shape parameterization with mean *s*_*l,w*_*α*_*l,w*_ and variance equal to the binomial variance component $s_{l,w}\alpha _{l,w}\left( {1 - \alpha _{l,w}} \right)$ multiplied by $\left( {1 + \left( {s_{l,w} - 1} \right)\frac{1}{{\theta _1^{ - 1} + 1}}} \right)$ to allow for overdispersion in the variant frequency data. Time runs in units of weeks from the start of the first wave until the end of the observation period in each location. The prior mean for the midpoint of the logistic function, $\alpha _l^{{{{{{\mathrm{mid}}}}{\mbox{-}}{{{\mathrm{mean}}}}}}}$, was set to the week in which the ratio $s_{l,w}^\Gamma /s_{l,w}$ was closest to 0.5 in location *l*. We force the variant frequencies of Gamma to close to zero before the week of Gamma’s emergence in each location, which is implemented through the informative prior (9e), where normal-ccdf denotes the survival function of a normal density. The prior in (9f) peaks at zero and, thus, favors the least complex model with no overdispersion.

In the second model component, we couple (9b) to decompose the COVID-19-attributable hospital admissions $h_{l,a,w}^{{{{{{\mathrm{res}}}}}}}$ by admissions with Gamma and non-Gamma variant. We expand on equation (4) to account for demographic changes in the population at risk of severe infection, either through higher cumulative mortality in older ages or through prioritized vaccination of older ages, and denote the population that remains at risk of severe infection in location *l*, age group *a* and week *w* by $n_{l,a,w}^R$. Then, we assumed characteristic per-capita rates $\lambda _{l,a}^\Gamma$, $\lambda _{l,a}^{{{{{{\mathrm{non}}}}}}{\mbox{-}}\Gamma }$ of severe infection and hospitalization with Gamma and non-Gamma variants, respectively, and considered modeling the expected values of the hospital admissions $h_{l,a,w}^{{{{{{\mathrm{res}}}}}}}$ with

10a $${\Bbb E}\left( {h_{l,a,w}^{{{{{{\mathrm{res}}}}}}}} \right) = \left( {\alpha _{l,w}\pi _{l,a,w}^\Gamma + \left( {1 - \alpha _{l,w}} \right)\pi _{l,a,w}^{{{{{{\mathrm{non}}}}}}{\mbox{-}}\Gamma }} \right)h_{l,w}^{{{{{{\mathrm{res}}}}}}},$$

10b $$\pi _{l,a,w}^\Gamma = \lambda _{l,a}^\Gamma n_{l,a,w}^R{\big /}\mathop {\sum }\limits_a \lambda _{l,a}^\Gamma n_{l,a,w}^R,$$

10c $$\pi _{l,a,w}^{{{{{{\mathrm{non}}}}}}{\mbox{-}}\Gamma } = \lambda _{l,a}^{{{{{{\mathrm{non}}}}}}{\mbox{-}}\Gamma }n_{l,a,w}^R{\big /}\mathop {\sum }\limits_a \lambda _{l,a}^{{{{{{\mathrm{non}}}}}} {\mbox{-}} \Gamma }n_{l,a,w}^R,$$

and used $\pi _{l,a,w}^{{{{{{\mathrm{non}}}}}}{\mbox{-}}\Gamma }$, $\pi _{l,a,w}^\Gamma$ in lieu of $\pi _{l,a}^{{{{{{\mathrm{non}}}}}}{\mbox{-}}\Gamma }$, $\pi _{l,a}^\Gamma$ in equations (4), (5) and (8). We specified $n_{l,a,w}^R$ as follows. First, to account for cumulative mortality, we calculated excess deaths based on the all-cause deaths reported by Brazil’s Civil Registry (https://transparencia.registrocivil.org.br/registros) and subtracted from the population size projections the larger of cumulated excess deaths or all reported COVID-19 deaths in the SIVEP-Gripe database (Supplementary Fig. 6). Second, to account for increasing vaccine protection, we calculated vaccination coverage by vaccine administered in each location, age group and week (Supplementary Figs. 3–5), based on vaccine administration records from the Brazilian Ministry of Health (https://opendatasus.saude.gov.br/dataset/covid-19-vacinacao). We then calculated the population at risk of severe infection, $n_{l,a,w}^R$, by subtracting further a proportion of the individuals vaccinated with one or two doses for at least 2 weeks according to the vaccine-specific efficacy values^77–81^ listed in Supplementary Table 7. Following the mean structure of (10), the second model component is given by

11a $${{{{{\bf{h}}}}}}_{l,w}^{{{{{{\mathrm{res}}}}}}} \sim {{{{{\mathrm{Dirichlet}}}}{\mbox{-}}{{{\mathrm{Multinomial}}}}}}\left( {h_{l,w}^{{{{{{\mathrm{res}}}}{\mbox{-}}{{{\mathrm{sum}}}}}}},{\bf \phi} _{l,w}{{{{{\bf{\uppi }}}}}}_{l,w}} \right)$$

11b $$\pi _{l,a,w} = \alpha _{l,w}\pi _{l,a,w}^\Gamma + \left( {1 - \alpha _{l,w}} \right)\pi _{l,a,w}^{{{{{{\mathrm{non}}}}}}{\mbox{-}}\Gamma }$$

11c $$\pi _{l,a,w}^\Gamma = {{{{{\mathrm{softmax}}}}}}\left( {{{{{{\mathrm{log}}}}}}\,\lambda _{l,a}^\Gamma + {{{{{\mathrm{log}}}}}}\,n_{l,a,w}^R} \right)$$

11d $$\pi _{l,a,w}^{{{{{{\mathrm{non}}}}}}{\mbox{-}}\Gamma } = {{{{{\mathrm{softmax}}}}}}\left( {{{{{{\mathrm{log}}}}}}\, \lambda _{l,a}^{{{{{{\mathrm{non}}}}}}{\mbox{-}}\Gamma } + {{{{{\mathrm{log}}}}}}\, n_{l,a,w}^R} \right)$$

11e $${{{{{\mathrm{log}}}}}}\,\lambda _{l,a}^\Gamma \sim {{{{{\mathrm{N}}}}}}\left( {0,1} \right)$$

11f $${{{{{\mathrm{log}}}}}}\,\lambda _{l,a}^{{{{{{\mathrm{non}}}}}}{\mbox{-}}\Gamma } \sim {{{{{\mathrm{N}}}}}}\left( {0,1} \right)$$

11g $$\phi _{l,w} = \left( {h_{l,w}^{{{{{{\mathrm{res}}}}{\mbox{-}}{{{\mathrm{sum}}}}}}} - 1} \right)/\left( {\theta _2 + 1} \right)$$

11h $$\theta _2 \sim {{{{{\mathrm{Exponential}}}}}}\left( {20} \right),$$

where we denote the vector of age-specific hospital admissions in location *l* and week *w* by ${{{{{\mathbf{h}}}}}}_{l,w}^{{{{{{\mathrm{res}}}}}}} = \left( {h_{l,a,w}^{{{{{{\mathrm{res}}}}}}}} \right)_{a \in {{{{{\mathcal{A}}}}}}}$ and the vector of the age composition of hospital admissions in location *l* and week *w* by ${{{{{\mathbf{\pi }}}}}}_{l,w} = \left( {\pi _{l,a,w}} \right)_{a \in {{{{{\mathcal{A}}}}}}}$ again such that $\mathop {\sum }\limits_a \pi _{l,a,w} = 1$. The Dirichlet-Multinomial is in standard sample size-scale parameterization such that the means are given by $h_{l,w}^{{{{{{\mathrm{res}}}}{\mbox{-}}{{{\mathrm{sum}}}}}}}\pi _{l,a,w}$ as in (9b). The softmax transformations (11c) and (11d) allow for convenient prior specifications of the log hospital admission rates on the real line and run over age bands *a* for fixed location *l* and fixed week *w*. The scale parameter *ϕ*_*l,w*_ is conditional on the known $h_{l,w}^{{{{{{\mathrm{res}}}}{\mbox{-}}{{{\mathrm{sum}}}}}}}$ re-parameterized into the overdispersion parameter *θ*_2_, with *θ*_2_>0, and the prior on *θ*_2_ in (11h) peaks at zero and favors the least complex model with no overdispersion.

In the third model component, we describe the expected fatality rates in the COVID-19-attributable hospital admissions according to the modeled Gamma and non-Gamma fatality rates (6) and their corresponding contributions to the overall fatality rates according to Gamma’s increasing frequency (8), which is given by

12a $$h_{l,a,w}^{{{{{{\mathrm{res}}}}{\mbox{-}}{{{\mathrm{adj}}}}{\mbox{-}}{{{\mathrm{D}}}}}}} \sim {{{{{\mathrm{Beta}}}}{\mbox{-}}{{{\mathrm{Binomial}}}}}}\left( {h_{l,a,w}^{{{{{{\mathrm{res}}}}}}},\zeta _{l,a,w}/\theta _3,\left( {1 - \zeta _{l,a,w}} \right)/\theta _3} \right)$$

12b $$\zeta _{l,a,w} = \frac{{\left( {1 - \alpha _{l,w}} \right)\pi _{l,a,w}^{{{{{{\mathrm{non}}}}}}{\mbox{-}}\Gamma }}}{{\alpha _{l,w}\pi _{l,a,w}^\Gamma + \left( {1 - \alpha _{l,w}} \right)\pi _{l,a,w}^{{{{{{\mathrm{non}}}}}}{\mbox{-}}\Gamma }}}\zeta _{l,a,w}^{{{{{{\mathrm{non}}}}}}{\mbox{-}}\Gamma } + \frac{{\alpha _{l,w}\pi _{l,a,w}^\Gamma }}{{\alpha _{l,w}\pi _{l,a,w}^\Gamma + \left( {1 - \alpha _{l,w}} \right)\pi _{l,a,w}^{{{{{{\mathrm{non}}}}}}{\mbox{-}}\Gamma }}}\zeta _{l,a,w}^\Gamma$$

12c $${{{{{\mathrm{logit}}}}}}\,\zeta _{l,a,w}^{{{{{{\mathrm{non}}}}}}{\mbox{-}}\Gamma } = \eta _{l,a}^{{{{{{\mathrm{non}}}}}}{\mbox{-}}\Gamma } + X_{l,w}\beta _l$$

12d $${{{{{\mathrm{logit}}}}}}\,\zeta _{l,a,w}^\Gamma = \eta _{l,a}^{{{{{{\mathrm{non}}}}}}{\mbox{-}}\Gamma } + \eta _{l,a}^{\Gamma {\mbox{-}}{{{{{\mathrm{random}}}}{\mbox{-}}{{{\mathrm{effect}}}}}}} + X_{l,w}\beta _l$$

12e $$\eta _{l,a}^{{{{{{\mathrm{non}}}}}}{\mbox{-}}\Gamma } \sim {{{{{\mathrm{Normal}}}}}}\left( { - 0.25,1.5^2} \right)$$

12f $$\eta _{l,a}^{\Gamma {\mbox{-}}{{{{{\mathrm{random}}}}{\mbox{-}}{{{\mathrm{effect}}}}}}} \sim {{{{{\mathrm{Normal}}}}}}\left( {0,\sigma _\zeta ^2} \right)$$

12g $$\beta _{l,i} \sim {{{{{\mathrm{Normal}}}}}}_{\left[ {0,\infty } \right]}\left( {0,\kappa _{l,i}^2\tau ^2} \right)$$

12h $$\kappa _{l,i} \sim {{{{{\mathrm{Half}}}}{\mbox{-}}{{{\mathrm{Cauchy}}}}}}\left( {0,1} \right)$$

12i $$\tau \sim {{{{{\mathrm{Half}}}}{\mbox{-}}{{{\mathrm{Cauchy}}}}}}\left( {0,0.01} \right)$$

12j $$\sigma _\zeta \sim {{{{{\mathrm{Exponential}}}}}}\left( 2 \right)$$

12k $$\theta _3 \sim {{{{{\mathrm{Exponential}}}}}}\left( {100} \right),$$

where *w* = 1, …, *W*_*l*_. Importantly, we note that the deaths $h_{l,a,w}^{{{{{{\mathrm{res}}}}{\mbox{-}}{{{\mathrm{adj}}}}{\mbox{-}}{{{\mathrm{D}}}}}}}$ in (12a) are derived from the individual-level line list of hospital admissions in week *w*, adjusted only for underreporting, and so $h_{l,a,w}^{{{{{{\mathrm{res}}}}{\mbox{-}}{{{\mathrm{adj}}}}{\mbox{-}}{{{\mathrm{D}}}}}}}$ counts the deaths in exactly the $h_{l,a,w}^{{{{{{\mathrm{res}}}}}}}$ individual patients who were admitted in week *w*. The Beta-Binomial is in the shape–shape parameterization with mean $h_{l,a,w}^{{{{{{\mathrm{res}}}}}}}\zeta _{l,a,w}$ and variance equal to the binomial variance component $h_{l,a,w}^{{{{{{\mathrm{res}}}}}}}\zeta _{l,a,w}\left( {1 - \zeta _{l,a,w}} \right)$ multiplied by $\left( {1 + \left( {h_{l,a,w}^{{{{{{\mathrm{res}}}}}}} - 1} \right)\frac{1}{{\theta _3^{ - 1} + 1}}} \right)$ to allow for overdispersion. The priors in (12e) were chosen to place the non-Gamma in-hospital fatality rate around the empirically observed range. In (12f), we model the Gamma in-hospital fatality rate as a random effect around the non-Gamma in-hospital fatality rate. In the model, we conservatively sought to favor no dependence of in-hospital fatality rates on the hospital pressure indices, which we implemented using the horseshoe-type shrinkage prior in (12g–12i). The prior in (12k) peaks at zero and favors the least complex model with no overdispersion.

The model described through equations (9)–(12) was implemented in the Stan probabilistic computing language, is available at inst/stan-models/age_hfr_210719d.stan in the GitHub repository and was independently fitted to data from each location using cmdstanr version 0.3.0.9 (refs. ^82,83^). Because inferences were performed separately for each location, the estimates from each location provide independent support into the inferred relationships among in-hospital fatality rates, healthcare inequities and healthcare pressure. Each inference was conducted in four Hamiltonian Monte Carlo chains, each over 500 warmup iterations, and 50,500 sampling iterations. The smallest bulk effective sample size was 1,687.

From the Monte Carlo samples of the joint posterior distribution of the fitted Bayesian multi-strain fatality model, we generate the following quantities. We calculate the expected, COVID-19-attributable hospital admissions among residents in location *l*, age band *a* and week *w* for non-Gamma and Gamma variants that had no evidence of vaccination before hospitalization, respectively, by

13a $$h_{l,a,w}^{{{{{{\mathrm{res}}}}{\mbox{-}}{{{\mathrm{non }}}}}}{\mbox{-}}\Gamma } = \left( {1 - \alpha _{l,w}} \right)\pi _{l,a,w}^{{{{{{\mathrm{non}}}}}}{\mbox{-}}\Gamma }h_{l,a,w}^{{{{{{\mathrm{res}}}}}}}$$

13b $$h_{l,a,w}^{{{{{{\mathrm{res}}}}}}{\mbox{-}}\Gamma } = \alpha _{l,w}\pi _{l,a,w}^\Gamma h_{l,a,w}^{{{{{{\mathrm{res}}}}}}},$$

where $h_{l,a,w}^{{{{{{\mathrm{res}}}}}}}$ are observed and *α*_*l*,*w*_, $\pi _{l,a,w}^{{{{{{\mathrm{non}}}}}}{\mbox{-}}\Gamma }$, $\pi _{l,a,w}^\Gamma$ are from the joint posterior. The expected, COVID-19-attributable hospital admissions among residents in location *l*, age band *a* and week *w* across all variants are

14 $$h_{l,a,w}^{{{{{{\mathrm{res}}}}{\mbox{-}}{{{\mathrm{all}}}}}}} = h_{l,a,w}^{{{{{{\mathrm{res}}}}{\mbox{-}}{{{\mathrm{non}}}}}}{\mbox{-}}\Gamma } + h_{l,a,w}^{{{{{{\mathrm{res}}}}}}{\mbox{-}}\Gamma }.$$

We, thus, have that the expected share of age group *a* among hospital admissions among residents of location *l* in week *w* with non-Gamma variants is $\frac{{h_{l,a,w}^{{{{{{\mathrm{res}}}}{\mbox{-}}{{{\mathrm{non}}}}}}{\mbox{-}}\Gamma }}}{{\mathop {\sum }\nolimits_b h_{l,b,w}^{{{{{{\mathrm{res}}}}{\mbox{-}}{{{\mathrm{non}}}}}}{\mbox{-}}\Gamma }}} = \pi _{l,a,w}^{{{{{{\mathrm{non}}}}}}{\mbox{-}}\Gamma }$ and similarly for Gamma. The expected share of age group *a* among hospital admissions among residents of location *l* in week *w* across all variants is $\pi _{l,a,w} = \left( {1 - \alpha _{l,w}} \right)\pi _{l,a,w}^{{{{{{\mathrm{non}}}}}}{\mbox{-}}\Gamma } + \alpha _{l,w}\pi _{l,a,w}^\Gamma$. We calculate the expected, COVID-19-attributable deaths among hospital admissions in residents in location *l*, age band *a* and week *w* for non-Gamma and Gamma variants, respectively, by

15a $$h_{l,a,w}^{{{{{{\mathrm{res}}}}{\mbox{-}}{{{\mathrm{non}}}}}}{\mbox{-}}\Gamma {\mbox{-}}{{{{{\mathrm{D}}}}}}} = \left( {1 - \alpha _{l,w}} \right)\pi _{l,a,w}^{{{{{{\mathrm{non}}}}}}{\mbox{-}}\Gamma }h_{l,a,w}^{{{{{{\mathrm{res}}}}}}}\zeta _{l,a,w}^{{{{{{\mathrm{non}}}}}}{\mbox{-}}\Gamma }$$

15b $$h_{l,a,w}^{{{{{{\mathrm{res}}}}}}{\mbox{-}}\Gamma {\mbox{-}}{{{{{\mathrm{D}}}}}}} = \alpha _{l,w}\pi _{l,a,w}^\Gamma h_{l,a,w}^{{{{{{\mathrm{res}}}}}}}\zeta _{l,a,w}^\Gamma ,$$

where $h_{l,a,w}^{{{{{{\mathrm{res}}}}}}}$ are observed and $\alpha _{l,w}$, $\pi _{l,a,w}^{{{{{{\mathrm{non}}}}}}{\mbox{-}}\Gamma }$, $\pi _{l,a,w}^\Gamma$, $\zeta _{l,a,w}^{{{{{{\mathrm{non}}}}}}{\mbox{-}}\Gamma }$, $\zeta _{l,a,w}^\Gamma$ are from the joint posterior. The expected, COVID-19-attributable deaths among hospital admissions in residents in location *l*, age band *a* and week *w* for all variants are

16 $$h_{l,a,w}^{{{{{{\mathrm{res}}}}{\mbox{-}}{{{\mathrm{all}}}}{\mbox{-}}{{{\mathrm{D}}}}}}} = h_{l,a,w}^{{{{{{\mathrm{res}}}}{\mbox{-}}{{{\mathrm{non}}}}}}{\mbox{-}}\Gamma {{\mbox{-}}{{{{\mathrm{D}}}}}}} + h_{l,a,w}^{{{{{{\mathrm{res}}}}}}{\mbox{-}}\Gamma {\mbox{-}}{{{{{\mathrm{D}}}}}}}.$$

To compare COVID-19 in-hospital fatality rates across locations, we define the overall in-hospital fatality rate in location *l* and week *w* in an age-standardized population that adjusts for differences in age composition across locations. Specifically, we calculate

17 $$\zeta _{l,w}^{{{{{{\mathrm{age}}}}{\mbox{-}}{{{\mathrm{std}}}}}}} = \mathop {\sum }\limits_a \frac{{n_a^{{{{{{\mathrm{cities}}}}}}}}}{{\mathop {\sum }\nolimits_b n_b^{{{{{{\mathrm{cities}}}}}}}}}\zeta _{l,a,w},$$

where $n_a^{{{{{{\mathrm{cities}}}}}}}$ is the population size in age band *a* across all cities considered, and $\zeta _{l,a,w}$ is from the joint posterior. Then, for each location, we define the week w* with lowest in-hospital fatality rate as the week that minimizes the posterior median of (17),

18 $$w_l^ \star = {{{{{\mathrm{argmin}}}}}}_{w \in 1:W_l^{{{{{{\mathrm{detect}}}}}}{\mbox{-}}\Gamma }}\left( {{{{{{\mathrm{posterior}}}}}}\,{{{{{\mathrm{median}}}}}}\,{{{{{\mathrm{of}}}}}}\,\zeta _{l,w}^{{{{{{\mathrm{age}}}}{\mbox{-}}{{{\mathrm{std}}}}}}}} \right),$$

and calculate the lowest, age-standardized COVID-19 in-hospital fatality rates before the first detection of Gamma through

19 $$\zeta _l^{{{{{{\mathrm{lowest}}}}}}} = \zeta _{l,w_l^ \star }^{{{{{{\mathrm{age}}}}{\mbox{-}}{{{\mathrm{std}}}}}}}.$$

We recall that the healthcare pressure indices are standardized and evaluate to zero in the reference week of each location—that is, the week before the first detection of Gamma with lowest empirical, age-standardized in-hospital fatality rate. The week $w_l^ \star$ typically corresponds to the week with lowest empirical, age-standardized in-hospital fatality rate, and so $\zeta _l^{{{{{{\mathrm{lowest}}}}}}}$ is evaluated when the healthcare pressure effect $X_{l,w}\beta _l$ is zero. The estimated Gamma frequencies *α*_*l*,*w*_ are, of course, also very small before the first detection of Gamma, and so the lowest, age-standardized COVID-19 in-hospital fatality rate are to good approximation given by

20 $$\zeta _l^{{{{{{\mathrm{lowest}}}}}}} \approx \mathop {\sum }\limits_a \frac{{n_a^{{{{{{\mathrm{cities}}}}}}}}}{{\mathop {\sum }\nolimits_b n_b^{{{{{{\mathrm{cities}}}}}}}}}{{{{{\mathrm{logit}}}}}}^{ - 1}\left( {\eta _{l,a}^{{{{{{\mathrm{non}}}}}}{\mbox{-}}\Gamma }} \right),$$

where $\eta _{l,a}^{{{{{{\mathrm{non}}}}}}{\mbox{-}}\Gamma }$ are the intercept terms in our regression (6) and from the joint posterior. Then, to compare (19) across locations, we find the location with overall lowest age-standardised in-hospital fatality rate by

21 $$l^ \star = {{{{{\mathrm{argmin}}}}}}_l\,\zeta _l^{{{{{{\mathrm{lowest}}}}}}},$$

and compute for all other locations *l* the ratio

22 $$\zeta _l^{{{{{{\mathrm{lowest}}}}{\mbox{-}}{{{\mathrm{ratio}}}}}}} = \zeta _l^{{{{{{\mathrm{lowest}}}}}}}\big/\zeta _{l^ \star }^{{{{{{\mathrm{lowest}}}}}}}.$$

We interpret (22) as the location effect on COVID-19 in-hospital fatality rates. Because of (20), the location effect does not include contributions attributable to the healthcare pressure indices ($X_{l,w}\beta _l$) nor any contributions attributable to the non-parametric Gamma effects ($\eta _{l,a}^\Gamma$) on in-hospital fatality rates.

To compare time trends to in-hospital fatality rates in each location, we calculate for each location the multiplicative effect of changes in healthcare demand and resources in week *w* in location *l* by

23 $$\zeta _{l,w}^{{{{{{\mathrm{multiplier}}}}}}} = \mathop {\sum }\limits_a \frac{{n_a^{{{{{{\mathrm{cities}}}}}}}}}{{\mathop {\sum }\nolimits_b n_b^{{{{{{\mathrm{cities}}}}}}}}}\left( {\zeta _{l,a,w}^{{{{{{\mathrm{non}}}}}}{\mbox{-}}\Gamma } \big/\zeta _{l,a,w_l^ \star }^{{{{{{\mathrm{non}}}}}}{\mbox{-}}\Gamma }} \right),$$

where $\zeta _{l,a,w}^{{{{{{\mathrm{non}}}}}}{\mbox{-}}\Gamma }$ are from the joint posterior. Recalling equation (6a), we have

24 $$\zeta _{l,w}^{{{{{{\mathrm{multiplier}}}}}}} \approx \mathop {\sum }\limits_a \frac{{n_a^{{{{{{\mathrm{cities}}}}}}}}}{{\mathop {\sum }\nolimits_b n_b^{{{{{{\mathrm{cities}}}}}}}}}\frac{{{{{{{\mathrm{logit}}}}}}^{ - 1}\left( {\eta _{l,a}^{{{{{{\mathrm{non}}}}}}{\mbox{-}}\Gamma } + X_{l,w}\beta _l} \right)}}{{{{{{{\mathrm{logit}}}}}}^{ - 1}\left( {\eta _{l,a}^{{{{{{\mathrm{non}}}}}}{\mbox{-}}\Gamma }} \right)}}.$$

Here, the approximation is because the Gamma frequencies *α*_*l,w*_ are very small but not exactly zero and because the week in which the loess-smoothed fatality rates are lowest may not exactly coincide with the week $w_l^ \star$ in which the model-based fatality rates are lowest. We interpret (23) as the healthcare pressure effect on COVID-19 in-hospital fatality rates. Because of (24), the healthcare pressure effect does not include contributions attributable to the non-parametric Gamma effects ($\eta _{l,a}^\Gamma$) on in-hospital fatality rates and corresponds to the multiplier to the minimum fatality rates in each location that is associated with the healthcare pressure indices ($X_{l,w}\beta _l$).

Finally, we describe the effect of Gamma on in-hospital fatality rates in the model. Standardizing across age bands, we calculate the ratio in Gamma versus non-Gamma in-hospital fatality rates in location *l* by

25 $$\zeta _l^{\Gamma - {{{{{\mathrm{ratio}}}}}}} = \mathop {\sum }\limits_a \frac{{n_a^{{{{{{\mathrm{cities}}}}}}}}}{{\mathop {\sum }\nolimits_b n_b^{{{{{{\mathrm{cities}}}}}}}}}\zeta _{l,a,w_l^ \star }^\Gamma /\zeta _{l,a,w_l^ \star }^{{{{{{\mathrm{non}}}}}}{\mbox{-}}\Gamma },$$

where $\zeta _{l,a,w_l^ \star }^\Gamma$, $\zeta _{l,a,w_l^ \star }^{{{{{{\mathrm{non}}}}}}{\mbox{-}}\Gamma }$ are from the joint posterior. We interpret (25) as the Gamma effect on in-hospital fatality rates. It is again helpful to recall equation (6), which shows that we can approximate the Gamma effect through

26 $$\zeta _l^{\Gamma - {{{{{\mathrm{ratio}}}}}}} \approx \mathop {\sum }\limits_a \frac{{n_a^{{{{{{\mathrm{cities}}}}}}}}}{{\mathop {\sum }\nolimits_b n_b^{{{{{{\mathrm{cities}}}}}}}}}\frac{{{{{{{\mathrm{logit}}}}}}^{ - 1}\left( {\eta _{l,a}^{{{{{{\mathrm{non}}}}}}{\mbox{-}}\Gamma } + \eta _{l,a}^\Gamma } \right)}}{{{{{{{\mathrm{logit}}}}}}^{ - 1}\left( {\eta _{l,a}^{{{{{{\mathrm{non}}}}}}{\mbox{-}}\Gamma }} \right)}}.$$

The ratio $\zeta _l^{\Gamma - {{{{{\mathrm{ratio}}}}}}}$ does not include contributions attributable to the healthcare pressure indices ($X_{l,w}\beta _l$) and corresponds to the multiplier to the minimum fatality rates in each location that is associated with the non-parametric Gamma effects ($\eta _{l,a}^{{{{{{\mathrm{non}}}}}}{\mbox{-}}\Gamma }$).

To quantify the effect that the observed fluctuations in COVID-19-attributable in-hospital rates had on the death toll in the 14 state capitals, we performed two counterfactual analyses. The aim of the first counterfactual (Scenario 1) was to estimate how many COVID-19-attributable deaths could have been avoided with sufficient healthcare resources so that healthcare pressures would not have resulted in shocks in in-hospital fatality rates in each city. We implemented Scenario 1 by predicting deaths in each city under the minimum in-hospital fatality rate that was observed in each age group in that city. Specifically, for each location *l*, we considered the estimated age-specific in-hospital fatality rates (8) in the week (18) and computed

27 $$h_l^{{{{{{\mathrm{hyp1}}}}{\mbox{-}}{{{\mathrm{res}}}}{\mbox{-}}{{{\mathrm{D}}}}}}} = \mathop {\sum }\limits_{w = 1}^{W_l} \mathop {\sum }\limits_{a \in {{{{{\mathcal{A}}}}}}} h_{l,a,w}^{{{{{{\mathrm{res}}}}}}}\zeta _{l,a,w_l^ \star },$$

where the week indices range over the entire observation period. We define the expected COVID-19-attributable deaths that could have been avoided in the absence of healthcare pressures in location *l* during the observation period relative to this hypothetical scenario by

28 $$h_l^{{{{{{\mathrm{hyp1}}}}{\mbox{-}}{{{\mathrm{avoidable}}}}{\mbox{-}}{{{\mathrm{D}}}}}}} = \left( {\mathop {\sum }\limits_{w = 1}^{W_l} \mathop {\sum }\limits_{a \in {{{{{\mathcal{A}}}}}}} h_{l,a,w}^{{{{{{\mathrm{res}}}}{\mbox{-}}{{{\mathrm{adj}}}}{\mbox{-}}{{{\mathrm{D}}}}}}}} \right) - h_l^{{{{{{\mathrm{hyp1}}}}{\mbox{-}}{{{\mathrm{res}}}}{\mbox{-}}{{{\mathrm{D}}}}}}}.$$

Similarly, we define the expected percentage reduction in COVID-19-attributable deaths relative to this hypothetical scenario by

29 $$p_l^{{{{{{\mathrm{hyp1}}}}{\mbox{-}}{{{\mathrm{avoidable}}}}{\mbox{-}}{{{\mathrm{D}}}}}}} = 1 - \frac{{h_l^{{{{{{\mathrm{hyp1}}}}{\mbox{-}}{{{\mathrm{res}}}}{\mbox{-}}{{{\mathrm{D}}}}}}}}}{{\mathop {\sum }\nolimits_{w = 1}^{W_l} \mathop {\sum }\nolimits_{a \in {{{{{\mathcal{A}}}}}}} h_{l,a,w}^{{{{{{\mathrm{res}}}}{\mbox{-}}{{{\mathrm{adj}}}}{\mbox{-}}{{{\mathrm{D}}}}}}}}}.$$

The aim of the second counterfactual (Scenario 2) was to estimate how many COVID-19-attributable deaths could have been avoided with sufficient healthcare resources and without healthcare inequities across the cities. We implemented Scenario 2 by predicting deaths in each city under the minimum in-hospital fatality rate seen across all cities, which we observed in Belo Horizonte. Specifically, we considered the location $l^{ \star \star }$ in which we found the lowest age-standardized in-hospital fatality rate before Gamma’s detection in each location

30 $$l^{ \star \star } = {{{{{\mathrm{argmin}}}}}}_l\left( {\mathop {{{{{{{\mathrm{min}}}}}}}}\limits_{w \in 1:W_l^{{{{{{\mathrm{detect}}}}}}{\mbox{-}}\Gamma }} \left( {{{{{{\mathrm{posterior}}}}}}\,{{{{{\mathrm{median}}}}}}\,{{{{{\mathrm{of}}}}}}\,\xi _{l,w}^{{{{{{\mathrm{age}}}}{\mbox{-}}{{{\mathrm{std}}}}}}}} \right)} \right)$$

and computed

31 $$h_l^{{{{{{\mathrm{hyp2}}}}{\mbox{-}}{{{\mathrm{res}}}}{\mbox{-}}{{{\mathrm{D}}}}}}} = \mathop {\sum }\limits_{w = 1}^{W_l} \mathop {\sum }\limits_{a \in {{{{{\mathcal{A}}}}}}} h_{l,a,w}^{{{{{{\mathrm{res}}}}}}}\zeta _{l^{ \star \star },a,w^ \star },$$

where, again, the week indices range over the entire observation period. In analogy to the first counterfactual, we then calculate

32 $$h_l^{{{{{{\mathrm{hyp2}}}}{\mbox{-}}{{{\mathrm{avoidable}}}}{\mbox{-}}{{{\mathrm{D}}}}}}} = \left( {\mathop {\sum }\limits_{w = 1}^{W_l} \mathop {\sum }\limits_{a \in {{{{{\mathcal{A}}}}}}} h_{l,a,w}^{{{{{{\mathrm{res}}}}{\mbox{-}}{{{\mathrm{adj}}}}{\mbox{-}}{{{\mathrm{D}}}}}}}} \right) - h_l^{{{{{{\mathrm{hyp2}}}}{\mbox{-}}{{{\mathrm{res}}}}{\mbox{-}}{{{\mathrm{D}}}}}}},$$

and then the expected percentage reduction

33 $$p_l^{{{{{{\mathrm{hyp2}}}}{\mbox{-}}{{{\mathrm{avoidable}}}}{\mbox{-}}{{{\mathrm{D}}}}}}} = 1 - \frac{{h_l^{{{{{{\mathrm{hyp2}}}}{\mbox{-}}{{{\mathrm{res}}}}{\mbox{-}}{{{\mathrm{D}}}}}}}}}{{\mathop {\sum }\nolimits_{w = 1}^{W_l} \mathop {\sum }\nolimits_{a \in {{{{{\mathcal{A}}}}}}} h_{l,a,w}^{{{{{{\mathrm{res}}}}{\mbox{-}}{{{\mathrm{adj}}}}{\mbox{-}}{{{\mathrm{D}}}}}}}}}.$$

### Reporting Summary

Further information on research design is available in the Nature Research Reporting Summary linked to this article.

## Online content

Any methods, additional references, Nature Research reporting summaries, source data, extended data, supplementary information, acknowledgements, peer review information; details of author contributions and competing interests; and statements of data and code availability are available at 10.1038/s41591-022-01807-1.

## Supplementary information

Supplementary Information Supplementary Tables 1–7 and Figs. 1–19.

Reporting Summary

## Data Availability

All data necessary for the replication of our results are available at https://github.com/CADDE-CENTRE/covid19_brazil_hfr (10.5281/zenodo.6373425). These datasets were derived from the following public domain resources: the SIVEP-Gripe platform (https://opendatasus.saude.gov.br/dataset/srag-2020, https://opendatasus.saude.gov.br/dataset/srag-2021-e2022); the Brazilian Civil Registry (https://transparencia.registrocivil.org.br/); the Brazilian Ministry of Health (https://opendatasus.saude.gov.br/dataset/covid-19-vacinacao); the National Household Sample Survey COVID-19 (https://www.ibge.gov.br/estatisticas/sociais/populacao/9171-pesquisa-nacional-poramostra-de-domicilios-continua-mensal.html?=&t=o-que-e); and Brazil’s National Register of Health Facilities (https://datasus.saude.gov.br/transferencia-de-arquivos/). Data from the Brazilian Civil Registry were accessed on 9 August 2021 through https://github.com/capyvara/brazil-civil-registry-data. The downloaded and processed versions are also available in our GitHub repository at inst/data/SIVEP_hospital_31-01-2022-all.rds; inst/data/registry_covid_detailed_09-08-2021.csv; inst/data/aggregated_vaccinations_210805.rds; inst/data/PNADc_populationpyramids_210617.csv; inst/data/genomic_data_210702.csv; and inst/data/IPEA_ICUbeds_physicians_210928.csv.

## Code availability

The code necessary to reproduce the analysis are available in the GitHub repository at https://github.com/CADDE-CENTRE/COVID-19_brazil_hfr (10.5281/zenodo.6373425).

## References

[1] Davies NG (2021). Estimated transmissibility and impact of SARS-CoV-2 lineage B.1.1.7 in England. Science.

[2] Tegally H (2021). Detection of a SARS-CoV-2 variant of concern in South Africa. Nature.

[3] Faria NR (2021). Genomics and epidemiology of the P.1 SARS-CoV-2 lineage in Manaus, Brazil. Science.

[4] Volz E (2021). Assessing transmissibility of SARS-CoV-2 lineage B.1.1.7 in England. Nature.

[5] Dyer O (2021). Covid-19: South Africa’s surge in cases deepens alarm over omicron variant. BMJ.

[6] Naveca FG (2021). COVID-19 in Amazonas, Brazil, was driven by the persistence of endemic lineages and P.1 emergence. Nat. Med..

[7] Fujino T (2021). Novel SARS-CoV-2 variant in travelers from Brazil to Japan. Emerg. Infect. Dis..

[8] Harvey WT (2021). SARS-CoV-2 variants, spike mutations and immune escape. Nat. Rev. Microbiol..

[9] Souza WM (2021). Neutralisation of SARS-CoV-2 lineage P.1 by antibodies elicited through natural SARS-CoV-2 infection or vaccination with an inactivated SARS-CoV-2 vaccine: an immunological study. Lancet Microbe.

[10] Martins AF (2021). Detection of SARS-CoV-2 lineage P.1 in patients from a region with exponentially increasing hospitalisation rate, February 2021 Rio Grande do Sul, Southern Brazil. Euro Surveill..

[11] Walker PGT (2020). The impact of COVID-19 and strategies for mitigation and suppression in low- and middle-income countries. Science.

[12] SRAG 2020 - Banco de Dados de Síndrome Respiratória Aguda Grave. *openDataSUS* https://opendatasus.saude.gov.br/dataset/srag-2020 (2020).

[13] SRAG 2021 e 2022 - Banco de Dados de Síndrome Respiratória Aguda Grave. *openDataSUS* https://opendatasus.saude.gov.br/dataset/srag-2021-e-2022 (2021).

[14] Castro MC (2019). Brazil’s unified health system: the first 30 years and prospects for the future. Lancet.

[15] de Oliveira Andrade, R. COVID-19 is causing the collapse of Brazil’s national health service. *BMJ* **370**, m3032 (2020). 10.1136/bmj.m3032 32732376

[16] de Souza WM (2020). Epidemiological and clinical characteristics of the COVID-19 epidemic in Brazil. Nat. Hum. Behav..

[17] Ranzani OT (2021). Characterisation of the first 250 000 hospital admissions for COVID-19 in Brazil: a retrospective analysis of nationwide data. Lancet Respir. Med..

[18] Li, S. L. et al. Higher risk of death from COVID-19 in low-income and non-White populations of São Paulo, Brazil. *BMJ Glob. Health* **6**, e004959 (2021). 10.1136/bmjgh-2021-004959 PMC8094342 33926892

[19] Albuquerque MVD, Ribeiro LHL (2021). Inequality, geographic situation, and meanings of action in the COVID-19 pandemic in Brazil. Cad. Saude Publica.

[20] Rocha TAH (2018). National registry of health facilities: data reliability evidence. Cien. Saude Colet..

[21] de Oliveira, M. H. S., Lippi, G. & Henry, B. M. Sudden rise in COVID-19 case fatality among young and middle-aged adults in the south of Brazil after identification of the novel B.1.1.28.1 (P.1) SARS-CoV-2 strain: analysis of data from the state of Parana. Preprint at *medRxiv*10.1101/2021.03.24.21254046 (2021).

[22] Freitas ARR (2021). The emergence of novel SARS-CoV-2 variant P. 1 in Amazonas (Brazil) was temporally associated with a change in the age and sex profile of COVID-19 mortality: a population based ecological study. Lancet Reg. Health Am..

[23] Shu Y, McCauley J (2017). GISAID: Global Initiative on Sharing All Influenza Data—from vision to reality. Euro Surveill..

[24] Fellows M (2021). Under-reporting of COVID-19 cases among indigenous peoples in Brazil: a new expression of old inequalities. Front. Psychiatry.

[25] Veiga E Silva L (2020). COVID-19 mortality underreporting in Brazil: analysis of data from government internet portals. J. Med. Internet Res..

[26] Brazeau, N. et al. *Report 34: COVID-19 Infection Fatality Ratio: Estimates from Seroprevalence* (Imperial College London, 2020); 10.25561/83545

[27] Rocha R (2021). Effect of socioeconomic inequalities and vulnerabilities on health-system preparedness and response to COVID-19 in Brazil: a comprehensive analysis. Lancet Glob. Health.

[28] Funcia FR (2019). Underfunding and federal budget of SUS: preliminary references for additional resource allocation. Cien. Saude Colet..

[29] Pereira RHM (2021). Geographic access to COVID-19 healthcare in Brazil using a balanced float catchment area approach. Soc. Sci. Med..

[30] Castro MC (2021). Reduction in life expectancy in Brazil after COVID-19. Nat. Med..

[31] Clark A (2020). Global, regional, and national estimates of the population at increased risk of severe COVID-19 due to underlying health conditions in 2020: a modelling study. Lancet Glob. Health.

[32] Castro MC (2021). Spatiotemporal pattern of COVID-19 spread in Brazil. Science.

[33] de Souza Santos AA (2021). Dataset on SARS-CoV-2 non-pharmaceutical interventions in Brazilian municipalities. Sci. Data.

[34] Brito, A. F. et al. Global disparities in SARS-CoV-2 genomic surveillance. Preprint at *medRxiv*10.1101/2021.08.21.21262393 (2021).

[35] Sheikh A, McMenamin J, Taylor B, Robertson C (2021). SARS-CoV-2 Delta VOC in Scotland: demographics, risk of hospital admission, and vaccine effectiveness. Lancet.

[36] Davies NG (2021). Increased mortality in community-tested cases of SARSCoV-2 lineage B.1.1.7. Nature.

[37] Nicolelis MA, Raimundo RL, Peixoto PS, Andreazzi CS (2021). The impact of super-spreader cities, highways, and intensive care availability in the early stages of the COVID-19 epidemic in Brazil. Sci. Rep..

[38] Noronha, K. V. M. D. S. et al. Pandemia por COVID-19 no Brasil: análise da demanda e da oferta de leitos hospitalares e equipamentos de ventilação assistida segundo diferentes cenários. *Cad. Saude Publica*10.1590/0102-311X00115320 (2020). 10.1590/0102-311X00115320 32578805

[39] Machado FR (2017). The epidemiology of sepsis in Brazilian intensive care units (the Sepsis PREvalence Assessment Database, SPREAD): an observational study. Lancet Infect. Dis..

[40] Malta, M., Strathdee, S. A. & Garcia, P. J. The Brazilian tragedy: where patients living at the Earth’s lungs die of asphyxia, and the fallacy of herd immunity is killing people. *EClinicalMedicine* **32**, 100757 (2021). 10.1016/j.eclinm.2021.100757 PMC7890368 33659889

[41] Leite SN (2021). Management of the health workforce in facing COVID-19: disinformation and absences in Brazils public policies. Cien. Saude Colet..

[42] Mendes, Á., Carnut, L. & Guerra, L. D. D. S. Primary health care federal funding in the Unified Health System: old and new dilemmas. *Saúde em Debate*10.1590/0103-11042018S115 (2018).

[43] Grint, D. J. et al. Severity of SARS-CoV-2 alpha variant (B.1.1.7) in England. *Clin. Infect. Dis.*10.1093/cid/ciab754 (2021). 10.1093/cid/ciab754 PMC8522415 34487522

[44] Haldane V (2021). Health systems resilience in managing the COVID-19 pandemic: lessons from 28 countries. Nat. Med..

[45] Hiam L, Yates R (2021). Will the COVID-19 crisis catalyse universal health reforms?. Lancet.

[46] Lal A, Erondu NA, Heymann DL, Gitahi G, Yates R (2021). Fragmented health systems in COVID-19: rectifying the misalignment between global health security and universal health coverage. Lancet.

[47] Baum F (2021). Explaining covid-19 performance: what factors might predict national responses?. BMJ.

[48] Lytras, T. & Tsiodras, S. Total patient load, regional disparities and in-hospital mortality of intubated COVID-19 patients in Greece, from September 2020 to May 2021. *Scan. J. Public Health* 14034948211059968 (2021). 10.1177/14034948211059968 34903101

[49] Kadri SS (2021). Association between caseload surge and COVID-19 survival in 558 US hospitals, March to August 2020. Ann. Intern. Med..

[50] Rossman, H. et al. Hospital load and increased COVID-19 related mortality in Israel. *Nat. Commun.* **12**, 1904 (2021). 10.1038/s41467-021-22214-z PMC7997985 33771988

[51] Sirleaf EJ, Clark H (2021). Report of the Independent Panel for Pandemic Preparedness and Response: making COVID-19 the last pandemic. Lancet.

[52] *Pesquisa Nacional por Amostra de Domicílios - PNAD COVID19* (IBGE, 2020); https://www.ibge.gov.br/estatisticas/sociais/trabalho/27946-divulgacao-semanal-pnadcovid1.html?=&t=microdados

[53] Cadastro Nacional de Estabelecimentos de Saúde. *DataSUS* https://datasus.saude.gov.br/transferencia-de-arquivos/ (2021).

[54] Resolution No. 2.271 of February 14, 2020 (2020); https://www.in.gov.br/web/dou/-/resolucao-n-2.271-de-14-de-fevereiro-de2020-253606068

[55] Katoh K, Standley DM (2013). MAFFT multiple sequence alignment software version 7: improvements in performance and usability. Mol. Biol. Evol..

[56] Minh BQ (2020). IQ-TREE 2: new models and efficient methods for phylogenetic inference in the genomic era. Mol. Biol. Evol..

[57] Jukes, T. H. et al. in *Mammalian Protein Metabolism* (eds Munro, H. N. & Allison, J. B.) 21–132 (Elsevier, 1969).

[58] Rambaut, A., Lam, T. T., Max Carvalho, L. & Pybus, O. G. Exploring the temporal structure of heterochronous sequences using TempEst (formerly Path-O-Gen). *Virus Evol*. **2**, vew007 (2016). 10.1093/ve/vew007 PMC4989882 27774300

[59] du Plessis L (2021). Establishment and lineage dynamics of the SARS-CoV-2 epidemic in the UK. Science.

[60] Gutierrez B (2021). Genomic epidemiology of SARS-CoV2 transmission lineages in Ecuador. Virus Evol..

[61] Suchard, M. A. et al. Bayesian phylogenetic and phylodynamic data integration using BEAST 1.10. *Virus Evol.* **4**, vey016 (2018). 10.1093/ve/vey016 PMC6007674 29942656

[62] Hasegawa M, Kishino H, Yano T-A (1985). Dating of the human–ape splitting by a molecular clock of mitochondrial DNA. J. Mol. Evol..

[63] Hill V, Baele G (2019). Bayesian estimation of past population dynamics in BEAST 1.10 using the Skygrid coalescent model. Mol. Biol. Evol..

[64] Rambaut A, Drummond AJ, Xie D, Baele G, Suchard MA (2018). Posterior summarization in Bayesian phylogenetics using tracer 1.7. Syst. Biol..

[65] McCrone, J. T. BEAST v1.10.5 pre-release of ThorneyTreeLikelihood v0.1.1. *GitHub* https://github.com/beast-dev/beast-mcmc/releases/tag/v1.10.5pre_thorney_v0.1.1 (2021).

[66] Volz, E. M. & Frost, S. D. W. Scalable relaxed clock phylogenetic dating. *Virus Evol*. **3**, vex025 (2017).

[67] Didelot X, Siveroni I, Volz EM (2021). Additive uncorrelated relaxed clock models for the dating of genomic epidemiology phylogenies. Mol. Biol. Evol..

[68] Lemey P, Rambaut A, Drummond AJ, Suchard MA (2009). Bayesian phylogeography finds its roots. PLoS Comput. Biol..

[69] Minin VN, Suchard MA (2008). Counting labeled transitions in continuous-time Markov models of evolution. J. Math. Biol..

[70] Minin, V. N. & Suchard, M. A. Fast, accurate and simulation-free stochastic mapping. *Phil. Trans. R. Soc. B* **363**, 3985–3995 (2008). 10.1098/rstb.2008.0176 PMC2607419 18852111

[71] O’Brien JD, Minin VN, Suchard MA (2009). Learning to count: robust estimates for labeled distances between molecular sequences. Mol. Biol. Evol..

[72] Lemey P (2021). Untangling introductions and persistence in COVID-19 resurgence in Europe. Nature.

[73] *Genomic Sequencing of SARS-CoV-2: A Guide to Implementation for Maximum Impact on Public Health* (WHO, 2021); https://www.who.int/publications/i/item/9789240018440

[74] artic-network/fieldbioinformatics. *GitHub* https://github.com/artic-network/fieldbioinformatics (2022).

[75] O’Toole, Á., Hill, V., McCrone, J. T., Scher, E. & Rambaut, A. Pangolin COVID-19 Lineage Assigner (2022); https://pangolin.cog-uk.io/

[76] Rambaut A (2020). A dynamic nomenclature proposal for SARS-CoV-2 lineages to assist genomic epidemiology. Nat. Microbiol..

[77] Ranzani, O. T. et al. Effectiveness of the CoronaVac vaccine in older adults during a gamma variant associated epidemic of COVID-19 in Brazil: test negative case–control study. *BMJ* **374**, n2016 (2021). 10.1136/bmj.n2015 PMC8377801 34417194

[78] Voysey M (2021). Single-dose administration and the influence of the timing of the booster dose on immunogenicity and efficacy of ChAdOx1 nCoV-19 (AZD1222) vaccine: a pooled analysis of four randomised trials. Lancet.

[79] Madhi SA (2021). Efficacy of the ChAdOx1 nCoV-19 COVID-19 vaccine against the B.1.351 variant. N. Engl. J. Med..

[80] Clemens, S. A. C. et al. Efficacy of ChAdOx1 nCoV-19 (AZD1222) vaccine against SARS-CoV-2 lineages circulating in Brazil. *Nat. Commun*. **12**, 5861 (2021). 10.1038/s41467-021-25982-w PMC8494913 34615860

[81] Khoury DS (2021). Neutralizing antibody levels are highly predictive of immune protection from symptomatic SARS-CoV-2-infection. Nat. Med..

[82] Carpenter B (2017). Stan: a probabilistic programming language. J. Stat. Softw..

[83] Hoffman MD, Gelman A (2014). The No-U-Turn sampler: adaptively setting path lengths in Hamiltonian Monte Carlo. J. Mach. Learn. Res..

