# Supplementary material for: Spatial and temporal fluctuations in COVID-19 fatality rates in Brazilian hospitals
Source: Nat Med. 2022 May 10;28(7):1476–85. doi: 10.1038/s41591-022-01807-1 (PMC9307484; doi:10.1038/s41591-022-01807-1)
Supplement: Supplementary file 1 — Supplementary Tables 1–7 and Figs. 1–19. [file 41591_2022_1807_MOESM1_ESM.pdf]

nature portfolio

https://doi.org/10.1038/s41591-022-01807-1

Supplementary information

Spatial and temporal fluctuations in COVID-19 fatality rates in Brazilian hospitals

In the format provided by the
authors and unedited

Supplementary Tables and Figures to

Spatial and temporal fluctuations in COVID-19 fatality rates in Brazilian hospitals

Brizzi et. al.

State capital	State	Observation period		SARS-CoV-2 Gamma variant	
		Start	End	Estimated emergence (posterior median)	First detection (observed)
Belo Horizonte	Minas Gerais	06/04/2020	26/07/2021	15/02/2021	15/12/2020
Curitiba	Paraná	02/03/2020	26/07/2021	26/01/2021	22/10/2020
Florianópolis	Santa Catarina	09/03/2020	26/07/2021	08/01/2021	11/12/2020
Goiânia	Goiás	16/03/2020	26/07/2021	19/01/2021	28/11/2020
João Pessoa	Paraíba	09/03/2020	26/07/2021	21/01/2021	20/12/2020
Macapá	Amapá	30/03/2020	26/07/2021	16/01/2021	14/12/2020
Manaus	Amazonas	24/02/2020	26/07/2021	04/12/2020	04/12/2020
Natal	Rio Grande do Norte	16/03/2020	26/07/2021	26/01/2021	06/12/2020
Porto Alegre	Rio Grande do Sul	02/03/2020	26/07/2021	10/01/2021	21/11/2020
Porto Velho	Rondônia	30/03/2020	26/07/2021	18/01/2021	25/12/2020
Rio de Janeiro	Rio de Janeiro	16/03/2020	26/07/2021	19/01/2021	29/11/2020
Salvador	Bahia	16/03/2020	26/07/2021	28/12/2020	02/12/2020
São Luís	Maranhão	24/02/2020	26/07/2021	19/01/2021	04/12/2020
São Paulo	São Paulo	20/01/2020	26/07/2021	01/02/2021	01/11/2020

Supplementary Table 1: Sampling frame of study.

Location	Reported COVID-19 attributable in-hospital deaths†				Underreporting-adjusted COVID-19 attributable in-hospital deaths‡			
	Total	Ages			Total	Ages		
		0-49	50-74	75+		0-49	50-74	75+
Belo Horizonte	7640	808	3680	3152	7691	817	3705	3169
Curitiba	7535	1162	3941	2432	7535	1162	3941	2432
Florianópolis	882	85	441	356	892	86	446	360
Goiânia	6103	1006	3110	1987	6245	1036	3181	2028
João Pessoa	2810	508	1326	976	3635	674	1730	1231
Macapá	992	240	507	245	1011	243	520	248
Manaus	10006	1922	5390	2694	10168	1961	5483	2724
Natal	3059	467	1448	1144	3512	561	1688	1263
Porto Alegre	5133	517	2625	1991	5265	541	2701	2023
Porto Velho	2156	468	1200	488	2472	561	1361	550
Rio de Janeiro	23398	3116	11537	8745	28052	3947	13988	10117
Salvador	7667	1206	3754	2707	8508	1402	4206	2900
São Luís	2163	372	1049	742	2546	428	1256	862
São Paulo	40744	5483	20301	14960	42768	5795	21302	15671

† COVID-19 attributable in-hospital deaths were defined as deaths in hospitalised unvaccinated residents with PCR confirmed or clinically diagnosed COVID-19 infection, or severe respiratory infection with no other confirmed cause. Data capture admissions and deaths until 26 July 2021.

‡ To reported counts were added expected fatal outcomes in COVID-19 attributable hospital admissions in unvaccinated residents with unreported outcomes (see Methods).

Supplementary Table 2: Reported and underreporting-adjusted COVID-19 attributable in-hospital deaths.

City	Longest observed duration of COVID-19 in-hospital fatality rates above 50% (in weeks, by age group shown in years)										
	0-15	16-29	30-39	40-49	50-59	60-69	70-74	75-79	80-84	85-89	90+
Belo Horizonte	0	0	0	0	0	0	0	0	0	0	0
Curitiba	0	0	0	0	0	0	3	0	2	0	6
Florianópolis	0	0	0	0	0	0	0	8	7	5	14
Goiânia	0	0	0	0	0	0	7	9	12	22	32
João Pessoa	0	0	0	0	0	2	8	18	44	24	19
Macapá	0	7	0	8	9	11	14	18	23	53	31
Manaus	0	0	0	1	4	9	10	14	51	26	51
Natal	0	0	0	0	1	24	16	25	30	48	47
Porto Alegre	0	0	0	0	0	6	9	10	12	56	62
Porto Velho	0	0	0	0	0	8	25	30	16	48	64
Rio de Janeiro	0	0	0	0	0	0	12	52	72	72	70
Salvador	0	0	0	0	1	0	9	10	13	28	29
São Luís	1	0	0	0	0	1	2	14	11	17	12
São Paulo	0	1	0	0	0	4	7	2	8	20	20

Supplementary Table 3: Longest observed duration of COVID-19 in-hospital fatality rates above 50%.

	Date	Healthcare resources per 100,000 population†						
		Belo Horizonte	Curitiba	Florianó-polis	Goiânia	João Pessoa	Macapá	Manaus
Intensive Care	March 2020	2	3	3	2	7	0	2
Specialists	July 2021	2 (0%)	3 (0%)	4 (33%)	2 (0%)	6 (-14%)	0	2 (0%)
Nurses	March 2020	249	185	251	151	212	129	114
	July 2021	306 (23%)	228 (23%)	299 (19%)	180 (19%)	238 (12%)	164 (27%)	152 (33%)
Physicians	March 2020	631	482	615	368	309	124	155
	July 2021	674 (7%)	520 (8%)	686 (12%)	394 (7%)	345 (12%)	139 (12%)	166 (7%)
Physiotherapists	March 2020	74	65	86	50	76	47	19
	July 2021	92 (24%)	84 (29%)	117 (36%)	59 (18%)	90 (18%)	56 (19%)	29 (53%)
Nurse assistants	March 2020	584	356	479	371	309	318	290
	July 2021	723 (24%)	491 (38%)	601 (25%)	427 (15%)	367 (19%)	350 (10%)	328 (13%)
CC Beds	March 2020	41	41	35	51	27	10	11
	July 2021	56 (37%)	63 (54%)	54 (54%)	86 (69%)	59 (119%)	28 (180%)	28 (155%)
CC Beds with	March 2020	40	41	30	36	23	9	11
ventilator	July 2021	55 (38%)	62 (51%)	51 (70%)	70 (94%)	54 (135%)	25 (178%)	26 (136%)
ICU Beds	March 2020	34	33	24	32	22	6	9
	July 2021	48 (41%)	56 (70%)	46 (92%)	66 (106%)	51 (132%)	22 (267%)	24 (167%)
Ventilators	March 2020	83	79	86	77	57	21	38
	July 2021	101 (22%)	112 (42%)	108 (26%)	113 (47%)	87 (53%)	41 (95%)	58 (53%)
		Natal	Porto Alegre	Porto Velho	Rio de Janeiro	Salvador	São Luís	São Paulo
Intensive Care	March 2020	4	6	2	5	6	1	3
Specialists	July 2021	4 (0%)	6 (0%)	2 (0%)	6 (20%)	7 (17%)	2 (100%)	4 (33%)
Nurses	March 2020	162	290	168	185	229	223	183
	July 2021	201 (24%)	334 (15%)	250 (49%)	228 (23%)	265 (16%)	258 (16%)	219 (20%)
Physicians	March 2020	313	626	237	299	329	207	324
	July 2021	348 (11%)	654 (4%)	293 (24%)	326 (9%)	344 (5%)	229 (11%)	358 (10%)
Physiotherapists	March 2020	43	67	44	35	73	53	41
	July 2021	55 (28%)	79 (18%)	76 (73%)	41 (17%)	80 (10%)	71 (34%)	55 (34%)
Nurse assistants	March 2020	378	624	461	291	374	497	214
	July 2021	451 (19%)	868 (39%)	608 (32%)	422 (45%)	432 (16%)	569 (14%)	271 (27%)
CC Beds	March 2020	41	55	37	44	55	37	37
	July 2021	68 (66%)	88 (60%)	86 (132%)	56 (27%)	67 (22%)	58 (57%)	52 (41%)
CC Beds with	March 2020	36	53	35	38	46	33	30
ventilator	July 2021	63 (75%)	86 (62%)	82 (134%)	50 (32%)	63 (37%)	48 (45%)	48 (60%)
ICU Beds	March 2020	27	45	31	22	42	24	18
	July 2021	53 (96%)	82 (82%)	78 (152%)	31 (41%)	60 (43%)	37 (54%)	37 (106%)
Ventilators	March 2020	75	102	68	72	83	65	66
	July 2021	123 (64%)	156 (53%)	147 (116%)	94 (31%)	109 (31%)	87 (34%)	86 (30%)

† Numbers summarise healthcare facility level microdata on personnel (nurses, nurse assistants, physiotherapists, physicians and intensive care specialists), and reported equipment (critical care beds, ICU beds, ventilators) from Brazils National Register of Health Facilities (Cadastro Nacional de Estabelecimentos de Saúde - CNES); see Methods. Values in brackets give the percent increase from March 2020 to July 2021. CC stands for critical care.

Supplementary Table 4: Healthcare resources, March 2020 to July 2021.

Healthcare pressure index	Definition†	CBO family
3-wk SARI admissions per physician	$x_{l,w}^{\text{SARI-adm-per-physician-02}} = \left(\sum_{i=0}^{2} h_{l,w+i}^{\text{SARI}}\right) \Big/ n_{l,w}^{\text{physicians}}$	2231, 2251, 2252, 2253
3-wk ICU admissions per physiotherapist	$x_{l,w}^{\text{ICU-adm-per-physiotherapist-02}} = \left(\sum_{i=0}^{2} h_{l,w+i}^{\text{ICU}}\right) \Big/ n_{l,w}^{\text{physiotherapists}}$	2236
3-wk ICU admissions per physician	$x_{l,w}^{\text{ICU-adm-per-physician-02}} = \left(\sum_{i=0}^{2} h_{l,w+i}^{\text{ICU}}\right) \Big/ n_{l,w}^{\text{physicians}}$	2231, 2251, 2252, 2253
3-wk ICU admissions per nurse assistant	$x_{l,w}^{\text{ICU-adm-per-nurse-assist-02}} = \left(\sum_{i=0}^{2} h_{l,w+i}^{\text{ICU}}\right) \Big/ n_{l,w}^{\text{nurse-assist}}$	3222
3-wk ICU admissions per nurse	$x_{l,w}^{\text{ICU-adm-per-nurse-02}} = \left(\sum_{i=0}^{2} h_{l,w+i}^{\text{ICU}}\right) \Big/ n_{l,w}^{\text{nurses}}$	2235
3-wk ICU admissions per intensive care specialist	$x_{l,w}^{\text{ICU-adm-per-intensivist-02}} = \left(\sum_{i=0}^{2} h_{l,w+i}^{\text{ICU}}\right) \Big/ n_{l,w}^{\text{intensivist}}$	225150
		CNES code
3-wk SARI admissions per ventilator	$x_{l,w}^{\text{SARI-adm-per-ventilator-02}} = \left(\sum_{i=0}^{2} h_{l,w+i}^{\text{SARI}}\right) \Big/ n_{l,w}^{\text{ventilators}}$	64
3-wk SARI admissions per critical care bed with ventilator	$x_{l,w}^{\text{SARI-adm-per-crit-care-bed-vent-02}} = \left(\sum_{i=0}^{2} h_{l,w+i}^{\text{SARI}}\right) \Big/ n_{l,w}^{\text{crit-care-beds-vent}}$	64, 95
3-wk SARI admissions per critical care bed	$x_{l,w}^{\text{SARI-adm-per-crit-care-bed-02}} = \left(\sum_{i=0}^{2} h_{l,w+i}^{\text{SARI}}\right) \Big/ n_{l,w}^{\text{crit-care-beds}}$	51, 75, 76,95
3-wk ICU admissions per ventilator	$x_{l,w}^{\text{ICU-adm-per-ventilator-02}} = \left(\sum_{i=0}^{2} h_{l,w+i}^{\text{ICU}}\right) \Big/ n_{l,w}^{\text{ventilators}}$	64
3-wk ICU admissions per ICU bed	$x_{l,w}^{\text{ICU-adm-per-ICU-bed-02}} = \left(\sum_{i=0}^{2} h_{l,w+i}^{\text{ICU}}\right) \Big/ r_{l,w}^{\text{ICU-beds}}$	51, 75, 76

† Data on hospital resources were obtained from CNES [53], and were aggregated by month and location. Data on hospital admissions were retrieved from the SIVEP-Gripe platform. Hospital personnel data were defined according to the 2002 Brazilian Classification of Occupations [Classificação Brasileira de Ocupações (CBO)], available at: https://www.ocupacoes.com.br/. Hospital equipment data were defined according to the CNES code classification; see Methods for details.

Supplementary Table 5: Definitions of the healthcare pressure indices.

Week	Manaus		Belo Horizonte		São Paulo	
	Genomes sampled	Gamma positive	Genomes sampled	Gamma positive	Genomes sampled	Gamma positive
27/05/20	2	0	-	-	-	-
02/11/20	9	0	-	-	1	0
09/11/20	2	0	-	-	-	-
16/11/20	4	0	-	-	-	-
23/11/20	-	-	-	-	1	0
30/11/20	2	1	-	-	-	-
07/12/20	7	1	-	-	-	-
14/12/20	24	7	-	-	-	-
21/12/20	37	20	-	-	4	0
28/12/20	14	8	-	-	2	0
04/01/21	46	40	2	0	3	1
11/01/21	-	-	1	0	7	5
18/01/21	-	-	-	-	7	2
25/01/21	-	-	2	0	3	1
01/02/21	-	-	3	0	1	0
08/02/21	-	-	1	0	2	1
15/02/21	-	-	3	0	14	10
22/02/21	-	-	1	0	10	9
01/03/21	-	-	37	11	5	5
08/03/21	-	-	24	16	6	6
15/03/21	-	-	-	-	2	2
22/03/21	-	-	-	-	7	6
29/03/21	-	-	-	-	1	1

Supplementary Table 6: SARS-CoV-2 sequence data from Manaus, Belo Horizonte, and São Paulo, obtained under controlled sequence sampling frames.

	Assumed efficacy against severe infection requiring hospitalisation		Notes and references
	1st dose	2nd dose	
Sinovac	31.6%	71.4%	Values taken from Ref.[77], assuming vaccine effectiveness against hospitalisation is similar to effectiveness against death.
Covishield	40%	91.4%	Estimates based on Refs.[78,79,80], and calibrating effectiveness against symptomatic infection against severe infection using Fig. 3A in Ref.[81].
Janssen and Pfizer	40%	91.4%	No effectiveness studies found in Brazil. Janssen and Pfizer contributed to 15.07% of administered by the end of the study period. Hence, used same values as for Covishield.

Supplementary Table 7: COVID-19 vaccine efficacy estimates in populations in which SARS-CoV-2 Gamma is circulating.

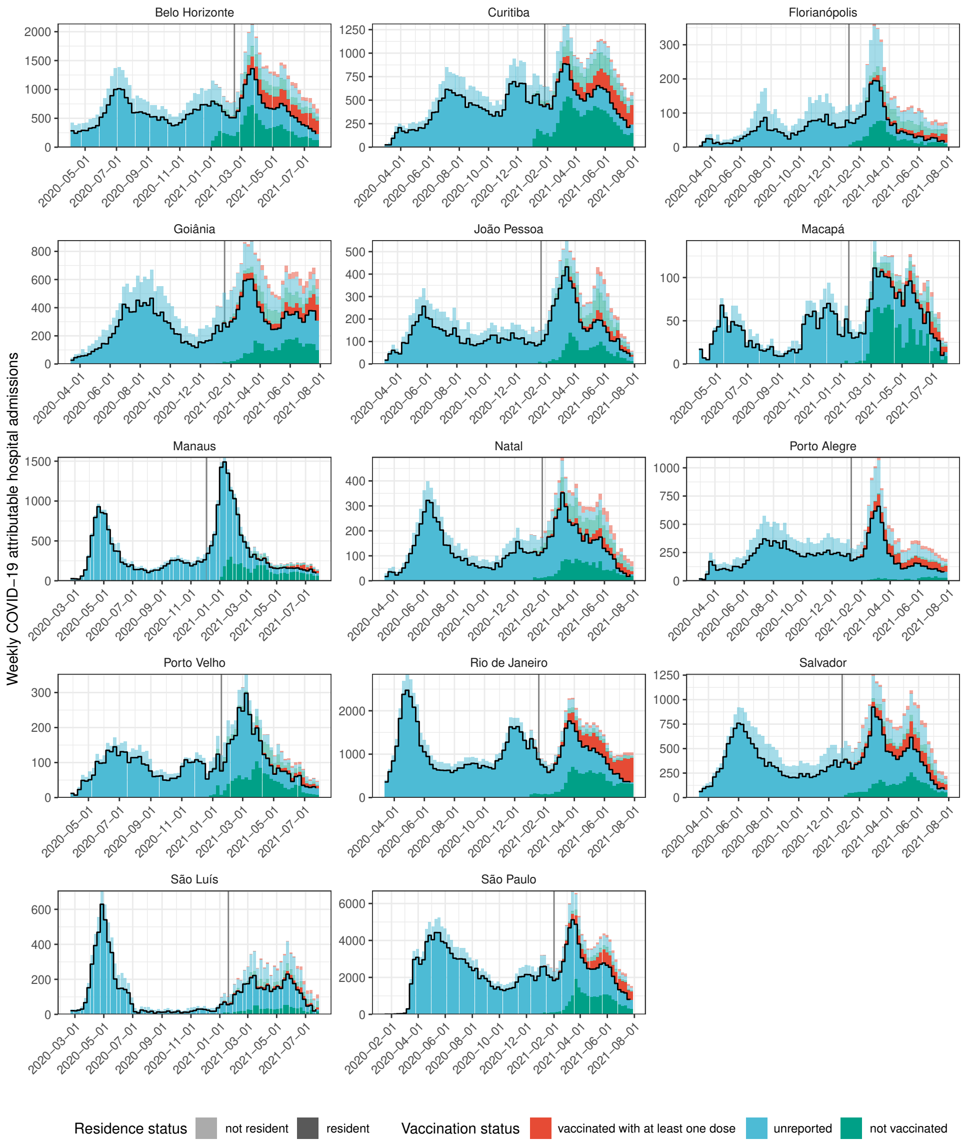

Supplementary Figure S1: COVID-19 attributable hospital admissions. Data are shown by week of admission and are stratified by residence status (lighter shade for non-residents and darker shade for residents) and vaccination status (colours) in 14 cities in Brazil. Data are from the SIVEP-Gripe platform of the Brazilian Ministry of Health and as of 31 January 2022. The black line indicates the hospitalised patients selected in this study. The grey vertical line indicates the date of Gamma's first detection in each city.

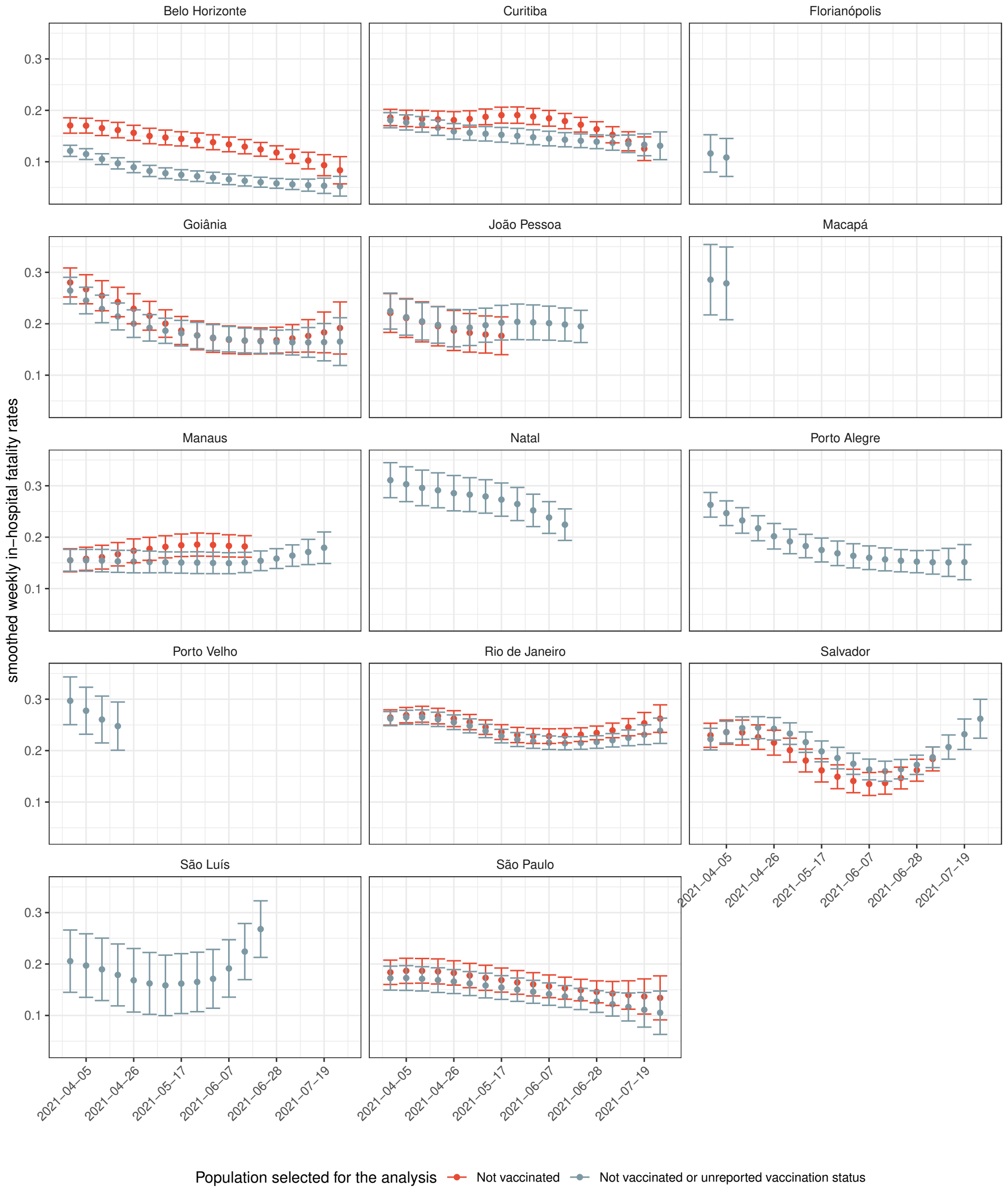

Supplementary Figure S2: Age-standardised COVID-19 fatality rates in reported unvaccinated patients only. From the denominator of COVID-19 attributable hospitalised residents, we further excluded patients with unreported vaccination status on or after March 29, 2021, and re-calculated the non-parametric, smoothed estimates of age-standardised COVID-19 fatality rates. To avoid extrapolation, we only included estimates obtained prior to the last date for which there was at least one hospital admission per age group. Mean estimates (dots) are shown along with 95% confidence intervals (errorbars) for two patient groups depending on reported vaccination status (colour): patients without evidence of vaccination (n=100,746) and confirmed unvaccinated patients (n=45,422)

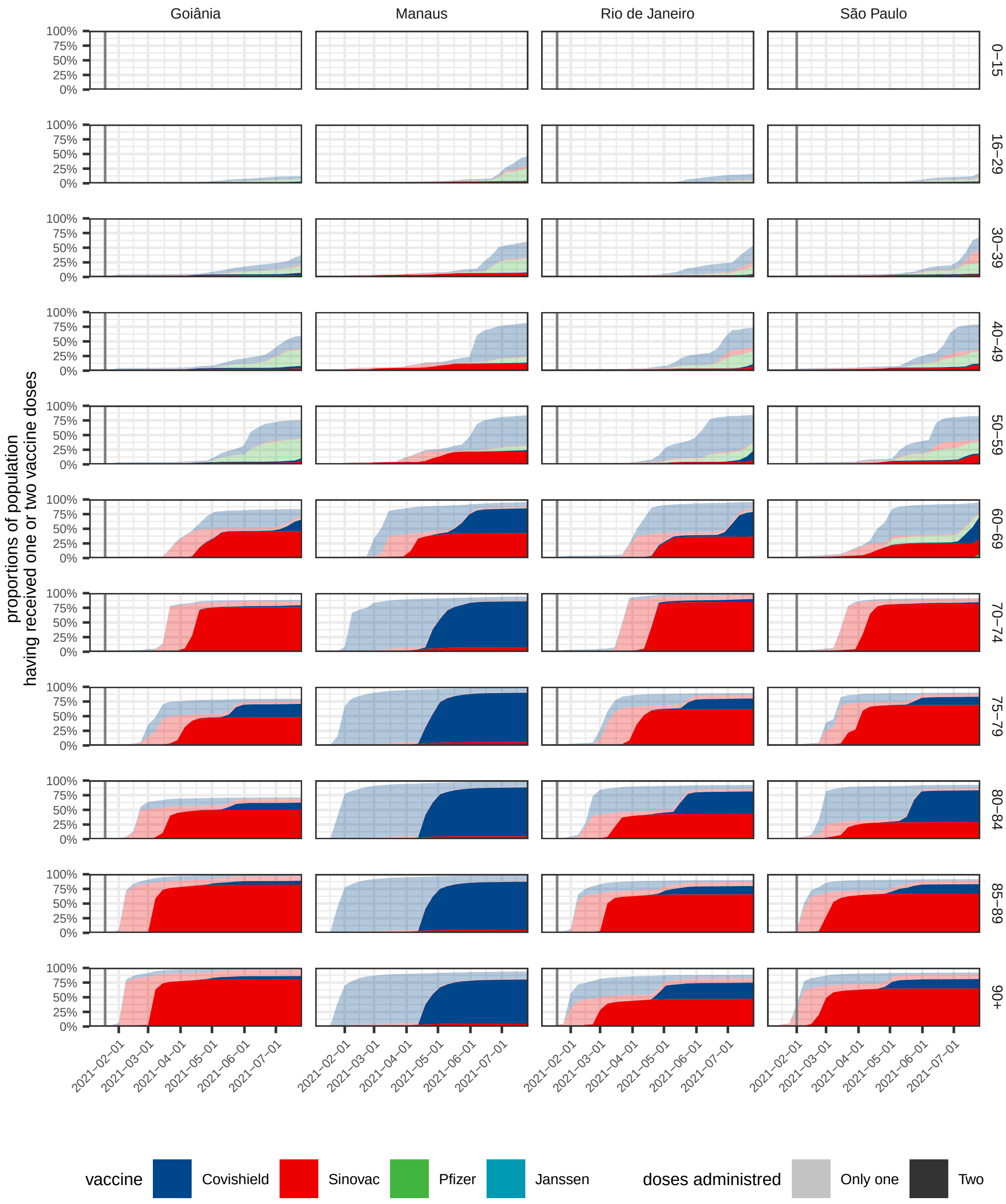

Supplementary Figure S3: COVID-19 vaccine coverage: Goiânia, Manaus, Rio de Janeiro and São Paulo. Individual-level data on administered vaccine doses from the Brazilian Ministry of Health database (https://opendatasus.saude.gov.br/dataset/covid-19-vacinacao) were retrieved on 5 August 2021 as described in the Methods. Estimated vaccine coverage is shown by vaccine (colour) and number of doses administered (colour intensity). The date of Gamma's detection is added as a grey vertical line.

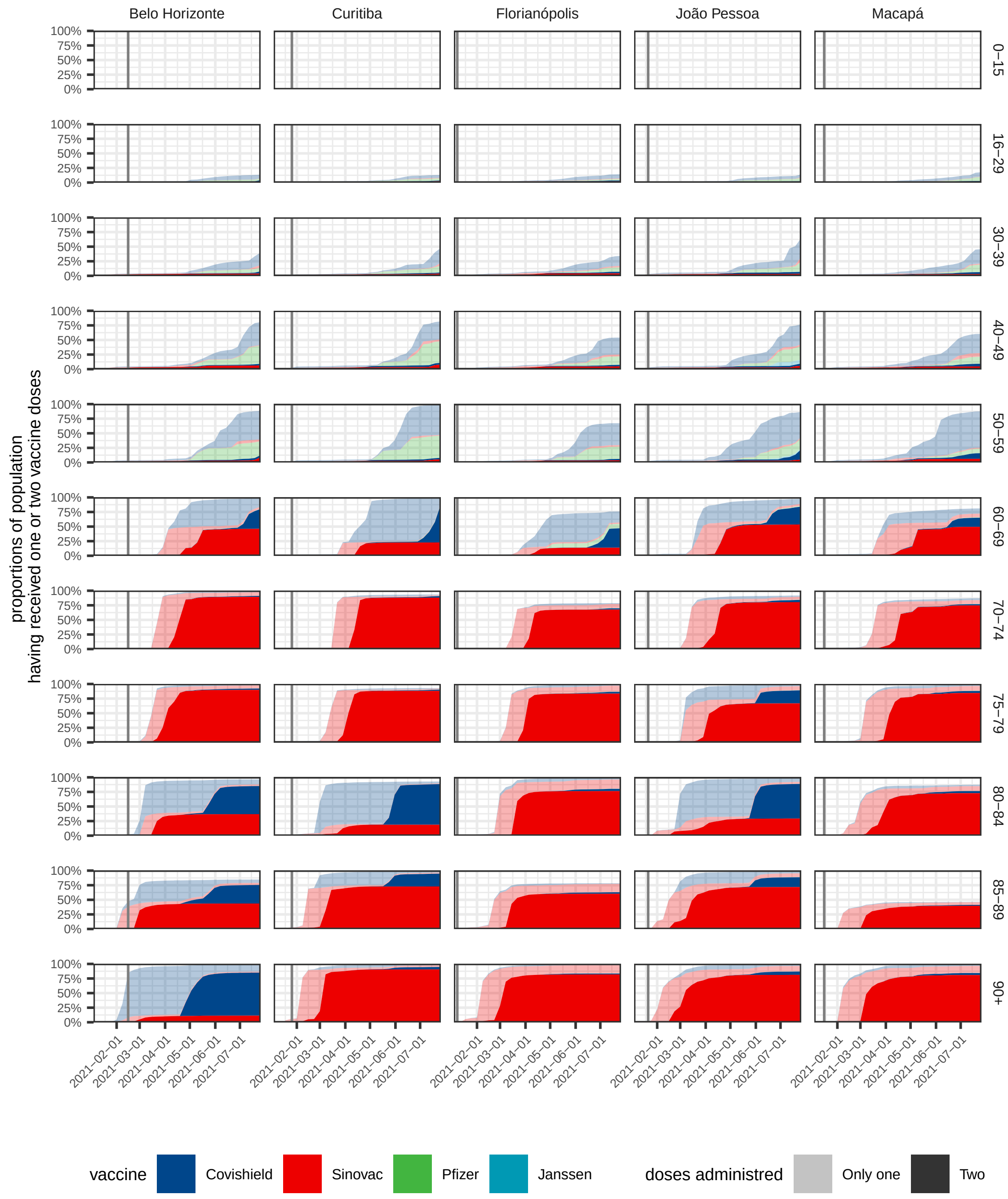

Supplementary Figure S4: COVID-19 vaccine coverage: Belo Horizonte, Curitiba, Florianópolis, João Pessoa and Macapá. Individual-level data on administered vaccine doses from the Brazilian Ministry of Health database (https://opendatasus.saude.gov.br/dataset/covid-19-vacinacao) were retrieved on 5 August 2021, and preprocessed as described in the Methods. Estimated vaccine coverage is shown by vaccine (colour) and number of doses administered (colour intensity). The date of Gamma's detection is added as a grey vertical line.

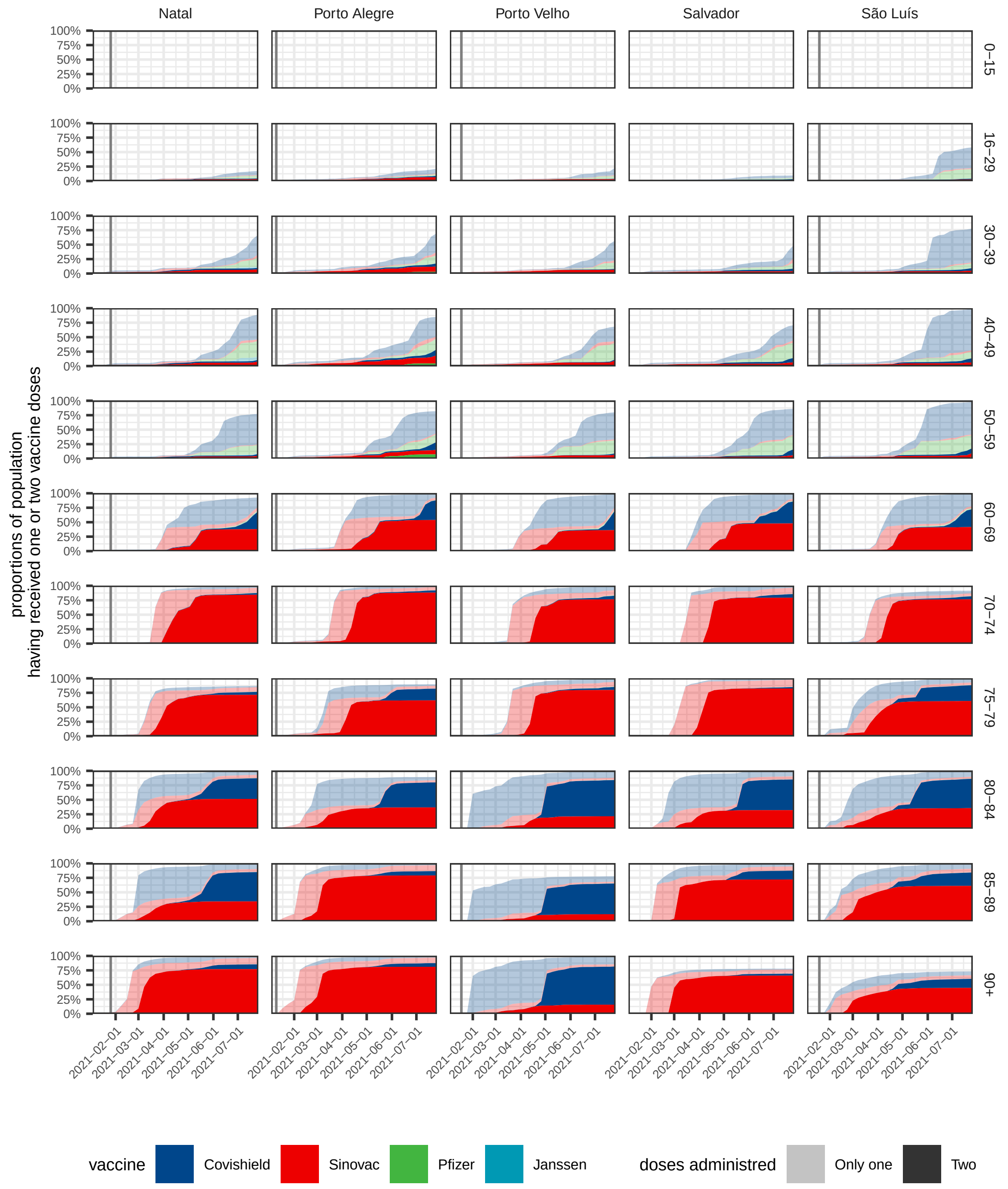

Supplementary Figure S5: COVID-19 vaccine coverage: Natal, Porto Alegre, Porto Velho, Salvador and São Luís. Individual-level data on administered vaccine doses from the Brazilian Ministry of Health database (https://opendatasus.saude.gov.br/dataset/covid-19-vacinacao) were retrieved on 5 August 2021, and preprocessed as described in the Methods. Estimated vaccine coverage is shown by vaccine (colour) and number of doses administered (colour intensity). The date of Gamma's detection is added as a grey vertical line.

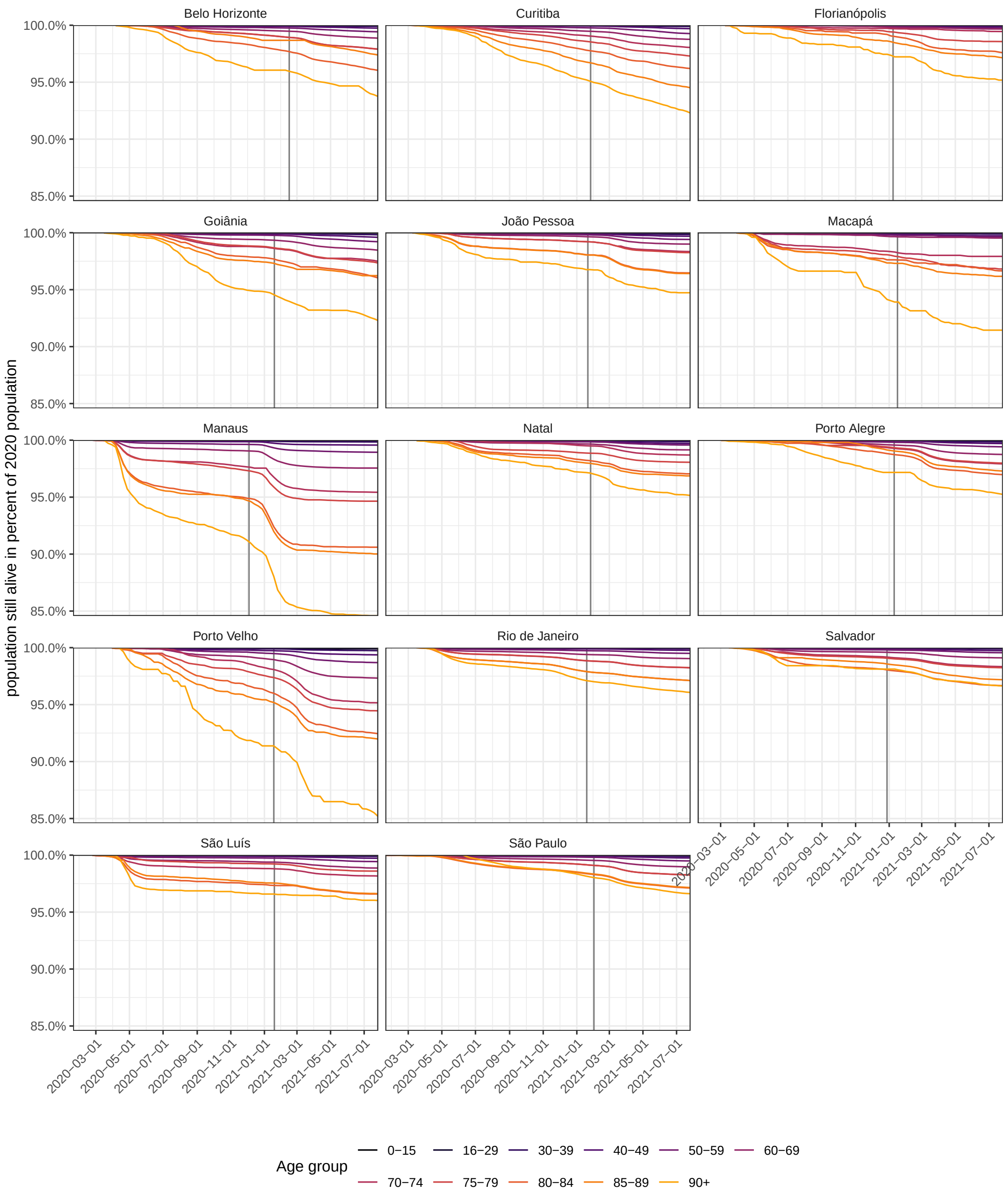

Supplementary Figure S6: Impact of age-specific cumulative mortality on population sizes. For each location, COVID-19 attributable deaths in residents were compared to excess deaths, and the larger of the two were cumulated over weeks for each age band. Shown is the proportion of the age-specific populations still alive by the end of each calendar week. The date of Gamma's detection is shown as grey vertical line.

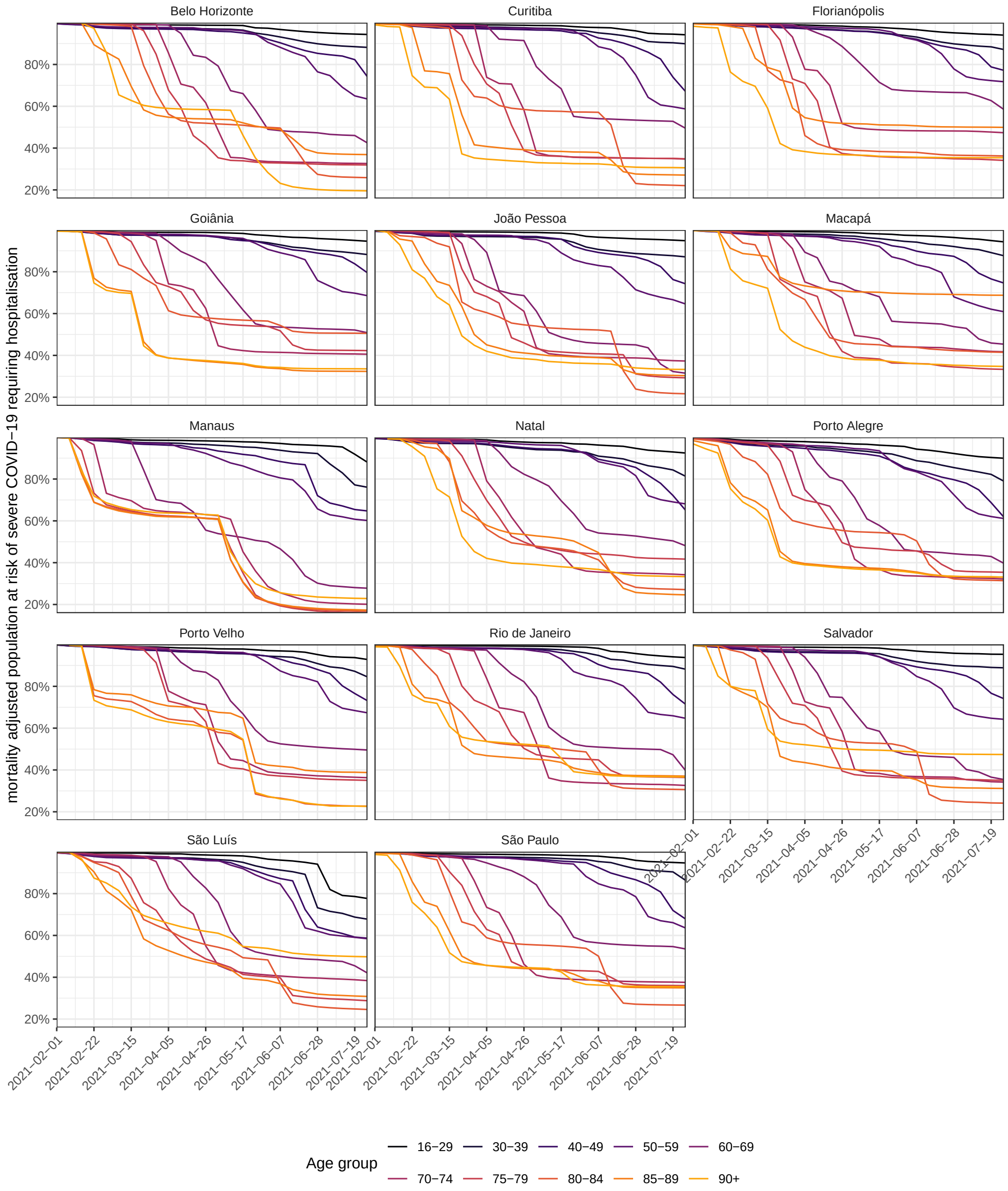

Supplementary Figure S7: Impact of age-specific vaccination rates on population at risk of severe COVID-19 infection. Individual vaccine administration records were for each city aggregated by vaccine, number of doses, week and age band. We then subtracted from the mortality adjusted population size projections a proportion of the vaccinated sub-populations in each age band and week according to the COVID-19 vaccine efficacy estimates in Supplementary Table 7 to estimate the population protected from severe COVID-19 infection and hospitalisation. Shown are the proportions of the mortality adjusted populations in each age band that are estimated to be at risk of severe COVID-19 infection and hospitalisation.

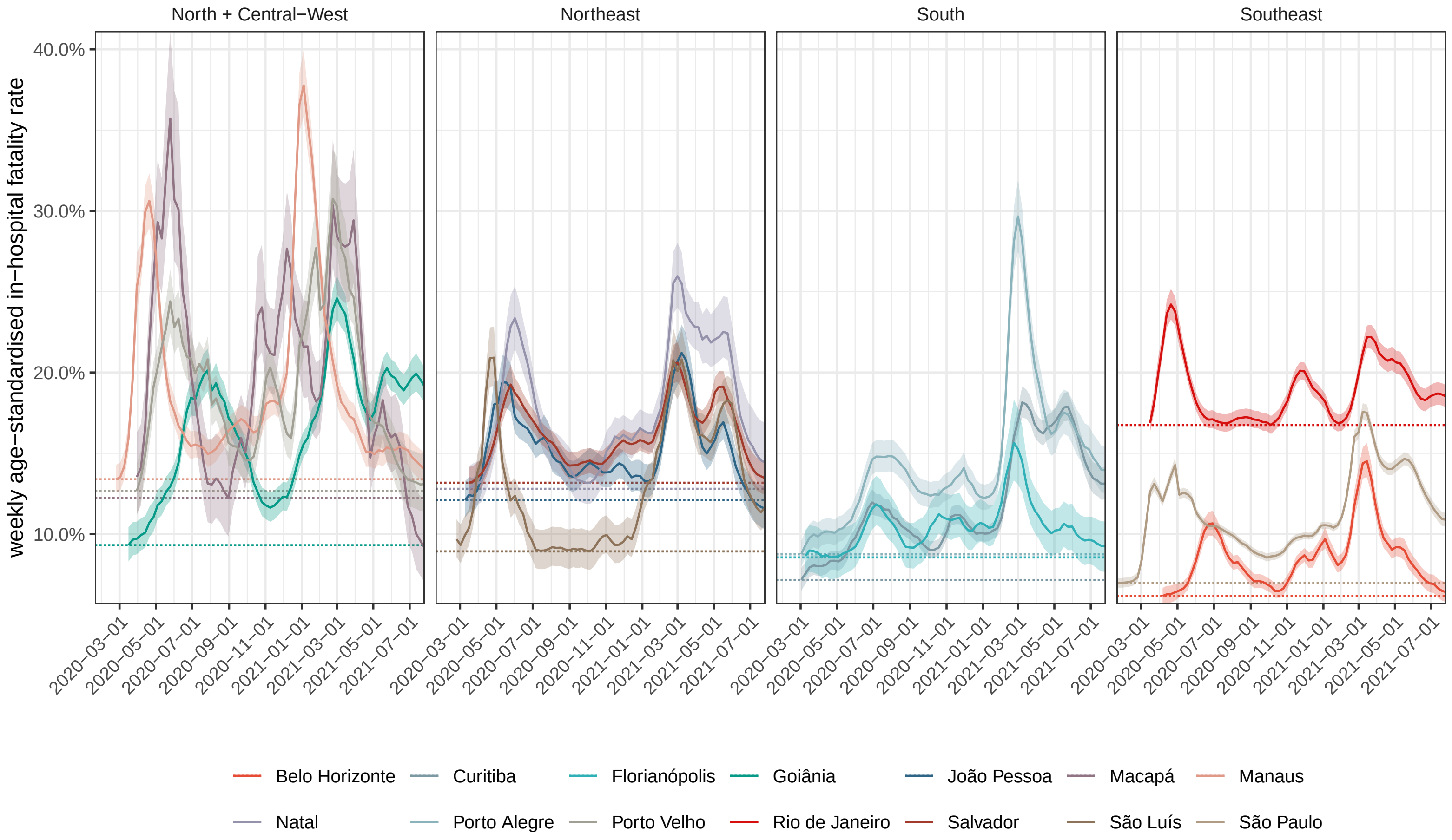

Supplementary Figure S8: Estimated, weekly age-standardised COVID-19 in-hospital fatality rates assuming that all hospitalisations with unreported outcome were discharged. Posterior median estimates (line) are shown with 95%CrIs (ribbon), and the lowest estimated fatality rates during the observation period in each state capital (dotted horizontal line).

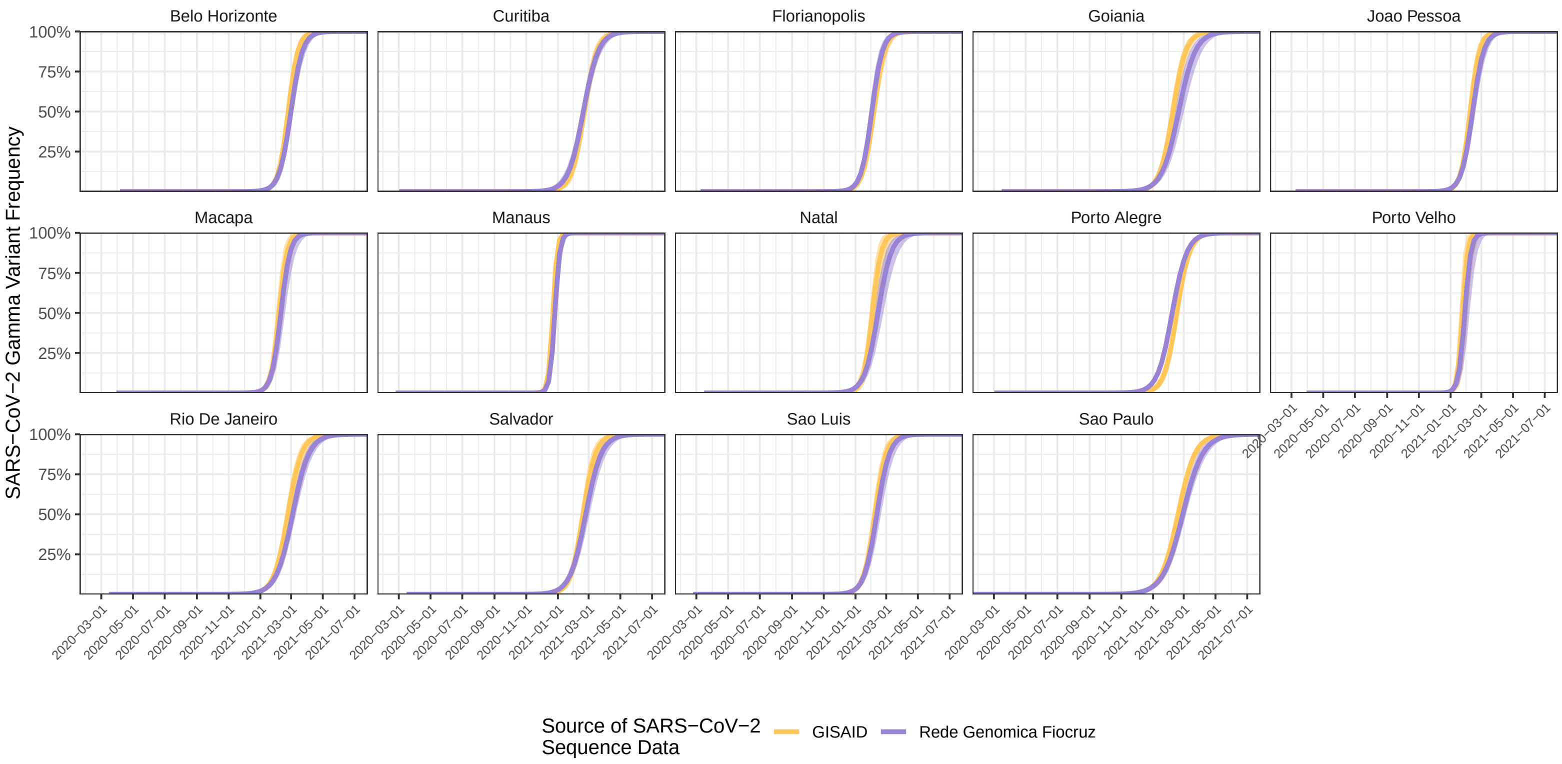

Supplementary Figure S9: Estimated temporal dynamics of Gamma frequency across the 14 cities when using sequence data from Rede Genômica FioCruz. Posterior median estimates (dots) are shown along with 95% credible intervals (linerange) for the central analysis (yellow, n=7,221 samples) and sensitivity analysis (purple, n=17,070 samples).

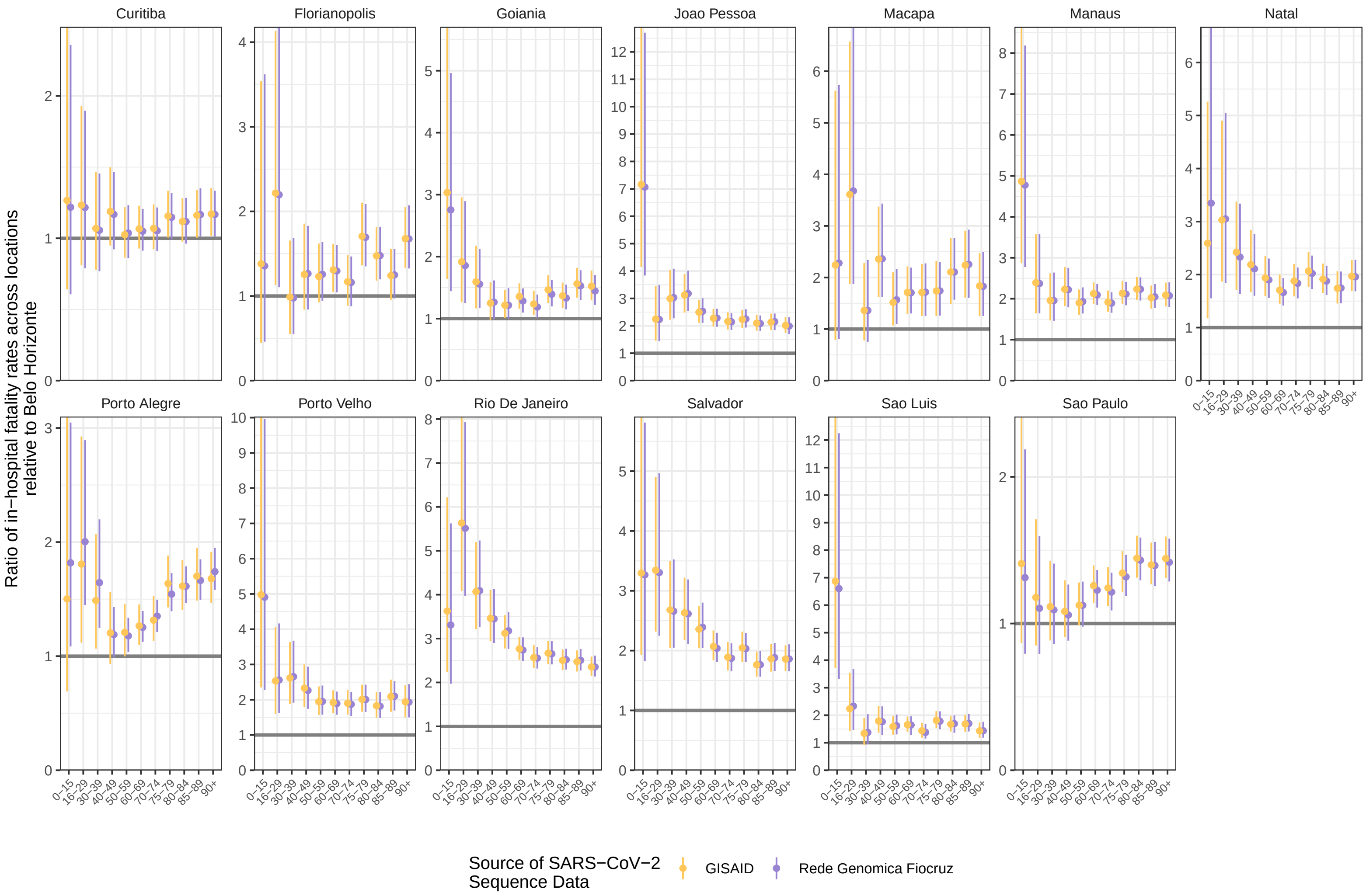

Supplementary Figure S10: Estimated effect of location on in-hospital fatality rates when using sequence data from Rede Genômica FioCruz. Posterior median estimates (dots) are shown along with 95% credible intervals (linerange) for the central analysis (yellow, n=7,221 samples) and sensitivity analysis (purple, n=17,070 samples).

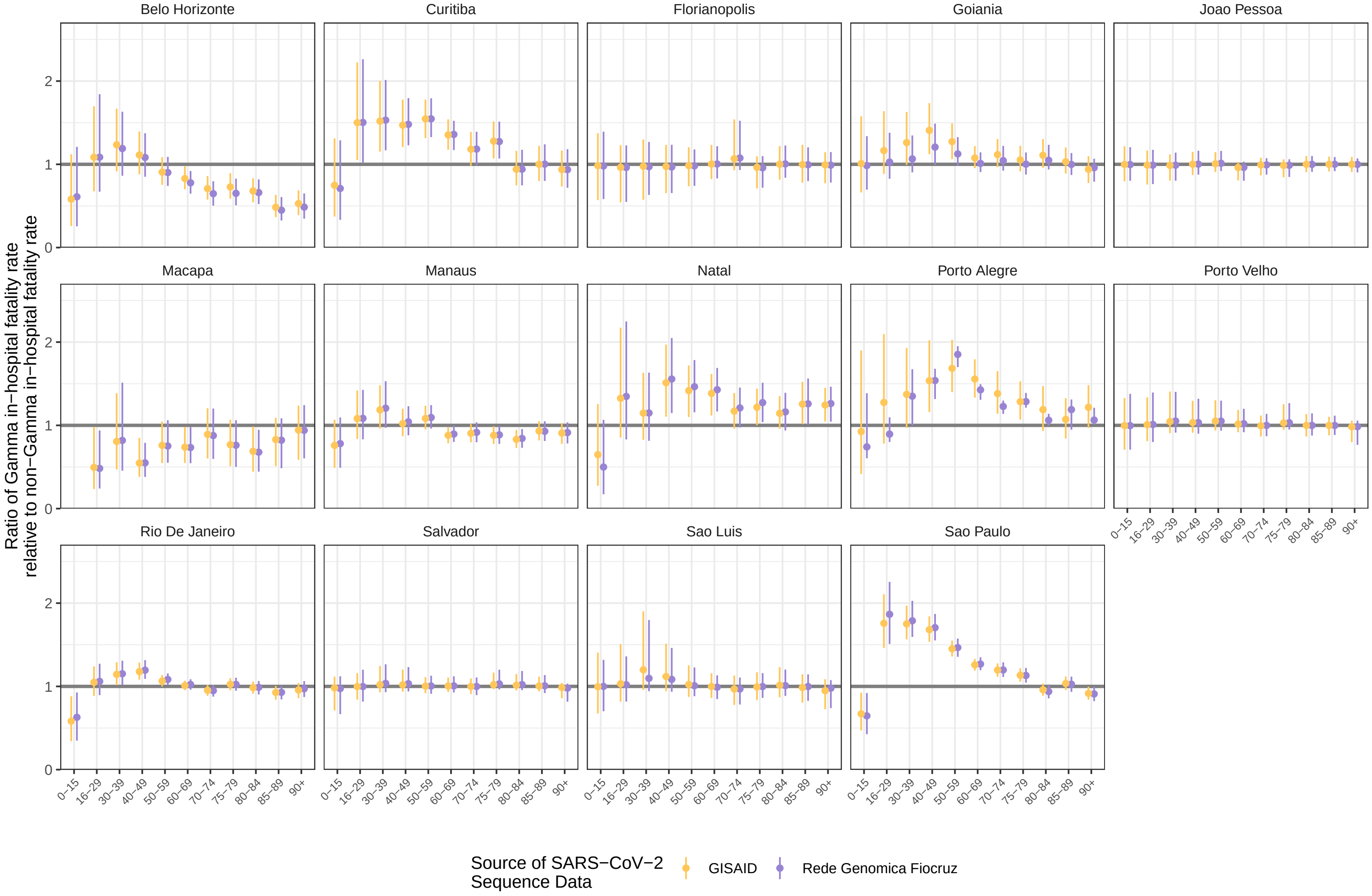

Supplementary Figure S11: Estimated effect of Gamma infection on in-hospital fatality rates when using sequence data from Rede Genômica FioCruz. Posterior median estimates (dots) are shown along with 95% credible intervals (linerange) for the central analysis (yellow, n=7,221 samples) and sensitivity analysis (purple, n=17,070 samples).

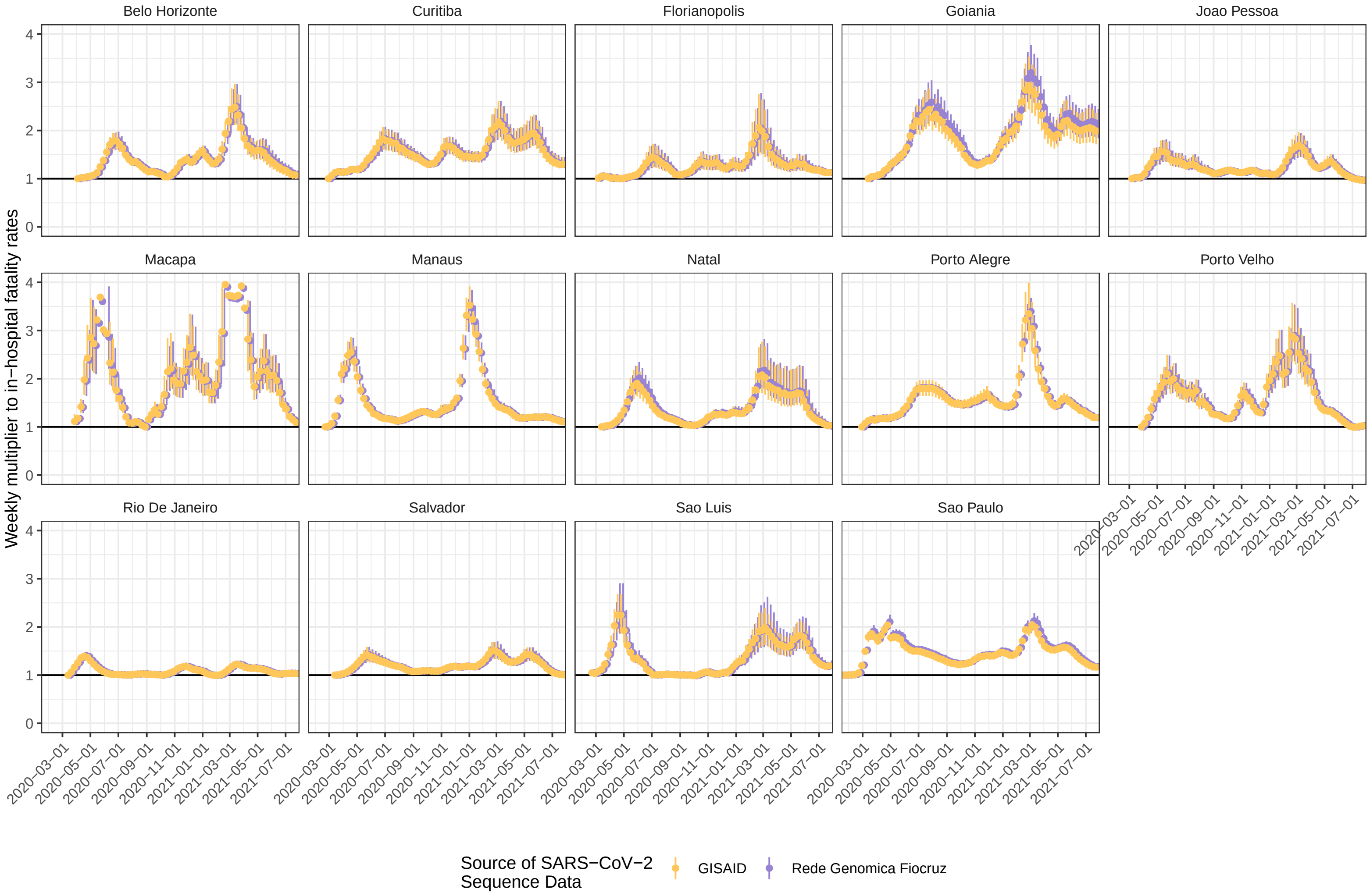

Supplementary Figure S12: Estimated effect of healthcare pressure on in-hospital fatality rates when using sequence data from Rede Genômica FioCruz. Posterior median estimates (dots) are shown along with 95% credible intervals (linerange) for the central analysis (yellow, n=3,683 samples) and sensitivity analysis (purple, n=301 samples).

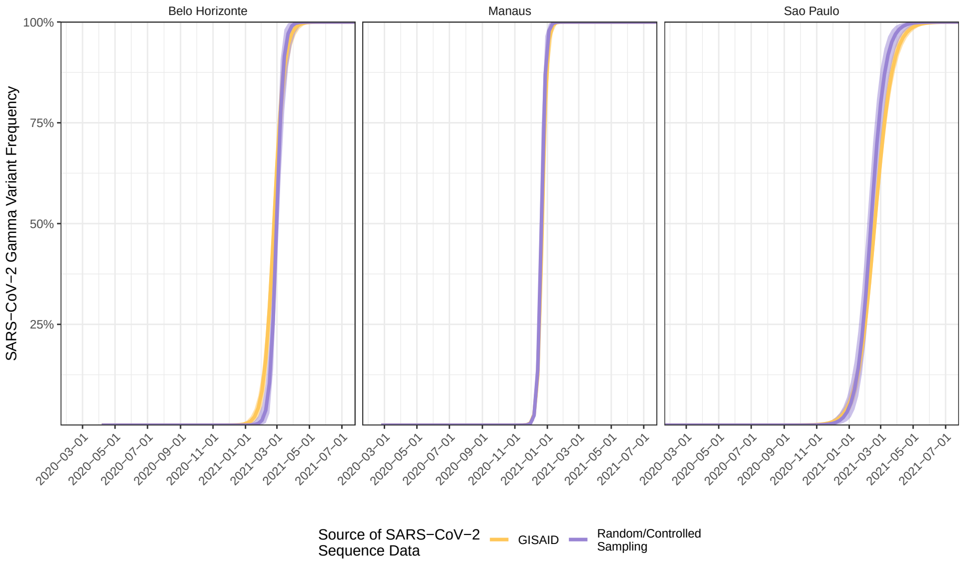

Supplementary Figure S13: Estimated temporal dynamics of Gamma frequency in when using sequence data collected using controlled sampling methodologies. Posterior median estimates (dots) are shown along with 95% credible intervals (linerange) for the central analysis (yellow) and sensitivity analysis (purple).

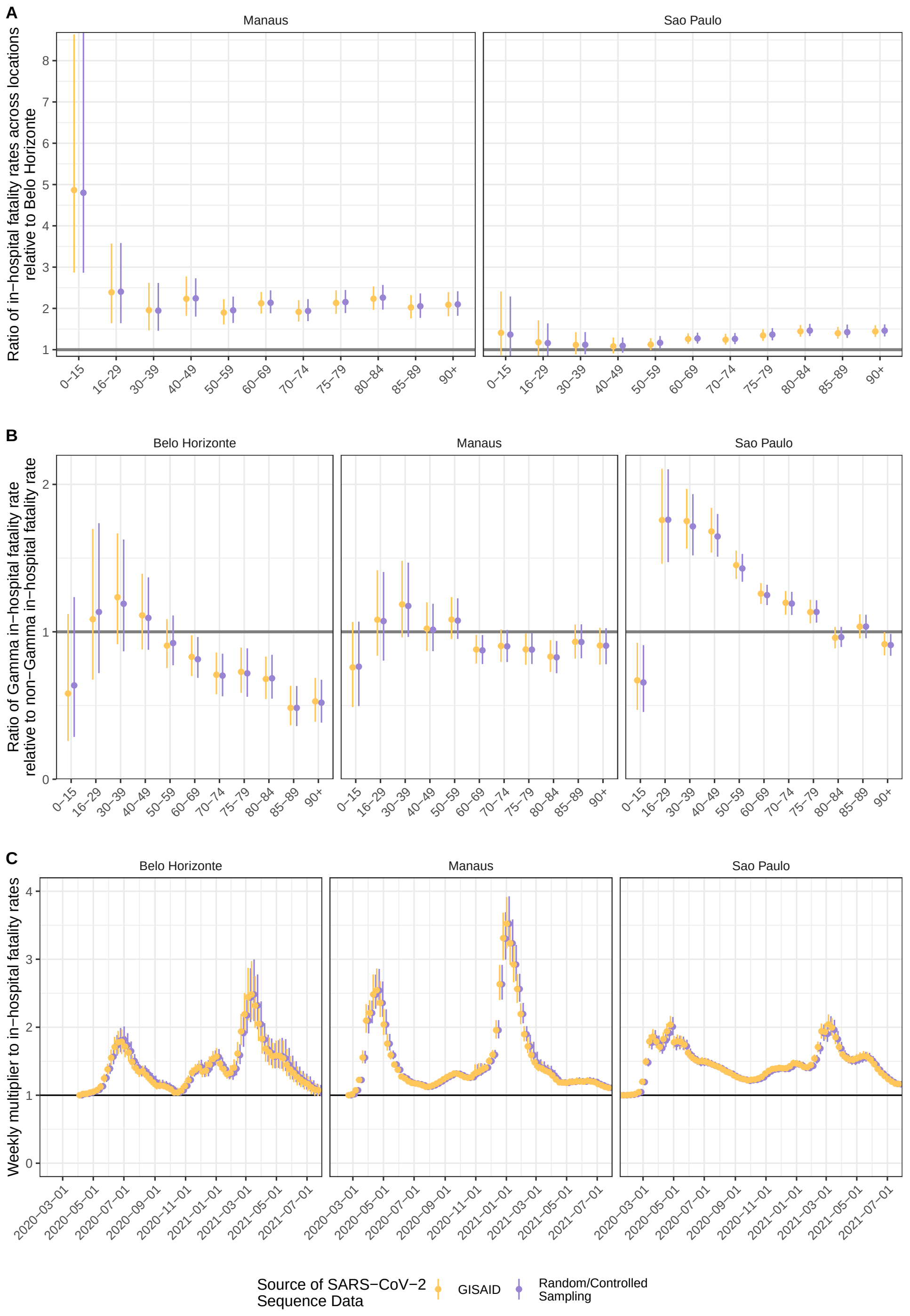

Supplementary Figure S14: Estimated location, Gamma and healthcare pressure effects when using sequence data collected using controlled sampling methodologies. (A) Estimated location effect, (B) Estimated Gamma effect. (C) Estimated healthcare pressure effect. Posterior median estimates (dots) are shown along with 95% credible intervals (linerange) for the central analysis (yellow) and sensitivity analysis (purple).

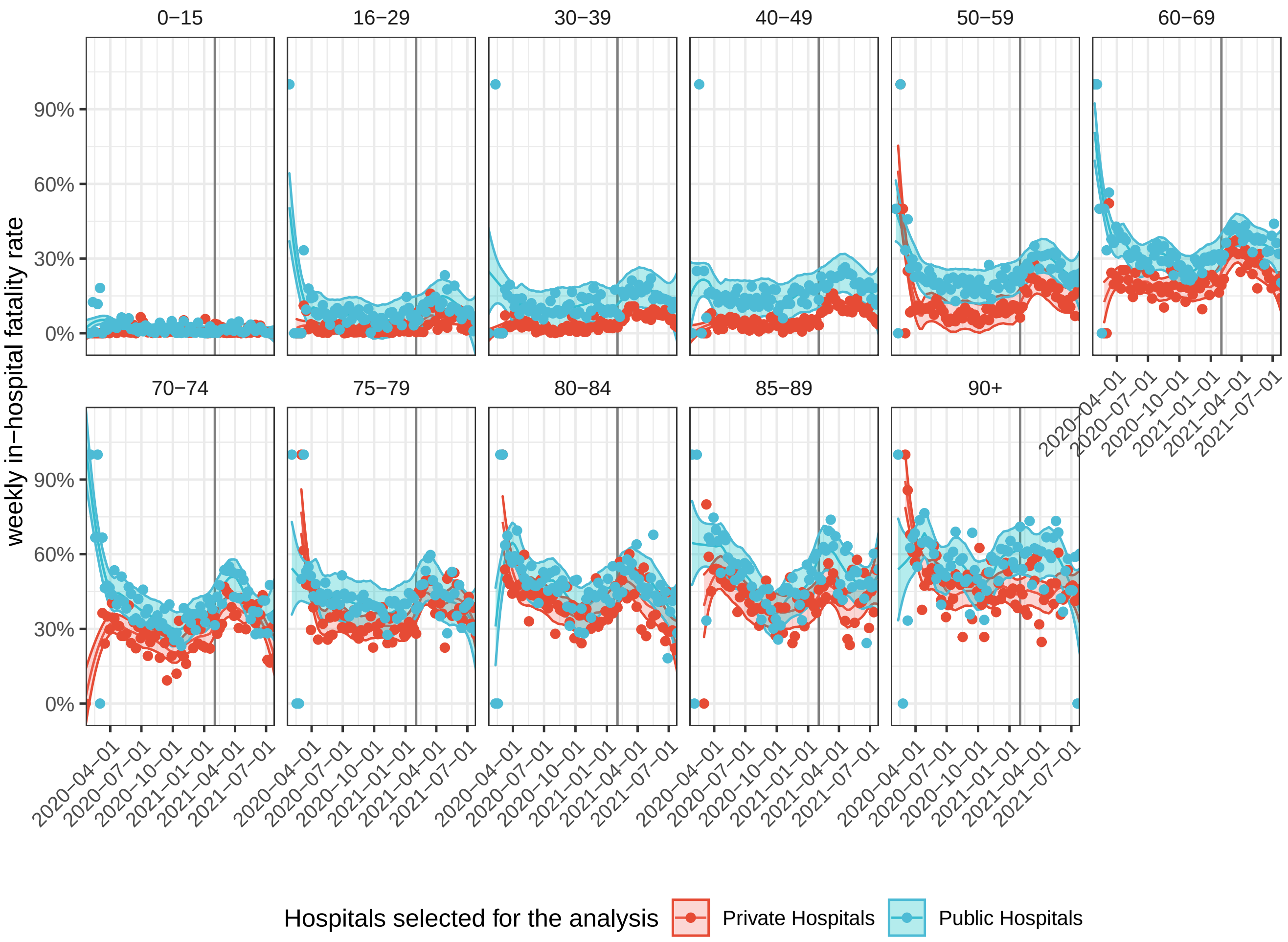

Supplementary Figure S15: Non-parametric estimates of COVID-19 in-hospital fatality rates in public and private hospitals by age group in São Paulo. Nearly all of São Paulo's hospitals could be categorised into public or private healthcare facilities, and COVID-19 in-hospital fatality rates were calculated among unvaccinated patients in public hospitals (blue), and private hospitals (red). Non-parametric, smoothed loess mean estimates (line) are shown with 95% confidence intervals (ribbon). Weekly data are shown as dots, and the date of Gamma's first detection is indicated as a vertical dotted black line.

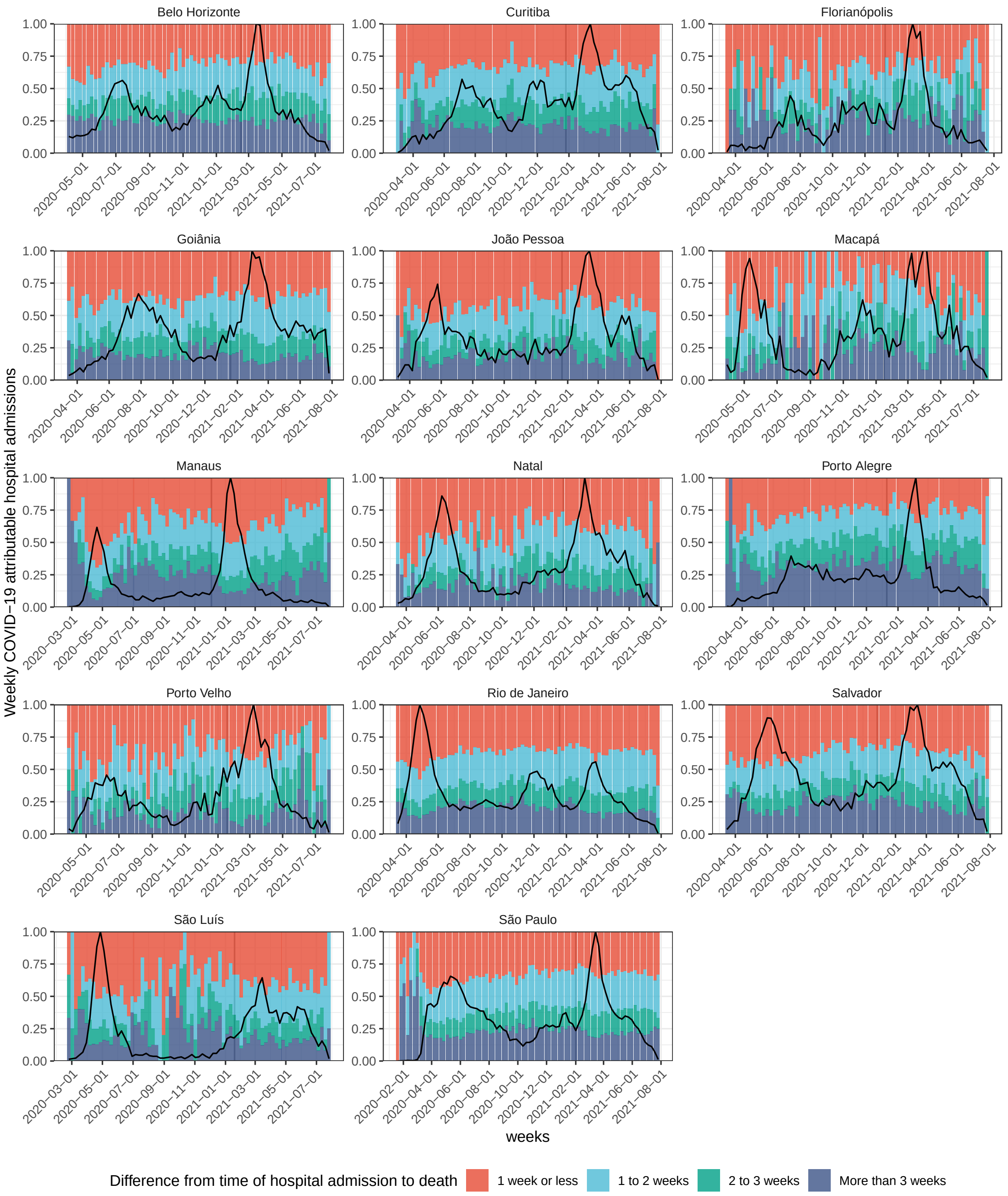

Supplementary Figure S16: Time to death in COVID-19 attributable hospital admissions. Fatalities in weekly COVID-19 attributable hospital admissions were stratified by time to death (1 week or less, 1-2 weeks, 2-3 weeks or more than 3 weeks since admission). We show the proportion of deaths in each time to death stratum (colours). Data for each city are in facets. For reference, the time evolution of weekly hospital admissions is shown with the black line.

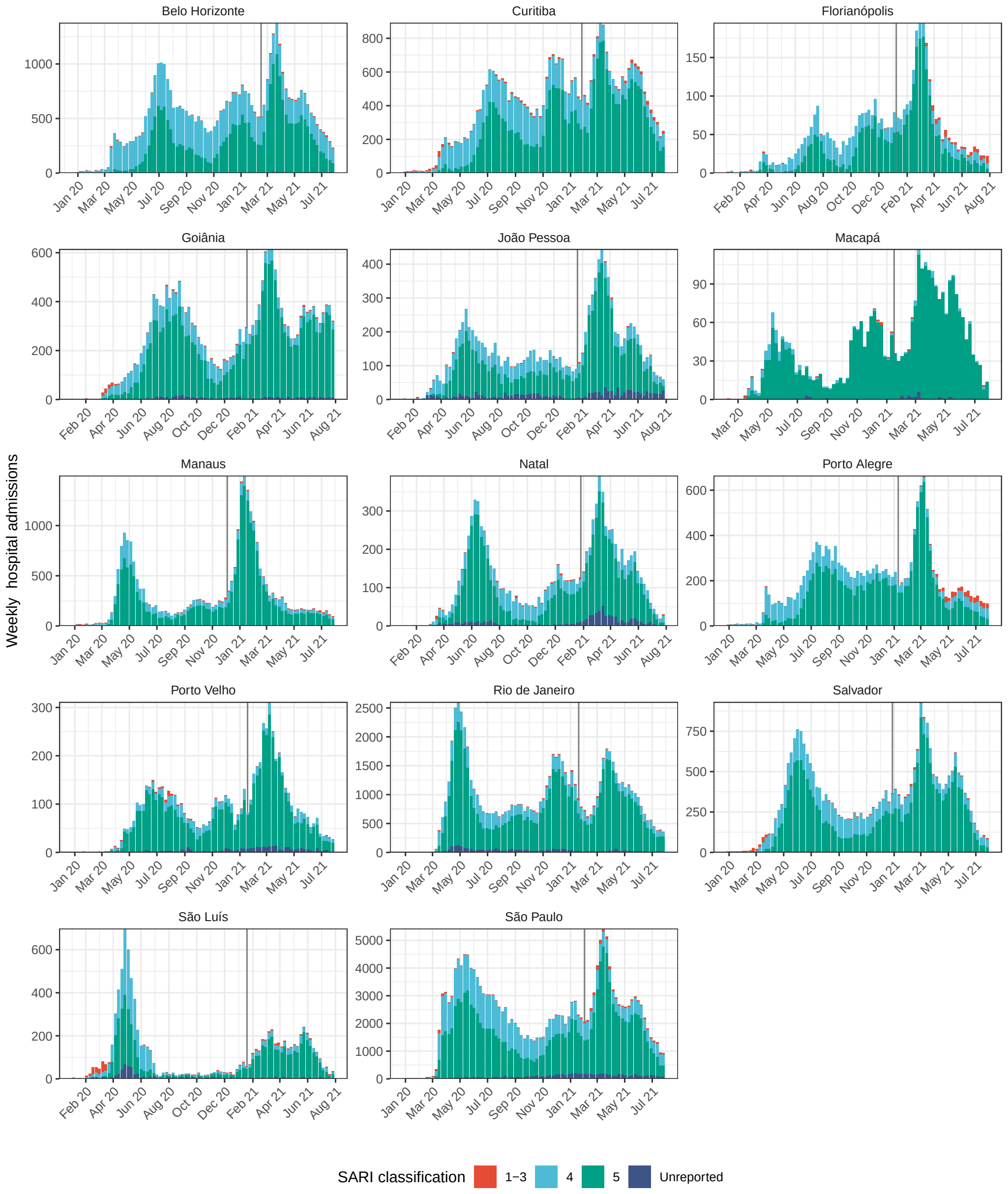

Supplementary Figure S17: Weekly hospital admissions with severe acute respiratory infections in residents without evidence of prior COVID-19 vaccination. Data are shown by week of admission and are stratified by the SIVEP-Gripe classification of severe acute respiratory infections. Classes 1-3 comprise patients with confirmed infection with pathogens other than SARS-CoV-2. Class 4 comprises patients with suspected COVID-19. Class 5 comprises patients with confirmed COVID-19. Patients with unreported diagnosis are shown separately. Data are from the SIVEP-Gripe platform of the Brazilian Ministry of Health and as of 31 January 2022. The grey vertical line indicates the date of Gamma's first detection in each city.

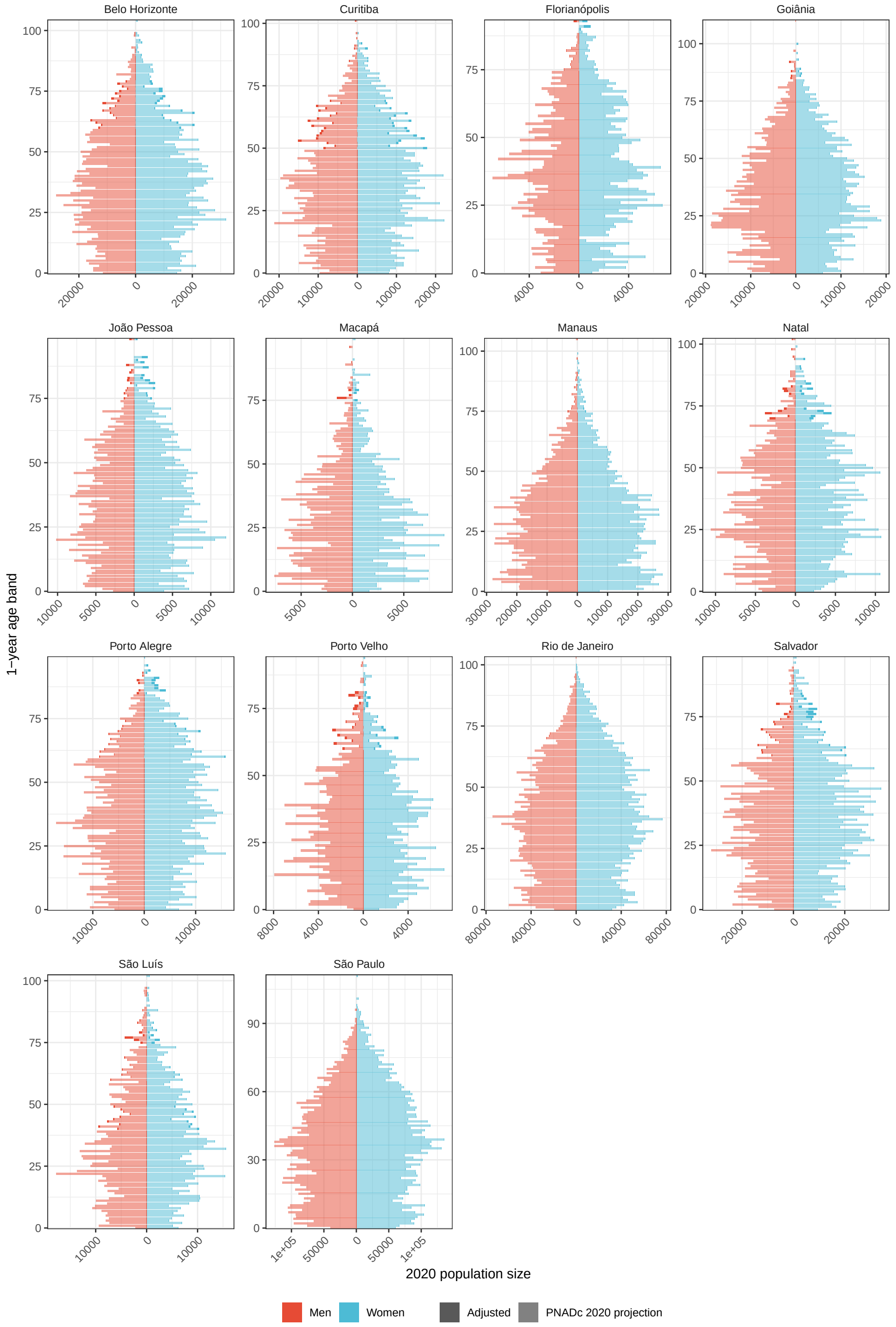

Supplementary Figure S18: 2020 population size projections by sex and 1-year age bands across state capitals and the Federal District. The population size projections were retrieved from the PNAD COVID-19 survey and adjusted to be compatible with vaccine administration records (see Methods).

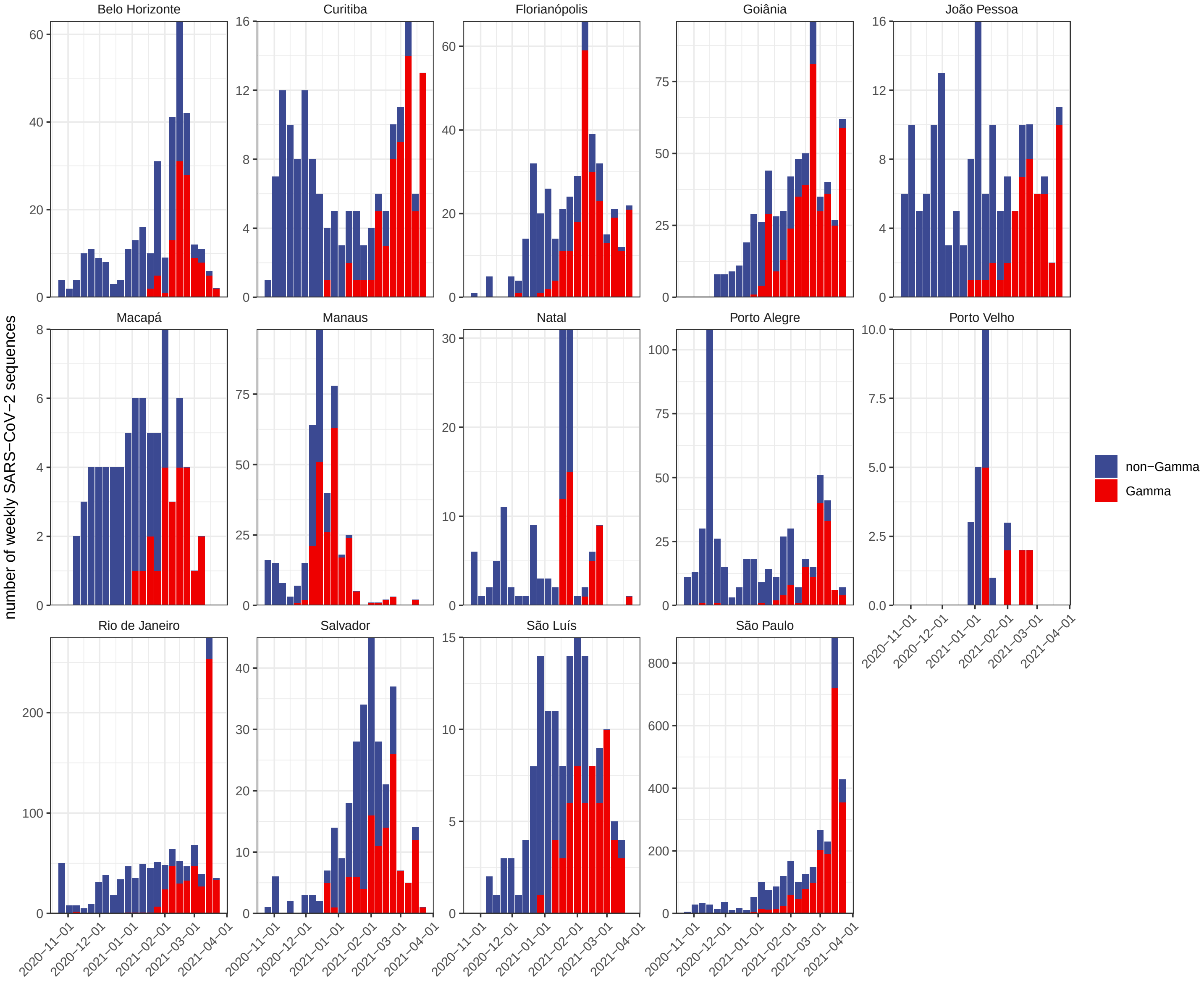

Supplementary Figure S19: SARS-CoV-2 sequence data obtained from GISAID in the 14 states in which the 14 state capitals are located.
