# Supplementary material for: Spatial and temporal fluctuations in COVID-19 fatality rates in Brazilian hospitals
Source: Nat Med. 2022 May 10;28(7):1476–85. doi: 10.1038/s41591-022-01807-1 (PMC9307484; doi:10.1038/s41591-022-01807-1)
Supplement: Supplementary file 2 — Reporting Summary [file 41591_2022_1807_MOESM2_ESM.pdf]

nature portfolio

Corresponding author(s): Oliver Ratmann, Samir Bhatt, Nuno Faria, Ester Sabino

Last updated by author(s): Mar 28, 2022

Reporting Summary

Nature Portfolio wishes to improve the reproducibility of the work that we publish. This form provides structure for consistency and transparency in reporting. For further information on Nature Portfolio policies, see our Editorial Policies and the Editorial Policy Checklist.

Statistics

For all statistical analyses, confirm that the following items are present in the figure legend, table legend, main text, or Methods section.

n/a	Confirmed	
☐	☒	The exact sample size (*n*) for each experimental group/condition, given as a discrete number and unit of measurement
☒	☐	A statement on whether measurements were taken from distinct samples or whether the same sample was measured repeatedly
☒	☐	The statistical test(s) used AND whether they are one- or two-sided<br>*Only common tests should be described solely by name; describe more complex techniques in the Methods section.*
☐	☒	A description of all covariates tested
☐	☒	A description of any assumptions or corrections, such as tests of normality and adjustment for multiple comparisons
☐	☒	A full description of the statistical parameters including central tendency (e.g. means) or other basic estimates (e.g. regression coefficient) AND variation (e.g. standard deviation) or associated estimates of uncertainty (e.g. confidence intervals)
☒	☐	For null hypothesis testing, the test statistic (e.g. *F*, *t*, *r*) with confidence intervals, effect sizes, degrees of freedom and *P* value noted<br>*Give P values as exact values whenever suitable.*
☐	☒	For Bayesian analysis, information on the choice of priors and Markov chain Monte Carlo settings
☒	☐	For hierarchical and complex designs, identification of the appropriate level for tests and full reporting of outcomes
☐	☒	Estimates of effect sizes (e.g. Cohen's *d*, Pearson's *r*), indicating how they were calculated

Our web collection on statistics for biologists contains articles on many of the points above.

Software and code

Policy information about availability of computer code

Data collection

No primary data collection was carried out for these analyses.

Data analysis

All code used for the analyses is publicly available at: https://github.com/CADDE-CENTRE/covid19_brazil_hfr/ (DOI:10.5281/zenodo.6373425).
The following packages were used within R version 4.0.3: (cmdstan v0.3.0.9, stats v4.0.3). Software requirements to reproduce the statistical analysis can be found at `covid19_brazil_hfr.yml` in our github directory.
Pangolin v.3.0.6, Pangolearn v.1.2.12, scorpio lineage (https://github.com/cov-lineages/pangolin), BEAST v0.10.4 (https://github.com/beast-dev/beast-mcmc/releases/tag/v1.10.4) and v1.10.5 (https://github.com/beast-dev/beast-mcmc/releases/tag/v1.10.5pre_thorney_v0.1.2), MAFFT, version 7(https://mafft.cbrc.jp/alignment/software/source.html), TempEst v.1.5.3. (http://tree.bio.ed.ac.uk/software/tempest), Tracer v.1.7(https://github.com/beast-dev/tracer/releases/tag/v1.7) and the ARTIC bioinformatics pipeline (https://github.com/articnetwork/fieldbioinformatics) were used for phylogenetic analyses.
Further, we used the TreeMarkovJumpHistoryAnalyzer tool, available at `inst/utils/P.1_MJumps_complete_history.xml` in our github directory.

For manuscripts utilizing custom algorithms or software that are central to the research but not yet described in published literature, software must be made available to editors and reviewers. We strongly encourage code deposition in a community repository (e.g. GitHub). See the Nature Portfolio guidelines for submitting code & software for further information.

Data

Policy information about availability of data

All manuscripts must include a data availability statement. This statement should provide the following information, where applicable:

- Accession codes, unique identifiers, or web links for publicly available datasets
- A description of any restrictions on data availability
- For clinical datasets or third party data, please ensure that the statement adheres to our policy

All source code and data necessary for the replication of our results are available at https://github.com/CADDE-CENTRE/covid19_brazil_hfr (DOI:10.5281/zenodo.6373425).

These datasets were derived from the following public domain resources: the SIVEP-Gripe platform https://opendatasus.saude.gov.br/dataset/srag-2020, https://opendatasus.saude.gov.br/dataset/srag-2021-e2022; the Brazilian Civil Registry https://transparencia.registrocivil.org.br/; the Brazilian Ministry of Health; https://opendatasus.saude.gov.br/dataset/covid-19-vacinacao; the National Household Sample Survey COVID-19 https://www.ibge.gov.br/estatisticas/sociais/populacao/9171-pesquisa-nacional-poramostra-de-domicilios-continua-mensal.html?=&t=o-que-e; and Brazil's National Register of Health Facilities https://datasus.saude. gov.br/transferencia-de-arquivos/
Data from the Brazilian Civil Registry was accessed on the 9th of August through https://github.com/capyvara/brazil-civil-registry-data.
The downloaded and processed versions are also available in our Github repository at:inst/data/SIVEP_hospital_31-01-2022-all.rds; inst/data/registry_covid_detailed_09-08-2021.csv; inst/data/aggregated_vaccinations_210805.rds; inst/data/PNADc_populationpyramids_210617.csv; inst/data/genomic_data_210702.csv; inst/data/IPEA_ICUbeds_physicians_210928.csv

Field-specific reporting

Please select the one below that is the best fit for your research. If you are not sure, read the appropriate sections before making your selection.

☒ Life sciences ☐ Behavioural & social sciences ☐ Ecological, evolutionary & environmental sciences

For a reference copy of the document with all sections, see nature.com/documents/nr-reporting-summary-flat.pdf

Life sciences study design

All studies must disclose on these points even when the disclosure is negative.

Sample size	Sample sizes for our datasets were calculated as the number of Severe Acute Respiratory Infections (SARI) reported to the SIVEP-Gripe platform satisfying different criteria. Sample sizes are reported in the Introduction of the main text and schematized in Figure 1.
Data exclusions	Criteria for inclusion of each reported SARI case in our datasets are discussed in the Introduction of the main text. Briefly, we excluded:<br>- patients confirmed with respiratory pathogens other than SARS-CoV-2, or with unreported cause as our focus was COVID-19 severity.<br>- non-resident patients, to preserve the same population denominators as in the population size estimates;<br>- patients with proof of vaccine administration, to avoid confounding of time trends in fatality rates and vaccine rollout;<br>The above exclusion criteria were pre-established.
Replication	Analyses were performed independently for each city, and thus estimates from each location provide independent support into the inferred relationships between in-hospital fatality rates, healthcare inequities, and healthcare pressure.
Randomization	The findings are derived from a retrospective longitudinal observational study on fatality rates in hospitalised patients and as such randomisation was infeasible.
Blinding	All data sources are publicly and freely available.

Reporting for specific materials, systems and methods

We require information from authors about some types of materials, experimental systems and methods used in many studies. Here, indicate whether each material, system or method listed is relevant to your study. If you are not sure if a list item applies to your research, read the appropriate section before selecting a response.

Materials & experimental systems

n/a	Involved in the study
☒	☐ Antibodies
☒	☐ Eukaryotic cell lines
☒	☐ Palaeontology and archaeology
☒	☐ Animals and other organisms
☒	☐ Human research participants
☒	☐ Clinical data
☒	☐ Dual use research of concern

Methods

n/a	Involved in the study
☒	☐ ChIP-seq
☒	☐ Flow cytometry
☒	☐ MRI-based neuroimaging
